# SIRT5 deficiency suppresses mitochondrial ATP production and promotes AMPK activation in response to energy stress

**DOI:** 10.1371/journal.pone.0211796

**Published:** 2019-02-13

**Authors:** Mengli Zhang, Jian Wu, Renqiang Sun, Xiaoting Tao, Xiaoxia Wang, Qi Kang, Hui Wang, Lei Zhang, Peng Liu, Jinye Zhang, Yukun Xia, Yuzheng Zhao, Yi Yang, Yue Xiong, Kun-Liang Guan, Yunzeng Zou, Dan Ye

**Affiliations:** 1 Key Laboratory of Metabolism and Molecular Medicine, Ministry of Education, and The Molecular and Cell Biology Lab, Institutes of Biomedical Sciences, Key Laboratory of Medical Epigenetics and Metabolism, Shanghai Medical College, Fudan University, Shanghai, China; 2 Shanghai Institute of Cardiovascular Diseases, Zhongshan Hospital and Institutes of Biomedical Sciences, Fudan University, Shanghai, China; 3 Department of Thoracic Surgery and Department of Oncology, Shanghai Medical College, Fudan University, Shanghai, China; 4 Waters corporation Shanghai Science & Technology Co Ltd, Shanghai, China; 5 School of Pharmacy, East China University of Science and Technology, Shanghai, China; 6 Lineberger Comprehensive Cancer Center, Department of Biochemistry and Biophysics, University of North Carolina at Chapel Hill, Chapel Hill, North Carolina, United States; 7 Department of Pharmacology and Moores Cancer Center, University of California San Diego, La Jolla, California, United States; 8 Department of General Surgery, Huashan Hospital, Fudan University, Shanghai, China; University of Cincinnati College of Medicine, UNITED STATES

## Abstract

Sirtuin 5 (SIRT5) is a member of the NAD^+^-dependent sirtuin family of protein deacylase that catalyzes removal of post-translational modifications, such as succinylation, malonylation, and glutarylation on lysine residues. In light of the SIRT5's roles in regulating mitochondrion function, we show here that SIRT5 deficiency leads to suppression of mitochondrial NADH oxidation and inhibition of ATP synthase activity. As a result, SIRT5 deficiency decreases mitochondrial ATP production, increases AMP/ATP ratio, and subsequently activates AMP-activated protein kinase (AMPK) in cultured cells and mouse hearts under energy stress conditions. Moreover, Sirt5 knockout attenuates transverse aortic constriction (TAC)-induced cardiac hypertrophy and cardiac dysfunction in mice, which is associated with decreased ATP level, increased AMP/ATP ratio and enhanced AMPK activation. Our study thus uncovers an important role of SIRT5 in regulating cellular energy metabolism and AMPK activation in response to energy stress.

## Introduction

Large-scale proteomic surveys of cellular proteins have shown that lysine can be posttranslationally modified by diverse acylations, including acetylation [1], succinylation [2], malonylation [3], glutarylation [4], crotonylation [5], propionylation [6], and butyrylation [6]. Among these lysine acylation modifications, acetylation is the best studied regulatory mechanism for multiple metabolic processes. Acetylation state of a given protein results from the balanced action of lysine acetyltransferases and deacetylases, which catalyze the addition and removal, respectively, of an acetyl group from a lysine residue. Like acetylation, other lysine acylation modifications can also regulate cellular metabolism pathways, such as succinylation, malonylation, and glutarylation [3, 4, 7]. Among these modifications, lysine succinylation is considered as a spontaneous, enzyme-independent modification, especially in the mitochondrial matrix [8–10]. Systematic profiling of the mammalian succinylome has identified 858 succinylation sites on 124 proteins and more than 75% of these succinylated proteins are localized in mitochondria [11]. Unique conditions of the mitochondrial matrix, such alkaline pH and concentrated succinyl-CoA support the non-enzymatic reaction of lysine residues by succinyl-CoA [8]. It is thus not surprising that succinylated proteins are enriched in the mitochondrion [9]. Whether there is a lysine succinyltransferase that catalyzes the forward reaction of lysine succinylation using succinyl-CoA as a substrate is a nascent area of investigation, but two recent studies identified lysine acetyltransferase 2A (KAT2A, also known as GCN5) as a potential succinyltransferase of histone H3 [12], and carnitine palmitoyltransferase 1A (CPT1A) as a succinyltransferase of enolase 1 [13].

So far, investigation on the regulation of succinylation, malonylation, and glutarylation and their biological relevance has mostly been focusing on the NAD^+^-dependent deacylase sirtuin 5 (SIRT5), which has been proposed to play an important role in the maintenance of metabolic health in cells [2, 4]. Numerous previous studies have reported the role of SIRT5 in regulating metabolic pathways, including fatty acid oxidation (FAO), urea cycle, ketogenesis, and so forth. For examples, SIRT5 desuccinylates and increases the activity of very longchain acyl-CoA dehydrogenase (VLCAD) to facilitate FAO flux [14]. SIRT5 also desuccinylates and increases the activity of enoyl-CoA hydratase α-subunit (ECHA) in FAO in Sirt5 KO mouse hearts [11]. Moreover, SIRT5 desuccinylates glutaminase (GLS) and suppresses the activity of GLS to reduce ammonia production in human breast cancer cells MDA-MB-231 and mouse myoblast cells C2C12 [15]. SIRT5 also desuccinylates and activates the activity of carbamoyl phosphate synthetase 1 (CPS1) to neutralize ammonia in Sirt5 KO mouse liver [2]. Furthermore, succinylation inhibits the rate-limiting ketogenic enzyme 3-hydroxy-3-methylglutaryl-CoA synthase 2 (HMGCS2) and thereby suppresses ketogenesis in Sirt5 KO mouse livers [16]. SIRT5-mediated succinylation has also been shown to regulate the enzyme activities of pyruvate kinase M2 (PKM2) and succinate dehydrogenase (SDH), but the results are contradictory in different experimental studies [7, 17–19]. Additionally, SIRT5 is vital for preserving normal respiratory capacity of mitochondrion in murine embryonic fibroblasts (MEFs), HT1080 human fibrosarcoma cells harboring IDH1 R132C mutation, and mouse cortices [20–22].

In this study, we conducted an unbiased metabolic profiling in SIRT5 KO HEK293T cells, and discovered that SIRT5 deficiency significantly alters intermediates of multiple metabolic pathways. Notably, SIRT5 KO increases mitochondrial NADH, suppresses ATP production, and increases AMP/ATP ratio, leading to enhanced AMPK activation in both cultured cells and mouse hearts under energy stress conditions.

## Materials and methods

### Antibodies

Antibodies specific to β-ACTIN (GeneScript, mouse monoclonal antibody, A00702, 1:10000), SIRT5 (Cell Signaling Technology, rabbit monoclonal antibody, 8782, 1:1000), AMPKα (Cell Signaling Technology, rabbit polyclonal antibody, 2532, 1:1000), phospho-AMPKα (Thr172) (Cell Signaling Technology, rabbit monoclonal antibody, 2535, 1:1000), ACC (Cell Signaling Technology, rabbit monoclonal antibody, 3676, 1:1000), phospho-ACC (Ser79) (Cell Signaling Technology, rabbit polyclonal antibody, 3661, 1:1000), 4EBP1 (Cell Signaling Technology, rabbit polyclonal antibody, 9452, 1:1000), phospho-4EBP1 (Thr70) (Cell Signaling Technology, rabbit polyclonal antibody, 9455, 1:1000), ATP synthase (Abcam, mouse monoclonal antibody, ab109867, 10 μg/ml), ATP5B (Proteintech, rabbit polyclonal antibody, 17247-1-AP, 1:1000), SDHA (Abcam, rabbit polyclonal antibody, ab66484, 1:1000), pan-Succinyl-lysine (PTM Biolabs, mouse monoclonal antibody, PTM-419, 1:1000), pan-Malonyl-lysine (PTM Biolabs, rabbit polyclonal antibody, PTM-901, 1:1000), pan-Glutaryl-lysine (PTM Biolabs, rabbit polyclonal antibody, PTM-1151, 1:1000), and pan-Acetyl-Lysine (Cell Signaling Technology, mouse monoclonal antibody, 9681, 1:1000) were purchased commercially. Secondary antibodies for monoclonal mouse anti-rabbit IgG light chain and polyclonal goat anti-mouse IgG light chain (Jackson ImmunoResearch, 211-032-171 and 115-035-174, respectively, both 1:3000) were also purchased commercially.

### Plasmid construction

The cDNA encoding full-length human SIRT5 and ATP5B were cloned into HA-tagged pcDNA-3.1-B vector and Flag-tagged peGFP-C3 vector, respectively. HA-SIRT5^H158Y^ point mutant was generated from HA-SIRT5 by using the AccuPrime *Pfx* DNA Polymerase (Invitrogen, 12344024).

### Cell culture, transfection, and treatment

HEK293T cells (Obtained from ATCC in November 2015, ATCC CRL-11268) were cultured in Dulbecco’s Modified Eagle’s Medium (DMEM, Gibco) supplemented with 5% fetal bovine serum (Gibco), 100 units/ml penicillin and 100 μg/ml streptomycin, in 5% CO_2_ atmosphere at 37°C. Cell transfection was carried out by polyethyleneimine (PEI, Sigma-Aldrich). For glucose and glutamine starvation, HEK293T cells were washed with PBS, and incubated with DMEM (No glucose, no glutamine, Gibco) plus 0.1 mM glucose (Sigma-Aldrich) and 1 mM glutamine (Gibco) for the indicated time.

### Animals

Sirt5 KO mice (129-strain background) were obtained from the Jackson Laboratory, and were backcrossed for eight generations onto the C57BL/6J background. The animal protocols were approved by the Institutional Animal Care and Use Committee (IACUC) and Ethics committee of Fudan University (Shanghai, China). Animals were given unrestricted access to a standard diet and tap water, and animal experiments were performed at Fudan Animal Center and Shanghai Biomodel Organism Co., Ltd in accordance with the animal ware fare guidelines under the Division of Laboratory Animal Medicine regulations of Fudan University. All invasive animal procedures were performed under anesthesia (1.5%-2% isoflurane). Humane endpoints were defined as a rapid loss of 15%-20% of body weight in one day, or inability to ambulate or rise for food and water. If reaching these endpoints, animals were euthanized by using CO2 gas inhalation. All efforts were made to minimize suffering.

### Generation of SIRT5 knockout cells by using CRISPR/Cas9 genome editing

To generate SIRT5 KO HEK293T cells, the following three guide sequences targeting the human *SIRT5* gene were used: 5’-AGTGGTGTTCCGACCTTCAG-3’, 5’-GTTCCGACCTTCAGAG GAGC-3’, and 5’-AAGCACATAGTCATCATCTC-3’. The detailed protocol is described elsewhere [23]. Briefly, all three guide RNA sequences were respectively cloned into the plasmid px459 (Addgene, 48319). The plasmids were transfected into HEK293T cells. For SIRT5 knockout cell pool, after transfection for 24 hours, puromycin was added into the cells for selection for 5 days, and then the remaining living cells were used to perform experiments. For SIRT5 knockout monoclones, transfected cells were selected by puromycin for 3 days. Then cells were sorted into 96-well plates with only one cell in each well. Clones were screened by western blot with the SIRT5-specific antibody, and gene deletion was verified by Sanger sequencing of genomic DNA. Two independent clones with *SIRT5* deletion were used for further experiments.

### Generation of *SIRT5* knockdown cells by retroviral infection

To generate stable HEK293T cells with *SIRT5* knockdown, short-hairpin RNA (shRNA) oligos targeting *SIRT5* were cloned into the retroviral pMKO.1 vector according to standard cloning protocol. The two shRNA sequences targeting *SIRT5* are 5’-GCCCTTGAACATTTCCCAATG-3’ and 5’-GCATTAGAACTACAGACAAC-3’ [20]. And retrovirus was produced using a two-plasmid packaging system as previously described [24]. Briefly, the shRNA plasmids were co-transfected with two packing plasmids, vsvg and gag, into HEK293T cells. Retroviral supernatant was harvested at 36 hours after transfection and was used to infect the target cells. Polybrene was added to increase the infection efficiency. The infected cells were then treated and selected with 1 μg/ml puromycin for 1 week.

### Immunoprecipitation and western blotting

For whole cell lysate, cells were lysed in SDS sample buffer and denatured by heating on 99°C for 15 min and then subjected to western blot; For mouse heart lysate, mouse hearts were homogenized in ice-cold 0.5% NP-40 buffer containing 50 mM Tris-HCl (pH 7.5), 150 mM NaCl, 0.5% NP-40, 1 mM Na_3_VO_4_ and protease inhibitor cocktail (Biotool) with the Tissuelyser-24 (Shanghai JingXin). Heart homogenates were lysed with rotation at 4°C for 30 min and centrifugated at 13,000 rpm for 15 min at 4°C. The supernatant was collected, lysed in SDS sample buffer and denatured by heating on 99°C for 15 min, followed by direct western blot analysis; For ATP synthase immunoprecipitation, mouse hearts were lysed as described above. The heart supernatants or mitochondria (as described below) were incubated with an anti-ATP synthase antibody (Abcam) for 3 hours at 4°C, and then protein A beads (Repligen) were added into the supernatant for another 2 hours at 4°C, washed 3 times with ice-cold NP-40 buffer and analyzed by SDS-PAGE and immunoblotting according to the standard methods.

### Measurement of cytosolic and mitochondrial NADH levels

FrexH and Frex biosensors were designed to detect subcellular NADH in different cell compartments as described previously [25]. These two plasmids were tagged with organelle-specific signal peptides to direct them to express recombinant FrexH and Frex proteins in the cytosol and mitochondria, respectively [25]. At 30 hours after transfection, HEK293T cells were harvested with trypsin digestion and were washed and resuspended in PBS. Fluorescence excited at 420 nm (FrexH) and 485 nm (Frex) were measured by a BioTek Synergy Microplate Reader with emission filter 528 nm. Fluorescence values were background-calibrated by subtracting the fluorescence intensity of HEK293T cells not expressing FrexH or Frex.

### Isolation of mouse heart mitochondria

Mouse heart mitochondria were isolated as described previously [26]. Briefly, mouse heart tissue was cut into small pieces, added in 2 volumes of Buffer A (250 mM sucrose, 10 mM HEPES, 10 mM Tris-HCl, 1 mM EGTA, 1 mM Na_3_VO_4_, TSA, NAM and protease inhibitor cocktail, pH 7.4). Pieces were homogenized with a dounce homogenizer with up and down strokes for 25 times. The homogenate was centrifuged at 800 g for 8 min at 4°C to pellet the debris, and the supernatant was then subjected to centrifugation at 4000 g for 30 min at 4°C to pellet the mitochondria. Wash the pellet with Buffer B (250 mM sucrose, 20 mM HEPES, 10 mM Tris-HCl, 1 mM Na_3_VO_4_, TSA, NAM and protease inhibitor cocktail, pH 7.4), gently beating upon the pellet.

### Solubilization of mouse heart mitochondria

Mitochondria isolated above were diluted to 5.5 mg/ml in 50 mM Tris-HCl, 1% (v/v) protease inhibitor cocktail (Biotool), pH 7.5 and solubilized by adding 1/10 volume of 10% (w/v) n-dodecyl-D maltopyranoside (J&K Scientific) [27]. The final protein concentration was 5 mg/ml. The samples were incubated on ice for 30 min and centrifuged at maximum speed (~16,000 rpm) for 20 min at 4°C. Discard the pellet. The supernatant was used to measure the citrate synthase activity and ATP synthase activity [27].

### LC-MS/MS for metabolic profiling

Metabolite extraction from cells or mouse heart mitochondria was conducted as described above. LC-MS/MS-based metabolic profiling was performed as described elsewhere [28]. Briefly, the lyophilized samples were resuspended in 100 μL of reconstitution fluid (ACN: H2O = 75:25, v/v, containing 10 mM ammonium acetate, 0.01% phosphoric acid), mixed well and centrifuged for 5 min at 14000 rpm. Aliquot 90 μL of supernatant was then subjected to LC-MS/MS (Waters ACQUITY UPLC I-Class system coupled with Xevo TQ-XS mass spectrometry). Separation of metabolites was achieved in Waters ACQUITY UPLC BEH Amide column (2.1 mm × 100 mm × 1.7 μm) or ACQUITY UPLC HSS T3 column (2.1 mm × 50 mm × 1.8 μm). The elution gradient was set as follows: 99% A (0.0–0.1 min), 99–30% A (0.1–6 min), 30–99% (6.0–6.5 min), 99–99% A (6.5–10 min), or 100% A (0.0–0.2 min), 100–97% A (0.2–1.0 min), 97–5% (1.0–1.5 min), 5–5% A (1.5–2.0 min), 5–100% A (2.0–2.01 min), 100–100% A (2.01–2.5 min). The column temperature was set to be 45°C and the flow rate was 0.4 ml/min. The data were acquired in both ESI positive and negative modes. The Capillary was 2.5 kV for positive mode and 1.0 kV for negative mode and the Cone was 20 V. The Desolvation Temp and Source Temp were set to 600°C and 150°C, respectively. The mass spectrometer was optimized and set up in selected reaction monitoring (SRM) scan mode for monitoring the [M-H] of ATP (m/z 507.96→136.03), ADP (m/z 427.98→136.1) and AMP (m/z 346.09→78.98). Principal component analysis and orthogonal projections to latent structure-discriminant analysis were conducted, and SIMCA tool (version 14.1, trial, Umetrics, Sweden) was used for data analysis.

### Absolute quantification of ATP

ATP contents were measured in the hearts (20 mg) of female Sirt5 WT and KO mice after overnight fasting by using the ATP Colorimetric/Fluorometric Assay Kit (BioVision).

### Citrate synthase activity assay

Citrate synthase activity was measured as described previously [29]. The activity was determined as the reduction of 5, 5-dithiobis-2-nitrobenzoic acid (DTNB), monitored at 412 nm in the reaction buffer (100 mM Tris-HCl, 0.2 mM acetyl-CoA, 0.1 mM DTNB, 0.1% Triton X-100 and 0.5 mM oxaloacetate, pH 8.0).

### ATP synthase activity assay

ATP synthase activity was measured as described previously [29]. The reaction buffer contained 50 mM Tris-HCl (pH 8.0), 5 mg/ml BSA, 20 mM MgCl_2_, 25 mM KCl, 15 μM CCCP, 5 μM Antimycin A, 2.5 mM PEP, 2.5 mM ATP, 30 U/ml PK, 30 U/ml LDH and 0.5 mM NADH, with the rate being monitored by the oxidation of NADH at 340 nm. The oligomycin-sensitive activity of ATP synthase was determined with 4 μM oligomycin.

### Transverse aortic constriction (TAC) surgery

Male Sirt5 KO mice and WT littermates (10 weeks of age) were used for the experiments. Pressure overload was created by TAC as previously described [30]. Briefly, mice were anesthetized by inhalation of 2% isoflurane, endotracheally intubated and ventilated (Type 7025; Harvard Apparatus, March-Hugstetten, Germany). Left-sided parasternal thoracotomy was performed in the second intercostal space. After isolation of the aortic arch, it was tied steadily against a 27-gauge needle with a 6–0 silk ligature between the origins of innominate artery and left common carotid artery, and then promptly removed to yield a constriction. Finally, the chest wall was closed with a 4–0 silk suture, and meloxicam (0.13 mg each) was administered subcutaneously for analgesia. Correspondingly, the sham-operated mice underwent the similar operation without aortic banding. All operations were performed by a single operator with skilled experience in handling mouse cardiac surgery. Mice were monitored for 5 consecutive weeks.

### Echocardiographic measurements

High-frequency echocardiography was performed using a Vevo770 system (VisualSonics Inc., Toronto, Canada) with a transducer of 30-MHz center frequency. During the experiment, the mice were anesthetized with isoflurane (3%-4% for induction and 1.5% for maintenance). The structures and hemodynamics of left ventricle and aortic arch were measured as described previously [31]. The assessment parameters included peak flow velocity at aortic banding site, LVAWTd, LVAWTs, LVPWTd, LVPWTs, LV mass, LVIDd, LVIDs, LVEDV, LVESV, LVEF and LVFS. Heart rate maintained around 500 bpm, and the intercept angle between Doppler beam line and flow direction was controlled within 30 degrees. When echocardiography was performed on the mice, a parasternal long-axis B-mode image was acquired with appropriate positioning of the scanhead so that the maximum left ventricular length, the outflow tract, and the papillary muscle could be identified. From this view, an M-mode sample line was positioned at the place where the LV diameter was the largest (usually near the root of the papillary muscle), and perpendicular to the anterior and posterior walls of the LV. M-mode image loops were obtained for measurement of wall thickness and chamber dimensions. LVEF and LVFS images are calculated automatically by the ultrasound system according to the values of inner diameters of LV. The papillary muscle can be easily shown in systole, especially for TAC mice. Such image recordings and measurements can be decently found in previous literature by us and others [32, 33]. Echocardiographic assessment showed good intraobserver and interobserver agreement, as reported in previous studies [31, 34]. All the echocardiography was performed by a single operator blindly.

### RNA isolation and quantitative real-time PCR

Total RNA was extracted from mouse hearts by TRIzol reagent according to the manufacturer instructions (Invitrogen). RNA was reverse-transcribed to cDNA with oligo (dT) primers and preceded to real-time PCR with gene-specific primers using SYBR Premix Ex Taq (TaKaRa). β-Actin was used as the endogenous control. Primers used in the study were as follows (5'-3'):

*β-Actin-*Forward: CATTGCTGACAGGATGCAGAAGG;
*β-Actin*-Reverse: TGCTGGAAGGTGGACAGTGAGG;
*Sirt5-*Forward: CGCTGGAGGTTACTGGAGAA;
*Sirt5*-Reverse: GCGATGCAACTCGTCAATGT;
*Anp*-Forward: TTTCAAGAACCTGCTAGACCACC;
*Anp*-Reverse: ATCTATCGGAGGGGTCCCA;
*Bnp-*Forward: CTGAAGGTGCTGTCCCAGAT;
*Bnp*-Reverse: CCTTGGTCCTTCAAGAGCTG;
*Collagen 1a1*-Forward: GAGCGGAGAGTACTGGATCG;
*Collagen 1a1-*Reverse: GTTCGGGCTGATGTACCAGT;
*Collagen3a1-*Forward: GGAATGGAGCAAGACAGTCTTTG;
*Collagen3a1-*Reverse: TGCGATATCTATGATGGGTAGTCTCA;

### Statistics

Most statistical analyses were performed with a two-tailed unpaired Student's t-test, and TAC-surgery related data were analyzed with one-way ANOVA. All data shown represent the results obtained from triplicated or quadruplicated independent experiments with standard deviations of the mean (mean ± S.D.) or standard errors of the mean (mean ± SEM) (Table 1). The values of p<0.05 were considered statistically significant.

**Table 1:** Echocardiographic and hemodynamic measures in the Sham and TAC groups.

	WT-Sham	Sirt5 KO-Sham	WT-TAC	Sirt5 KO-TAC
	n = 6	n = 7	n = 9	n = 10
**Heart Rate (bpm)**	547.5 ± 11.08	523 ± 21.85	516.11 ± 10.92	504.7 ± 16.29
**Peak flow velocity (mm/s)**	790.19 ± 50.07	845.86 ± 33.43	3414.65 ± 5.96^###^	3386.51 ± 29.80^+++^
**LVAWTd (mm)**	0.92 ± 0.05	0.98 ± 0.05	1.45 ± 0.06^###^	1.34 ± 0.05^+++^
**LVAWTs (mm)**	1.33 ± 0.05	1.44 ± 0.05	1.89 ± 0.05^###^	1.80 ± 0.06^+++^
**LVPWTd (mm)**	0.8 ± 0.06	0.83 ± 0.06	1.36 ± 0.06^###^	1.35 ± 0.05^+++^
**LVPWTs (mm)**	1.28 ± 0.06	1.19 ± 0.11	1.73 ± 0.09^##^	1.53 ± 0.06^+^
**LVIDd (mm)**	3.96 ± 0.06	4.03 ± 0.05	4.22 ± 0.10	3.92 ± 0.06*
**LVIDs (mm)**	2.77 ± 0.09	2.86 ± 0.06	3.26 ± 0.15^#^	2.73 ± 0.10**
**LVEDV (μl)**	68.71 ± 2.44	71.46 ± 2.03	80.28 ± 4.66	67.10 ± 2.55*
**LVESV (μl)**	28.92 ± 2.34	31.28 ± 1.66	44.08 ± 5.27^#^	28.28 ± 2.55*
**Stroke Volume (μl)**	39.79 ± 2.40	40.68 ± 1.24	36.21 ± 2.30	38.82 ± 0.98
**LVEF (%)**	57.92 ± 2.95	56.33 ± 1.59	46.07 ± 3.45^#^	58.52 ± 2.44*
**LVFS (%)**	30.27 ± 2.02	29.11 ± 1.06	23.01 ± 2.01^#^	30.69 ± 1.67*
**LV Mass Corrected (mg)**	101.30 ± 7.63	108.40 ± 5.33	218.69 ± 13.59^###^	165.99 ± 9.11***^/^^+++^

LVAWTd = left ventricular diastolic anterior wall thickness; LVAWTs = left ventricular systolic anterior wall thickness; LVPWTd = left ventricular diastolic posterior wall thickness; LVPWTs = left ventricular systolic posterior wall thickness; LVIDd = left ventricular internal diastolic diameter; LVIDs = left ventricular internal systolic diameter; LVEDV = left ventricular end-diastolic volume; LVESV = left ventricular end-systolic volume; LVEF = left ventricular ejection fraction; LVFS = left ventricular shortening fraction. All data are shown as mean ± S.E.M.

#denotes the P < 0.05

##denotes the P < 0.01 and

###denotes the P < 0.001 for the comparisons between WT-Sham and WT-TAC groups.

+denotes the P < 0.05 and

+++denotes the P < 0.001 for the comparisons between Sirt5 KO-Sham and Sirt5 KO-TAC groups.

*denotes the P < 0.05

**denotes the P < 0.01 and

***denotes the P < 0.001 for the comparisons between WT-TAC and Sirt5 KO-TAC groups.

## Results

### SIRT5 KO leads to increased AMP/ATP ratio and enhanced AMPK activation in HEK293T cells

**Fig 1:**
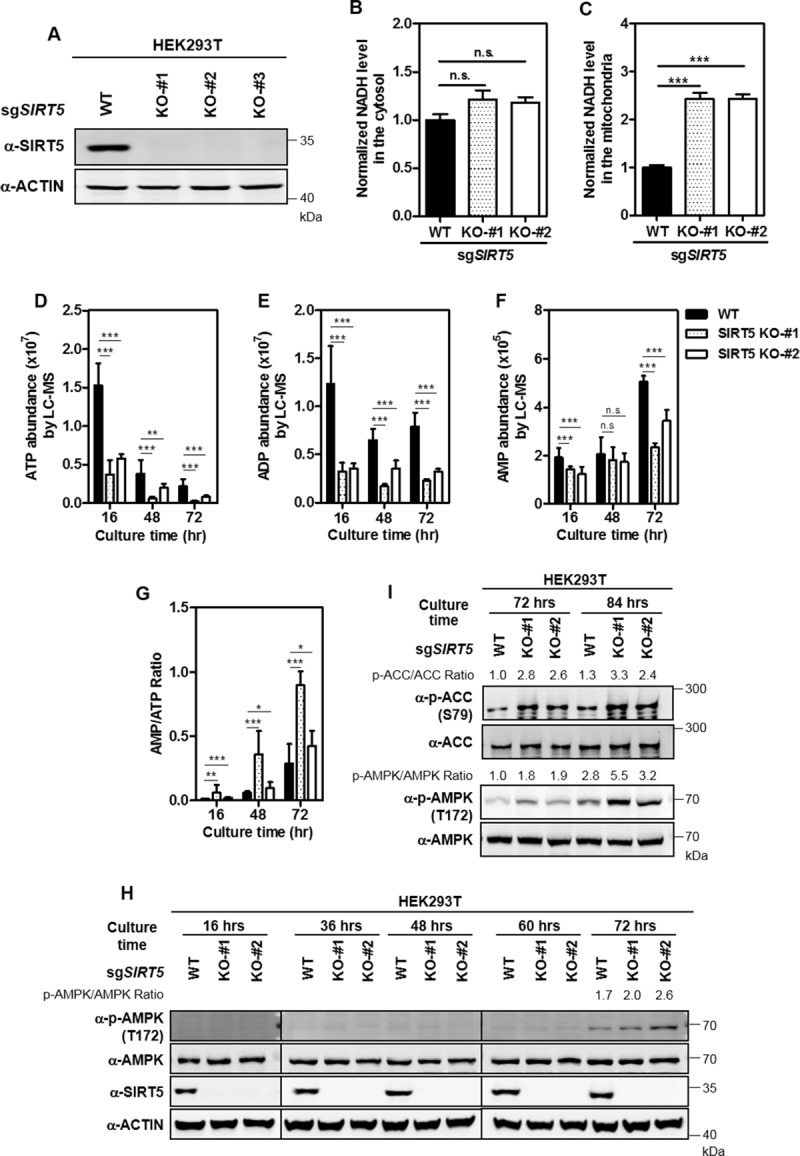
SIRT5 KO leads to increased AMP/ATP ratio and AMPK activation in HEK293T cells. (A) Verification of SIRT5 KO in HEK293T cells by immunoblotting with the indicated antibodies. (B-C) Cytosolic and mitochondrial NADH levels were measured by genetically encoded fluorescent protein biosensors as described in ‘Materials and Methods’. (D-G) The AMP/ATP ratio is significantly increased in SIRT5 KO HEK293T cells. 1×10^6^ cells were seeded into each well of six-well plates. After culture for 16, 48, and 72 hours, the cells were subjected to LC-MS/MS for metabolic profiling as described in ‘Materials and Methods’. Relative levels of ATP (D), ADP (E), AMP (F) and AMP/ATP ratio (G) were quantified. n = 3 or 4 for each cell line. Data are shown as mean ± SD of at least 3 independent experiments, two-tailed unpaired Student's t-test. *denotes the P < 0.05, **denotes the P < 0.01, and ***denotes the P < 0.001 for the indicated comparisons. n.s. = not significant. (H-I) AMPK activation in SIRT5 KO HEK293T cells. Cells were collected at the indicated culture periods, and AMPK T172 phosphorylation (H-I) and ACC S79 phosphorylation (I) were detected by immunoblotting using the indicated antibodies. WB bands for detecting the same proteins at 36, 48, 60, and 72 hours in (H) are from the same gels.

To decipher the role of SIRT5 in regulating cellular metabolism, we generated SIRT5 KO HEK293T cells by using CRISPR/Cas9-mediated genome editing technique (S1 Fig and Fig 1A). We found that SIRT5 KO did not change cytosolic NADH level (S2 Fig and Fig 1B), but significantly increased the level of mitochondrial NADH by as much as 2.4-fold (P<0.001) (S2 Fig and Fig 1C). It is acknowledged that NADH acts as a coenzyme in numerous central housekeeping redox reactions, and is oxidized through the respiratory chain and oxidative phosphorylation for electron transport and ATP synthesis [35–38]. The observed effect of SIRT5 KO on increasing mitochondrial NADH suggests that NADH oxidation is hindered by SIRT5 loss in the mitochondrion.

Next, we conducted metabolic profiling in SIRT5 wild-type (WT) and KO cells after different time periods of plating by using liquid chromatography-tandem mass spectrometry analysis (LC-MS/MS). Principal component analysis (PCA) revealed that SIRT5 KO and WT cells were separated into two clusters especially at 72 hours after plating (S3 Fig). As shown in the loading plot, p1 (i.e. the X-axis) is for distinguishing 16, 48, and 72 hours of plating, while p2 (i.e. the Y-axis) is for distinguishing WT and KO cells, and metabolites in the upper right panel of the plot altered significantly, including ATP and ADP in the adenine nucleotide biosynthesis pathway, glutamate and glutamine in the anaplerotic pathway, malate, fumarate, alpha-KG, and citrate in the TCA cycle, threonine, alanine, and glycine in the acetyl-CoA biosynthesis pathway, and methionine in the succinyl-CoA biosynthesis pathway (S4 Fig). Likewise, orthogonal projections to latent structure-discriminant analysis (OPLS-DA) showed that SIRT5 KO and WT cells were separated into two clusters at 16 and 72 hours after plating, and that ATP level was greatly decreased in SIRT5 KO cells compared to WT cells at the above mentioned time points after plating (S5–S8 Figs). Quantitative results showed that relative ATP level was decreased by as much as 75.4% (P<0.001) in SIRT5 KO cells compared to WT cells at 16 hours after plating or prolonged culture periods (Fig 1D). Relative levels of ADP and AMP were also reduced in SIRT5 KO cells compared with WT cells (Fig 1E and 1F). As a result, the AMP/ATP ratio was consistently and significantly increased in SIRT5 KO cells compared to WT cells at 16, 48, and 72 hours after plating (Fig 1G).

A high AMP/ATP ratio can promote activation of AMPK, a fundamental component of cellular response to energy stress [39]. AMPK is composed of a catalytic alpha subunit and two regulatory beta and gamma subunits [39]. The γ subunit includes four particular cystathionine beta synthase (CBS) domains that can bind adenine nucleotides, giving AMPK its ability to sense the AMP/ATP ratio [40]. The observed increase in AMP/ATP ratio encouraged us to examine AMPK activation status in SIRT5 KO cells by checking phosphorylation at threonine 172 in the catalytic domain of AMPK [39]. In accord with higher AMP/ATP ratio, AMPK T172 phosphorylation was increased in SIRT5 KO cells compared to control cells at 72 hours after plating when cells reached full confluency, a condition mimicking energy stress (Fig 1H). Phosphorylation of the AMPK substrate Acetyl-CoA Carboxylase (ACC) on serine 79 was increased in SIRT5 KO cells compared with WT cells at 72 or 84 hours after plating (Fig 1I). Furthermore, we found that putting-back wild-type or catalytic dead mutant (SIRT5^H158Y^) failed to attenuate the increased phosphorylation of AMPK in SIRT5 KO HEK293T cells (S9 Fig).

In addition, we also noticed that SIRT5 KO-#2 showed a much weaker phenotype in increasing AMP/ATP ratio than SIRT5 KO-#1 (Fig 1G). For verification, we generated SIRT5 knockout cell pool (by sgRNA) and stable *SIRT5* knockdown (by two different shRNAs against *SIRT5*) in HEK293T cells. In agreement with the findings above, ATP reduction, higher AMP/ATP ratio, and AMPK activation were observed (S10A–S10F Fig), re-affirming the potential role of SIRT5 in controlling cellular ATP production and AMPK activity.

Collectively, these results indicate that SIRT5 plays an important role in controlling mitochondrial NADH oxidation, and cellular energy metabolism. SIRT5 deficiency leads to reduced ATP, higher AMP/ATP ratio and subsequent AMPK activation in cultured cells under energy stress conditions.

### Sirt5 KO increases AMP/ATP ratio and activates AMPK in mouse hearts under fasting condition

**Fig 2:**
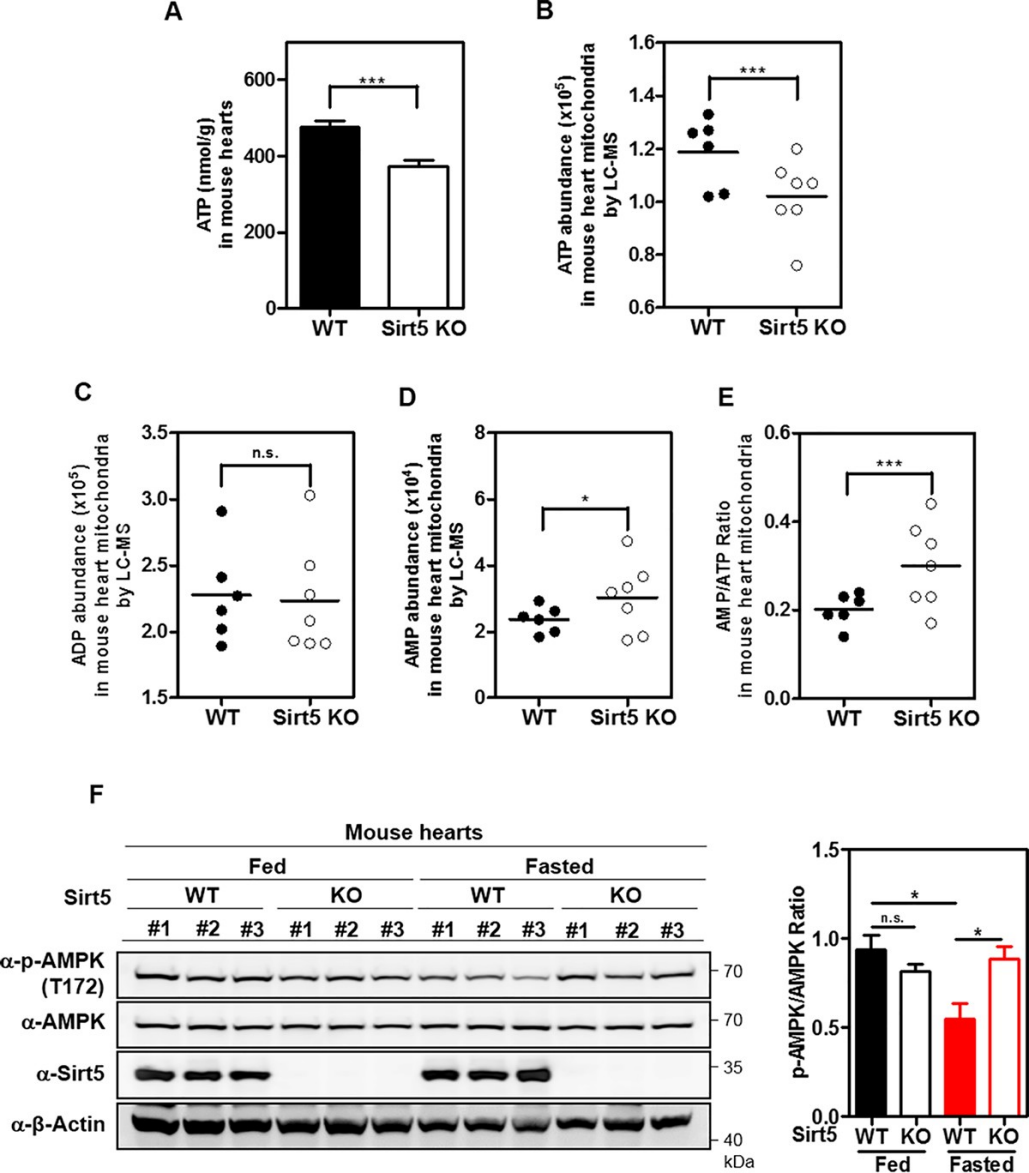
Sirt5 KO leads to increased AMP/ATP ratio and AMPK activation in mouse hearts. (A) Sirt5 deficiency suppresses ATP production in hearts of fasted mice. Female Sirt5 KO mice (n = 3) and WT littermates (n = 3) (4 weeks old, 16–19 g) were fasted overnight. Upon sacrifice, mouse hearts were harvested and then subjected to determination of ATP as described in ‘Materials and Methods’. (B-E) Sirt5 deficiency suppresses mitochondrial ATP production in mouse heart. Sirt5 KO mice (n = 7) and sex-matched WT control mice (n = 6) (16–28 weeks old) were fasted overnight. Upon sacrifice, mouse hearts were harvested for isolation of cardiac mitochondrion as described in ‘Materials and Methods’. Relative levels of ATP (B), ADP (C), and AMP (D) were measured by LC-MS/MS analysis, and the AMP/ATP ratio was calculated (E). (F) Sirt5 deficiency enhances AMPK activation in hearts of fasted mice. Sirt5 KO mice (n = 6) and sex-matched WT control mice (n = 6) (12 weeks old) were fed normally or fasted overnight. Upon sacrifice, mouse hearts were harvested, and AMPK T172 phosphorylation was detected by immunoblotting using the indicated antibodies. Data are shown as mean ± SD of at least 3 independent experiments, two-tailed unpaired Student's t-test. *denotes the P < 0.05 and ***denotes the P < 0.001 for the indicated comparisons. n.s. = not significant.

Mitochondria occupy approximately 30% of cardiomyocyte space, and produce more than 90% of energy for heart muscle [41, 42]. We found after overnight fasting that ATP production was significantly decreased (by 21.8%, P<0.001) in the heart of Sirt5 KO mice (n = 3) compared with WT littermates (n = 3) (Fig 2A). In cardiac mitochondrion samples, ATP was decreased by 14% (P<0.001) in the heart of Sirt5 KO mice (n = 7) compared with that of WT mice (n = 6), while relative AMP was increased by 28% (P<0.05) (Fig 2B and 2D). Relative ADP was unchanged in heart mitochondria of Sirt5 KO mice compared with that of WT mice (Fig 2C). As a result, the AMP/ATP ratio was significantly increased by 48.8% (P<0.001) in heart mitochondria of Sirt5 KO mice (Fig 2E).

Next, we examined the status of AMPK activation in hearts of Sirt5 KO and WT mice. Under normal fed conditions, AMPK T172 phosphorylation in the heart was comparable between Sirt5 KO (n = 3) and WT (n = 3) mice. However, AMPK T172 phosphorylation was significantly increased by 1.6-fold (P<0.05) in the heart of Sirt5 KO mice compared to that of WT mice under fasting conditions (Fig 2F).

Together, these findings provide *in vivo* data supporting that Sirt5 deficiency impairs mitochondrial ATP production and enhances AMPK activation in mouse hearts under fasting condition.

### Sirt5 KO leads to increased lysine succinylation and decreased ATP synthase activity

**Fig 3:**
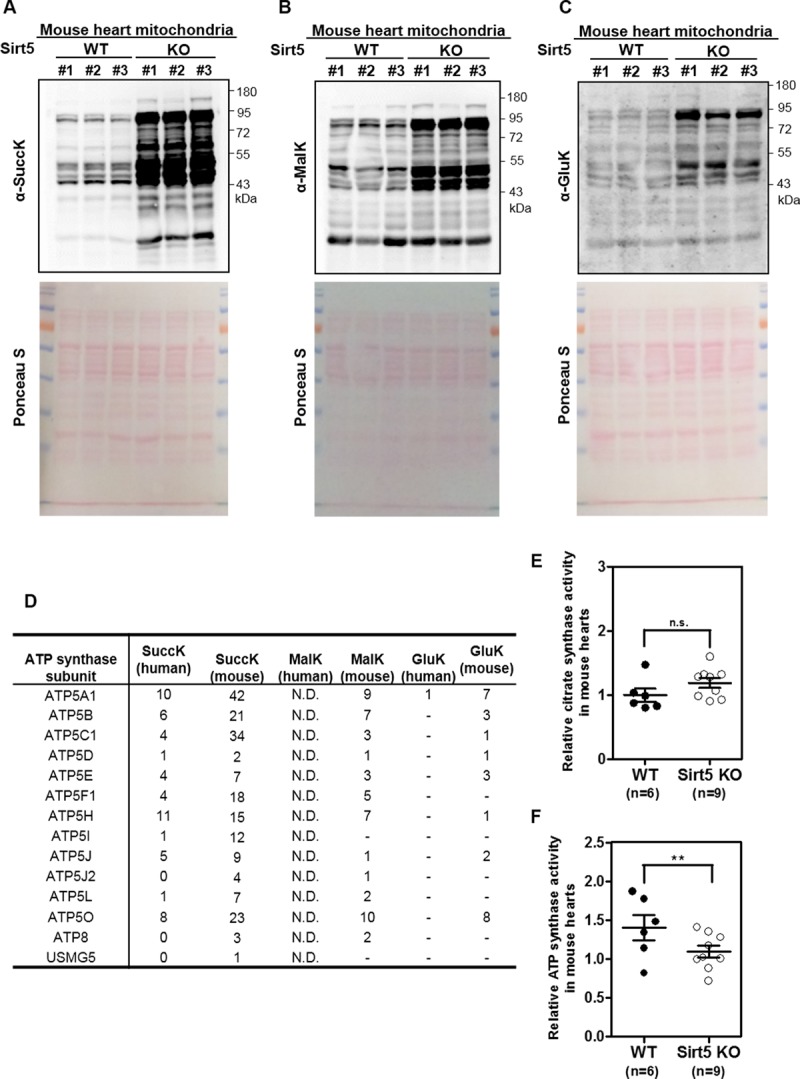
Sirt5 KO induces increased lysine succinylation and decreased ATP synthase activity in mouse heart mitochondria. (A-C) Lysine succinylation, malonylation and glutarylation are dramatically increased in mitochondria of Sirt5 KO mouse heart. Male Sirt5 KO mice (n = 3) and WT control mice (n = 3) (16–28 weeks old) were fasted overnight. Upon sacrifice, mouse hearts were harvested for isolation of cardiac mitochondria. Immunoblotting was performed using the anti-sucinyllysine antibody (A), anti-malonyllysine antibody (B), and anti-glutaryllysine antibody (C). Total protein loading was stained with Ponceau S. (D) Summary of previous proteomic studies identifying ATP synthase subunits as succinylation/malonylation/glutarylation substrates regulated by SIRT5. The numbers of lysine sites which are regulated by succinylation, malonylation, glutarylation and SIRT5 were listed. N.D. = not determined. (E-F) Sirt5 deficiency inhibits ATP synthase activity in hearts of fasted mice. Sirt5 KO mice (n = 9) and sex-matched WT control mice (n = 6) (16–28 weeks old) were fasted overnight. Upon sacrifice, mouse hearts were harvested for isolation of cardiac mitochondrion, and were then subjected to citrate synthase activity (E) and ATP synthase activity (F) assays as described in ‘Materials and Methods’. Data are shown as mean ± SD of at least 3 independent experiments, two-tailed unpaired Student's t-test. **denotes the P < 0.01 for the indicated comparison. n.s. = not significant.

As noted in the introduction, SIRT5 is a robust desuccinylase, demalonylase and deglutarylase [2, 4, 7]. As expected, we found that lysine succinylation, malonylation, and glutarylation were remarkably increased in cardiac mitochondria of Sirt5 KO mice compared to that of WT mice, while lysine acetylation was substantially not changed (Fig 3A–3C and S11 Fig).

The mammalian succinylome studies have identified oxidative phosphorylation as one of the top scoring pathways in the heart mitochondria of Sirt5 WT and KO mice [43, 44]. To investigate the mechanism of Sirt5 in regulating mitochondrial ATP production in mouse hearts, we evaluated the enzymatic activity of ATP synthase, which is directly responsible for mitochondrial ATP production in the oxidative phosphorylation [45]. As summarized in Fig 3D and S1 Table, most of ATP synthase subunits were identified as potential substrates of SIRT5-regulated lysine succinylation in both mice and human, while lysine malonylation and glutarylation of ATP synthase subunits were much less identified. Immunoprecipitation (IP) assay using an antibody against ATP synthase revealed that lysine succinylation of Atp5b, a member of ATP synthase catalytic core, was increased in heart mitochondria of Sirt5 KO mice compared to WT mice (S12 Fig). However, neither lysine malonylation nor glutarylation of Atp5b could be detected (S12 Fig). To test the enzyme activity of ATP synthase, Oligomycin-sensitivity of this enzyme was assessed in mouse heart mitochondrial protein extracts (S13A and S13B Fig). The enzyme activity of citrate synthase, representing mitochondrial mass as an internal control [29, 46, 47], did not differ between heart mitochondria of Sirt5 KO and WT fasted mice (Fig 3E). After normalized with citrate synthase activity, we found under fasting conditions that the activity of ATP synthase was significantly decreased by 22% (P<0.01) in heart mitochondrial lysates from Sirt5 KO mice (n = 9) compared to that of WT mice (n = 6) (Fig 3F). Meanwhile, we determined the protein level of Sdha (S14A Fig), and used this mitochondrial protein to normalize the activity of ATP synthase. Again, ATP synthase activity was significantly reduced by 19.3% (P<0.01) in heart mitochondrial lysates from Sirt5 KO mice (n = 9) compared to that of WT mice (n = 6) (S14B Fig).

Taken together, these results suggest that Sirt5 deficiency leads to inhibition of ATP synthase activity in heart mitochondria of fasted mice, which is most likely to be associated with increased lysine succinylation.

### Sirt5 KO prevents left ventricular dilation and cardiac dysfunction in transverse aortic constriction (TAC) mice

Numerous previous studies have suggested that AMPK prevents cardiac hypertrophy and cardiac dysfunction by inhibiting protein synthesis [48, 49]. Our earlier observation that Sirt5 deficiency enhances AMPK activation inspired us to assess the cardiac function in Sirt5 KO and WT mice after TAC, a well-characterized animal model for pressure overload-induced left ventricular hypertrophy [30]. Male Sirt5 KO mice and WT littermates (10 weeks of age) underwent TAC surgery and were then monitored for 5 consecutive weeks.

**Fig 4:**
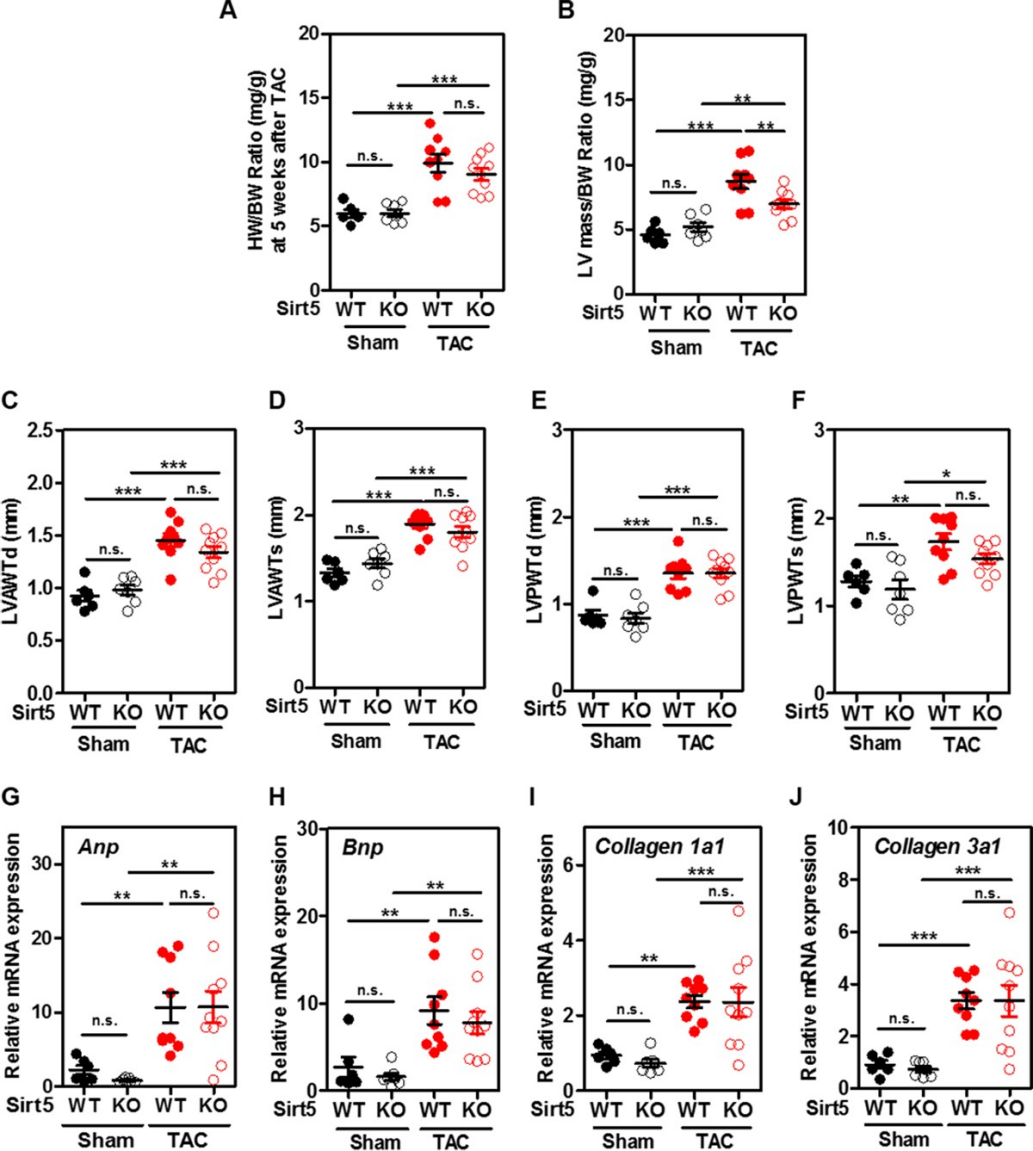
Sirt5 KO prevents left ventricular dilation in TAC mice. (A) The HW/BW ratio between male Sirt5 KO mice and WT littermates at 5 weeks after TAC surgery (n = 6–10 per group). HW, heart weight; BW, body weight. (B-F) Left ventricular hypertrophic parameters between male Sirt5 KO mice and WT littermates at 5 weeks after TAC surgery (n = 6–10 per group), including left ventricular mass/body weight (LV mass/BW) ratio (B), left ventricular diastolic anterior wall thickness (LVAWTd) (C), left ventricular systolic anterior wall thickness (LVAWTs) (D), left ventricular diastolic posterior wall thickness (LVPWTd) (E), and left ventricular systolic posterior wall thickness (LVPWTs) (F). (G-J) Changes in other hallmarks of hypertrophic cardiomyopathy and cardiac fibrotic markers between male Sirt5 KO mice and WT littermates at 5 weeks after TAC surgery (n = 6–10 per group). Hypertrophic markers, *Anp* (G) and *Bnp* (H), and fibrotic markers, *Collagen1a1* (I) and *Collagen3a1* (J) mRNA expression in mouse hearts were determined by qRT-PCR. Data are shown as mean ± SD of at least 3 independent experiments, one-way ANOVA. *denotes the P< 0.05, **denotes the P < 0.01 and ***denotes the P < 0.001 for the indicated comparisons. n.s. = not significant.

Echocardiographic measurement was used to determine the structures and hemodynamics of left ventricle and aortic arch with heart rate being kept to around 500 bpm in all four groups (i.e. WT-Sham, WT-TAC, Sirt5 KO-Sham, Sirt5 KO-TAC) (Table 1). In WT groups, peak flow velocity at aortic banding site which serves as the most effective predictor for successful TAC [50], was increased around by 4-fold at 5 weeks after TAC surgery (from 790.2 ± 50.1 to 3414.6 ± 6.0 mm/s, n = 9; P<0.001). In Sirt5 KO groups, peak flow velocity was also increased about by 4-fold after TAC surgery (from 845.9 ± 33.4 to 3386.5 ± 29.8 mm/s, n = 10; P<0.001). Thus, Sirt5 KO and WT mice were exposed to similar left ventricular end-systolic pressure overload. At 5 weeks after TAC, the heart weight/body weight (HW/BW) ratio was increased by 1.7-fold (P<0.001) and 1.5-fold (P<0.001) in WT and Sirt5 KO mice, respectively (S15A Fig and Fig 4A), reaffirming that the TAC surgery is successful. The HW/BW ratio was moderately decreased in Sirt5 KO mice compared to WT mice after TAC surgery, but failed to reach statistical significance (Fig 4A).

As expected, most of hypertrophic parameters were generally increased in WT mice after TAC surgery, including the left ventricular diastolic anterior wall thickness (LVAWTd) (increased by 1.6-fold; P<0.001), the LV systolic anterior wall thickness (LVAWTs) (increased by 1.4-fold; P<0.001), the LV diastolic posterior wall thickness (LVPWTd) (increased by 1.6-fold; P<0.001), and the LV systolic posterior wall thickness (LVPWTs) (increased by 1.4-fold; P<0.01) (Fig 4C–4F). These hypertrophic parameters tended to be lower in Sirt5 KO mice compared to WT controls after TAC surgery, but failed to reach statistical significance (Fig 4C–4F). Notably, left ventricular mass (LV mass) and LV mass/body weight (LV mass/BW) ratio were significantly decreased by 24% (P<0.001) and 20% (P<0.01), respectively, in Sirt5 KO mice compared to WT controls after TAC (Table 1 and Fig 4B). The discrepancy between LV mass and the HW/BW ratio is likely due to less changes in the right ventricle [34]. The pattern of change in other hallmarks of hypertrophic cardiomyopathy (e.g. *Anp*, *Bnp* mRNA) and cardiac fibrotic markers (e.g. *Collagen 1a1* and *Collagen 3a1* mRNA) was comparable between Sirt5 KO and WT mice at 5 weeks after TAC (Fig 4G–4J).

**Fig 5:**
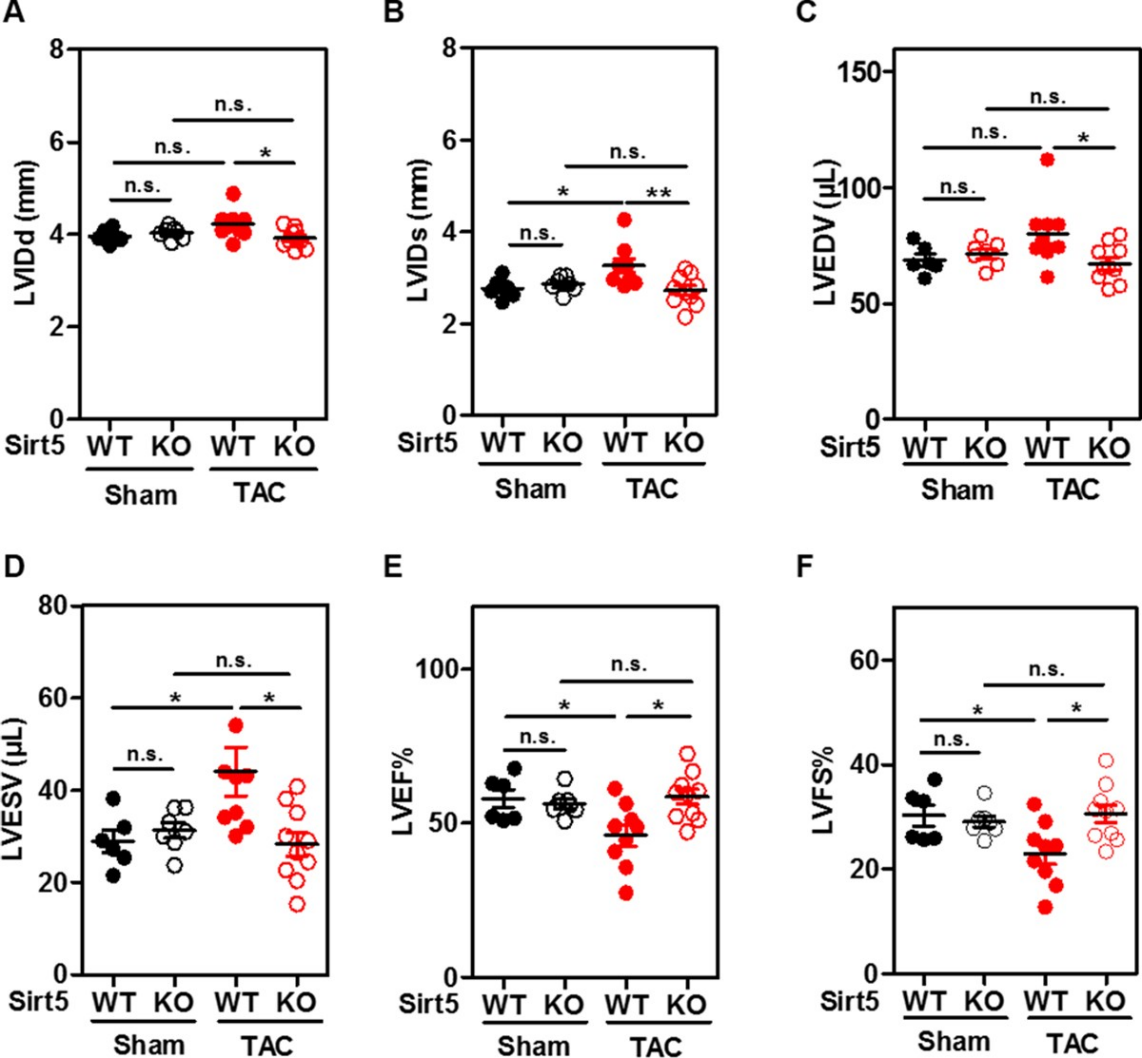
Sirt5 KO ameliorates cardiac dysfunction caused by TAC. (A-F) Left ventricular cardiac functional parameters between male Sirt5 KO mice and WT littermates at 5 weeks after TAC surgery (n = 6–10 animals per group), including left ventricular internal diastolic diameter (LVIDd) (A), left ventricular internal systolic diameter (LVIDs) (B), left ventricular end-diastolic volume (LVEDV) (C), left ventricular end-systolic volume (LVESV) (D), left ventricular ejection fraction LVEF% (E), and left ventricular shortening fraction LVFS% (F). Data are shown as mean ± SD of at least 3 independent experiments, one-way ANOVA. *denotes the P< 0.05 and **denotes the P < 0.01 for the indicated comparisons. n.s. = not significant.

Moreover, cardiac function was evaluated in WT and Sirt5 KO mice after TAC surgery. In WT groups, left ventricular internal diastolic diameter (LVIDd) and left ventricular internal systolic diameter (LVIDs) were increased by 1.1-fold (n.s.) and 1.2-fold (P<0.05) after TAC, respectively (S15B Fig, Fig 5A and 5B). Interestingly, neither LVIDd nor LVIDs was substantially changed in Sirt5 KO mice after TAC surgery. As a result, Sirt5 KO mice exhibited significantly lower levels of LVIDd (reduced by 7%; P<0.05) and LVIDs (reduced by 16.4%; P<0.01) than WT mice after TAC surgery (Fig 5A and 5B). Accordingly, left ventricular end-diastolic volume (LVEDV) and left ventricular end-systolic volume (LVESV) were markedly reduced by 16.4% (P<0.05) and 36% (P<0.05), respectively, in Sirt5 KO mice compared to WT controls after TAC surgery (Fig 5C and 5D). Furthermore, left ventricular ejection fraction (LVEF%) and left ventricular fractional shortening (LVFS%), two important parameters for cardiac functions, were significantly decreased by 20.5% (P<0.05) and 24% (P<0.05), respectively, in WT mice after TAC surgery (Fig 5E and 5F). Such reductions in LVEF% and LVFS% were, however, not observed in Sirt5 KO mice after TAC (Fig 5E and 5F). Consequently, Sirt5 KO mice exhibited significantly higher levels of LVEF% (increased by 1.3-fold; P<0.05) and LVFS% (increased by1.3-fold; P<0.05) than WT mice after TAC surgery (Fig 5E and 5F).

### Sirt5 KO increases AMP/ATP ratio and promotes AMPK activation in TAC mice

**Fig 6:**
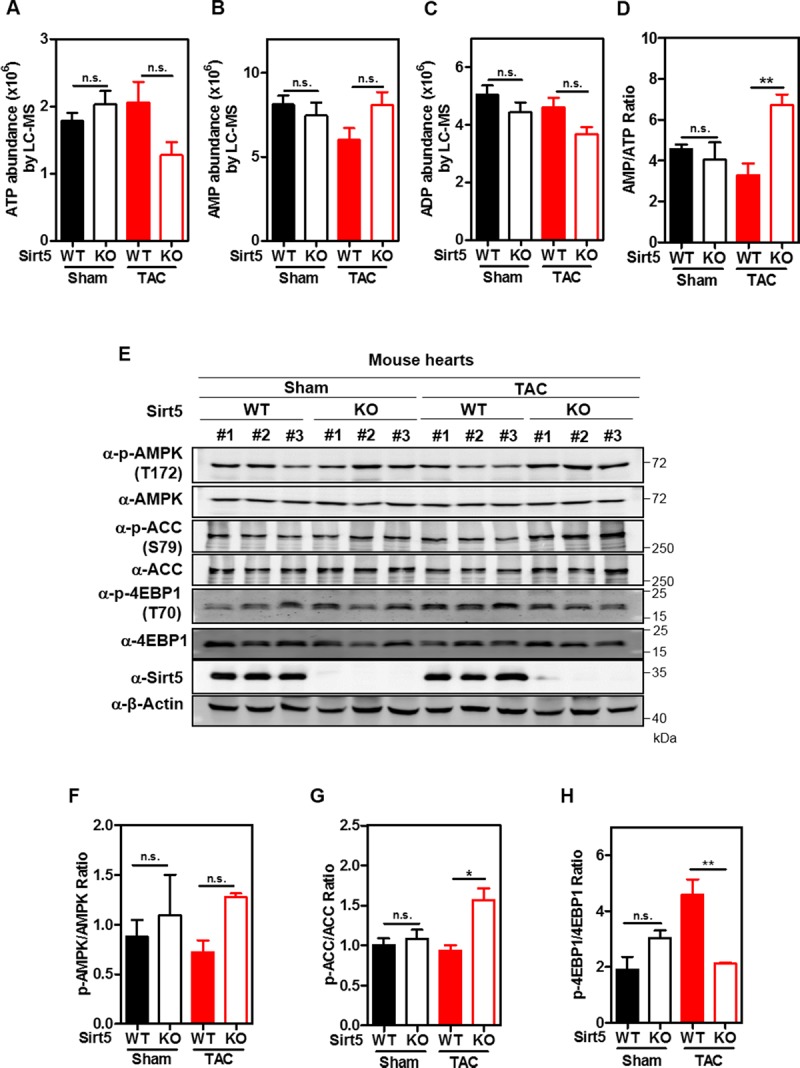
Sirt5 KO promotes AMPK activation in hearts of TAC mice. (A-D) Sirt5 deficiency suppresses ATP production in mouse hearts after TAC surgery. Relative levels of ATP (A), AMP (B), and ADP (C) in mouse hearts in both the Sham and TAC groups (n = 3 per group) were determined by LC-MS/MS analysis as described in ‘Materials and Methods’, and the AMP/ATP ratio was calculated accordingly (D). (E-H) Sirt5 KO promotes AMPK activation in mouse hearts after TAC surgery. Mouse heart samples were harvested as described above in (A-D), and AMPK T172 phosphorylation and ACC S79 phosphorylation were detected by immunoblotting using the indicated antibodies (E). Quantification of p-AMPK/AMPK ratio (F), p-ACC/ACC ratio (G), and p-4EBP1/4EBP1 ratio (H) were shown. Data are shown as mean ± SD of 3 independent experiments, one-way ANOVA. *denotes the P< 0.05, and **denotes the P < 0.01 for the indicated comparisons. n.s. = not significant.

In agreement with our earlier observations in cultured cells (Fig 1) and fasted mice (Fig 2), we found that relative ATP was reduced by 38% in hearts of Sirt5 KO mice compared to WT mice after TAC surgery (Fig 6A). A tendency to increased AMP was observed in hearts of Sirt5 KO mice compared to WT mice after TAC (Fig 6B). Moreover, relative ADP was reduced by 20.1% in hearts of Sirt5 KO mice compared to WT mice after TAC (Fig 6C). Although these changes failed to reach statistical significance, the AMP/ATP ratio was significantly increased by 1.5-fold (P<0.01) in hearts of Sirt5 KO mice compared to WT mice after TAC (Fig 6D). The observed higher AMP/ATP ratio was consistent with an increase in AMPK T172 phosphorylation (by ~1.4-fold) and ACC S79 phosphorylation (by ~1.4-fold; P<0.05) in hearts of Sirt5 KO mice after TAC (Fig 6E–6G). Eukaryotic initiation factor 4E binding protein 1 (4EBP1) is negatively regulated by AMPK activity via mTORC1 inhibition, and it can regulate global protein translation [49, 51]. We found that T70 phosphorylation of 4EBP1 was significantly reduced by 53.5% (P<0.01) in hearts of Sirt5 KO mice as compared to WT mice after TAC (Fig 6E and 6H). Hence, these findings support a notion that Sirt5 knockout promotes AMPK activation, which may hinder protein translation and prevent ventricular dilation and cardiac dysfunction in TAC mice.

## Discussion

The NAD^+^-dependent deacylase SIRT5 primarily localizes to the mitochondrial matrix [52, 53] and is broadly expressed in mouse tissues with the highest mRNA and protein expression in the heart, brain, liver, kidney, and muscle [53, 54]. Germline Sirt5 KO is well tolerated in mice [53, 55–57], and these animals exhibit no strong phenotypes with apparently normal size, body weight, food intake, mobility, and fertility under normal physiological conditions [53, 55–57]. Notably, SIRT5 loss may cause cellular dysfunction under certain stress or pathological condition. For instance, it was reported that Sirt5 KO mice exhibit more severe nigrostriatal dopaminergic degeneration and accelerated motor deficit after administration with the neurotoxin 1-methyl-4-phenyl-1,2,3,6-tetrahydropyridine (MPTP) [57]. Moreover, aged Sirt5 KO mice display mild cardiac hypertrophy and cardiac dysfunction [11]. We recently reported that low SIRT5 expression is associated with unfavorable prognosis for hepatocellular carcinoma cancer (HCC) patients, although whether SIRT5 downregulation contributes to HCC development remains to be addressed [58]. In primary MEFs, we previously found that Sirt5 deficiency leads to decreased cell proliferation and hypersensitivity to oxidative stress [20]. Mechanistically, SIRT5 modulates cellular NADPH production by desuccinylating isocitrate dehydrogenase 2 (IDH2) and deglutarylating glucose-6-phosphate dehydrogenase (G6PD), respectively, and therefore increases their activities [20].

Regarding the potential role of SIRT5 in regulating cellular energy metabolism, SIRT5 overexpression was found to increase ATP level in HepG2 liver hepatocellular cells [59], while Sirt5 deficiency was reported to decrease ATP in mouse hearts under fasting conditions [11]. In this study, we confirm that SIRT5 KO decreases ATP level in cultured cells and mouse hearts under energy stress conditions, reaffirming the crucial role of SIRT5 in controlling ATP homeostasis. Moreover, we show that SIRT5 KO increases mitochondrial NADH, reflecting impaired NADH oxidation through the respiratory chain and oxidative phosphorylation for electron transport and ATP synthesis. In eukaryotic cells, the ETC and oxidative phosphorylation with the terminal step being carried out by ATP synthase on the mitochondrial inner membrane are major sites for ATP production. In the term of SIRT5 regulating the electron transport chain, previous studies have reported that *SIRT5* knockdown in HEK293T cells leads to increased succinylation and enzymatic activation of SDHA, known as complex II of the respiratory chain [7]. However, another study reported that the complex II activity was reduced in Sirt5 KO mouse livers [19]. The activities of complex I, III, and IV of the electron respiratory chain were found to be unaffected by Sirt5 KO in mouse livers [19]. It was previously reported that SIRT5 interacts with ATP5E/5F1/5L/5O subunits of ATP synthase [60], but the biological function of this protein interaction remains unclear. A Sirt5 KO cardiac succinylome study has identified highly succinylated peptides and proteins, including ATP5O/5H/5B/5J/5F1 subunits of ATP synthase [44]. We show here that Sirt5 KO significantly decreases the enzymatic activity of ATP synthase in mouse heart mitochondria, and thus at least in part contributes to the decreased ATP production. In another study by Sadhukhan et al., Sirt5 KO mice, however, exhibit decreased ATP production without affecting cardiac ATP synthase activity [11]. The discrepancy is likely caused by different detection methods and calculation algorithms for ATP synthase activity. In the above mentioned work and our current study, ATP synthase activity was determined by monitoring the hydrolyzation of ATP to ADP, coupled with pyruvate kinase (PK) and lactate dehydrogenase (LDH) to oxidize NADH. We calculated the activity of ATP synthase by normalization with the activity of citrate synthase (as an internal control for mitochondrial contents), whereas Sadhukhan et al. determined the activity of ATP synthase by using a commercial kit following the similar principle without normalization with the citrate synthase activity.

The heart owns a very rapid and dynamic rate of ATP consumption, and thus a constant supply of ATP is essential for keeping proper heart functions. While this manuscript was in preparation, a study by Hershberger et al. reported that whole-body Sirt5 KO accelerates cardiac remodeling and reduces animal survival in pressure overloaded mice [61]. In the study by Hershberger et al., the suture was placed between the left carotid and the left axillary arteries. In the present study, the aortic arch was tied steadily between the origins of innominate artery and left common carotid artery. This means that LV end systolic press in two studies are different. Moreover, TAC surgery induced an increase of LV end systolic pressure to 197.0 ± 7.3 mmHg and 166.4 ± 9.5 mmHg in Sirt5 KO and WT mice, respectively, in the work by Hershberger et al. There is a great disparity in LV end systolic pressure (around 31 mmHg) between Sirt5 KO and WT mice after TAC surgery. In our present study, the degree of TAC-induced pressure overload as reflected by the peak flow velocity at aortic banding site, was achieved to similar levels between Sirt5 KO and WT mice (3386.5 ± 29.8 mm/s and 3414.6 ± 6.0 mm/s, respectively). In fact, cardiac responses and signaling pathways are different during graded degrees of pressure overload due to aortic constriction [62–64]. We speculate that the discrepancy between Hershberger’s and our studies may be due to different induction approaches and/or differential graded degrees of pressure overload in mouse hearts. Very recently, Hershberger et al. reported that inducible, cardiomyocyte-specific Sirt5 KO mice exhibit normal survival in response to chronic pressure overload via TAC surgery for 16 weeks [44]. In addition to acute cardiac hypertrophy and cardiac dysfunction, SIRT5 KO was also found to induce chronic heart failure with aging, as early as 8 weeks of age, which is associated with reduced ATP production [11]. However, we did not observe any defective hypertrophic cardiomyopathy in Sirt5 KO mice at 15 weeks of age, which was also not observed in Sirt5 KO mice at 12–21 weeks of age in Hershberger’s work [61]. It has to be noted that mice are on the 129/C57BL/6J background in the work by Hershberger et al. [61] and us, and the genetic background information of animals in the study by Sadhukhan et al. was not shown [11]. Furthermore, cardiac functional parameters were measured in male mice at 12–21 weeks of age at 4 weeks post TAC in the study by Hershberger et al. [61], while we conducted the measurement in animals at 15 weeks of age at 5 weeks post TAC. We speculate that the variety of experimental outcomes may due to different surgical methods and the time points for parameter measurement after TAC surgery, and thus the effect of SIRT5 deficiency on cardiac functions may be in a context-dependent manner.

Moreover, we show in this study that SIRT5 deficiency increases AMP/ATP ratio and enhances AMPK activation in cells and mouse hearts under energy stress conditions. The protective effect of AMPK activation against cardiac hypertrophy and dysfunction has been well-recognized. For example, AMPKα2 KO significantly exacerbates left ventricular hypertrophy and dysfunction in mice after TAC surgery, which is associated with increase of 4EBP1 phosphorylation [49]. Moreover, cardiac-specific deletion of LKB1, a master serine/threonine kinase to phosphorylate and activate AMPK [65], leads to cardiac hypertrophy and dysfunction in mice, accompanied by reduced AMPK phosphorylation and increased phosphorylation of p70S6 kinase, which can regulate global protein synthesis [49, 66]. On the other hand, overexpression of a constitutively active form of AMPK can ameliorate LKB1-silencing induced hypertrophy in cultured neonatal rat cardiac myocytes [66]. Point mutation of Prkag2, the γ2 subunit of AMPK, causes cardiac hypertrophy in a transgenic mouse model, accompanied by dramatically increased phosphorylation of 4EBP1 [67]. Furthermore, long-term activation of AMPK by administration of AICAR, a specific activator of AMPK, attenuates protein synthesis, TAC-induced cardiac hypertrophy, and improves cardiac function in rats [68]. AMPK activation by globular adiponectin, an adipose tissue-derived hormone, alleviates AngII-induced cardiac hypertrophy and fibrosis in neonatal Sprague-Dawley rat [69]. These findings thus support the notion that AMPK exerts a pivotal role in cardiac protection partially through inhibition of protein translation. In this study, we show that Sirt5 KO attenuates TAC-induced cardiac hypertrophy and improves cardiac function in mice, which is associated with higher AMP/ATP ratio and AMPK activation, confirming the heart-protective role of AMPK activation in a mouse model of pressure overload-induced left ventricular hypertrophy. We believe that reduced ATP and increased AMP/ATP ratio in Sirt5 KO mouse hearts after TAC surgery is possibly a compensatory effect to activate AMPK, thereby attenuating the progression of cardiac remodeling and improving cardiac function.

In summary, we show in this study that SIRT5 deficiency suppresses mitochondrial NADH oxidation and ATP production at least in part through increasing lysine succinylation and enzymatic inhibition of ATP synthase, thereby contributing to decreased ATP production, higher AMP/ATP ratio, and enhanced AMPK activation in both cultured cells and an animal model for pressure overload-induced left ventricular hypertrophy. The higher AMP/ATP ratio and AMPK activation may contribute to cardiac protection under stress. Our study thus uncovers a crucial role of SIRT5 in regulating cellular energy metabolism and AMPK activation in response to energy stress.

## Supporting information

S1 Fig Verification of SIRT5 KO in HEK293T cells. Clone SIRT5 KO-#1: the two alleles both contained 20 nucleotides deletion, and allele-2 had a site mutation (blue). Clone SIRT5 KO-#2: one allele contained two nucleotide insertion and one site mutation (red and blue), while the other allele contained one nucleotide insertion (red). Clone SIRT5 KO-#3: each allele contained four and one nucleotide deletion, respectively.
(TIF)

S2 Fig Application and identification of genetically encoded sensors to detect NADH in the cytosol and mitochondria. FrexH and Frex were ectopically expressed in HEK293T cells, and their subcellular localizations were determined by immunofluorescence staining in the presence of 40 μM NADH. Representative immunofluorescence images (original magnification, 630 x; a single focal plane, scale bar, 5 μm) are shown.
(TIF)

S3 Fig SIRT5 KO and WT HEK293T cells are separated into two clusters especially at 72 hours of culture periods. Principal component analysis was performed to analyze the indicated intermediates in SIRT5 WT, SIRT5 KO-#1 and SIRT5 KO-#2 HEK293T cells. In the score plot, SIRT5 KO and WT cells were separately clustered, especially at 72 hours after plating. n = 3 or 4 for each cell line.
(TIF)

S4 Fig SIRT5 KO changes intracellular metabolites in HEK293T cells. Principal component analysis was performed to analyze the indicated intermediates in SIRT5 WT, SIRT5 KO-#1 and SIRT5 KO-#2 HEK293T cells. In the loading plot, p1 is for distinguishing 16, 48, and 72 hours of plating, and p2 is for distinguishing WT and KO cells. Metabolites in the upper right panel of the plot changed significantly, including ATP. n = 3 or 4 for each cell line.
(TIF)

S5 Fig SIRT5 KO and WT HEK293T cells are separated into two clusters at 16 hours of culture periods. Orthogonal projections to latent structure-discriminant analysis was performed to analyze the indicated intermediates in SIRT5 KO-#1 and WT HEK293T cells (1×10^6^ cells) at 16 hours after plating. n = 3 or 4 for each cell line.
(TIF)

S6 Fig SIRT5 KO changes intracellular metabolites at 16 hours of culture periods in HEK293T cells. The volcano plots showed the fold change (log2) of mean concentrations of metabolites in SIRT5 KO-#1 and WT cells at 16 hours after plating according to Student’s t test p values (-log10), n = 3 or 4 for each cell line.
(TIF)

S7 Fig SIRT5 KO and WT HEK293T cells are separated into two clusters at 72 hours of culture periods. Orthogonal projections to latent structure-discriminant analysis was performed to analyze the indicated intermediates in SIRT5 KO-#1 and WT HEK293T cells (1×10^6^ cells) at 72 hours after plating. n = 3 or 4 for each cell line.
(TIF)

S8 Fig SIRT5 KO changes intracellular metabolites at 72 hours of culture periods in HEK293T cells. The volcano plots showed the fold change (log2) of mean concentrations of metabolites in SIRT5 KO-#1 and WT cells at 72 hours after plating according to Student’s t test p values (-log10), n = 3 or 4 for each cell line.
(TIF)

S9 Fig Putting-back SIRT5 cannot attenuate the increased phosphorylation of AMPK in SIRT5 KO HEK293T cells. HA-SIRT5 was ectopically expressed in SIRT5 KO HEK293T. Cells were collected at the indicated culture periods, and immunoblotting was performed with the indicated antibodies (A). Moreover, HA-SIRT5^H158Y^ was ectopically expressed in SIRT5 KO HEK293T. Cells were collected after glucose and glutamine starvation for 1 hour, and then immunoblotting was performed with the indicated antibodies (B).
(TIF)

S10 Fig *SIRT5* knockdown leads to increased AMP/ATP ratio and AMPK activation in HEK293T cells. (A-B) The AMP/ATP ratio is significantly increased in *SIRT5* knockdown HEK293T cells. 2×10^6^ cells were seeded into 60 mm plates. After culture for 72 hours, the cells were subjected to LC-MS/MS for metabolic profiling as described in ‘Materials and Methods’. Relative levels of ATP (A) and AMP/ATP ratio (B) were quantified. (C) AMPK activation in *SIRT5* knockdown HEK293T cells. Cells were collected at 72 hours, and AMPK T172 phosphorylation was detected by immunoblotting using the indicated antibody. (D-E) The AMP/ATP ratio is significantly increased in SIRT5 knockout HEK293T cell pool. 1×10^6^ cells were seeded into each well of six-well plates. After culture for 72 hours, the cells were subjected to LC-MS/MS for metabolic profiling as described in ‘Materials and Methods’. Relative levels of ATP (D) and AMP/ATP ratio (E) were quantified. (F) AMPK activation in SIRT5 knockout HEK293T cell pool. Cells were collected at 72 hours, and AMPK T172 phosphorylation was detected by immunoblotting using the indicated antibody. n = 3 for each cell line. Data are shown as mean ± SD of 3 independent experiments, two-tailed unpaired Student's t-test. *denotes the P < 0.05, **denotes the P < 0.01, and ***denotes the P < 0.001 for the indicated comparisons.
(TIF)

S11 Fig Sirt5 KO does not change lysine acetylation in mitochondria of mouse hearts. Male Sirt5 KO mice (n = 3) and WT control mice (n = 3) (16–28 weeks old) were fasted overnight. Upon sacrifice, mouse hearts were harvested for isolation of cardiac mitochondria. Immunoblotting was performed using the anti-acetyllysine antibody. Total protein loading was stained with Ponceau S.
(TIF)

S12 Fig Sirt5 KO leads to increased lysine succinylation of Atp5b in mouse heart mitochondria. (A) To verify the immunoprecipitation efficiency of ATP synthase antibody, ectopically expressed Flag-EGFP-ATP5B was included as a positive control. WCL, whole heart tissue lysate; Mito, mitochondria; CTRL, control. (B) Sirt5 deficiency increases lysine succinylation of Atp5b in hearts of fasted mice. Sirt5 KO mice (n = 4) and sex-matched WT control mice (n = 4) (12 weeks old) were fasted overnight. Upon sacrifice, mouse hearts were harvested for isolation of cardiac mitochondrion, and were then subjected to immunoprecipitation with the ATP synthase antibody, following immunoblotting to detect Atp5b and succinylation.
(TIF)

S13 Fig Verification of the methodology to measure ATP synthase activity. (A-B) Mouse heart mitochondria were isolated, and Oligomycin-sensitive activity of ATP synthase was measured with varying amount of mouse heart mitochondrial extracts as described in ‘Materials and Methods’. OL, oligomycin, a specific inhibitor of ATP synthase.
(TIF)

S14 Fig Sirt5 deficiency suppresses ATP synthase activity in hearts of fasted mice. (A-B) Sirt5 KO mice (n = 9) and sex-matched WT control mice (n = 6) (16–28 weeks old) were fasted overnight. Upon sacrifice, mouse hearts were harvested for isolation of cardiac mitochondrion. Immunoblotting was performed using the anti-SDHA antibody and anti-SIRT5 antibody. Total protein loading was stained with Ponceau S (A). The samples were then subjected to ATP synthase activity (B) assays as described in ‘Materials and Methods’. Data are shown as mean ± SD of at least 3 independent experiments, two-tailed unpaired Student's t-test. **denotes the P < 0.01 for the indicated comparison.
(TIF)

S15 Fig Gross images of mouse hearts and M-mode images of echocardiography in the hearts of Sirt5 KO mice and WT littermates in the Sham and TAC groups. (A-B) Gross images of mouse hearts in the Sham and TAC groups. All gross images of the hearts of Sirt5 KO mice and WT littermates in the Sham and TAC groups (n = 6–10 per group) were presented (A). M-mode images of echocardiography showing cardiac function in the hearts of Sirt5 KO mice and WT littermates in the Sham and TAC groups were shown (n = 6–10 per group) (B).
(TIF)

S1 Table Summary of previous proteomic studies on ATP synthase subunits. List of lysine succinylation, malonylation and glutarylation sites of mammalian ATP synthase subunits.
(ZIP)

S1 Dataset Original data for immunoblotting, graphs and Table 1. (ZIP)
