# Supplementary figures and images for: SIRT5 deficiency suppresses mitochondrial ATP production and promotes AMPK activation in response to energy stress
Source: PLoS One. 2019 Feb 13;14(2):e0211796. doi: 10.1371/journal.pone.0211796 (PMC6373945; doi:10.1371/journal.pone.0211796)

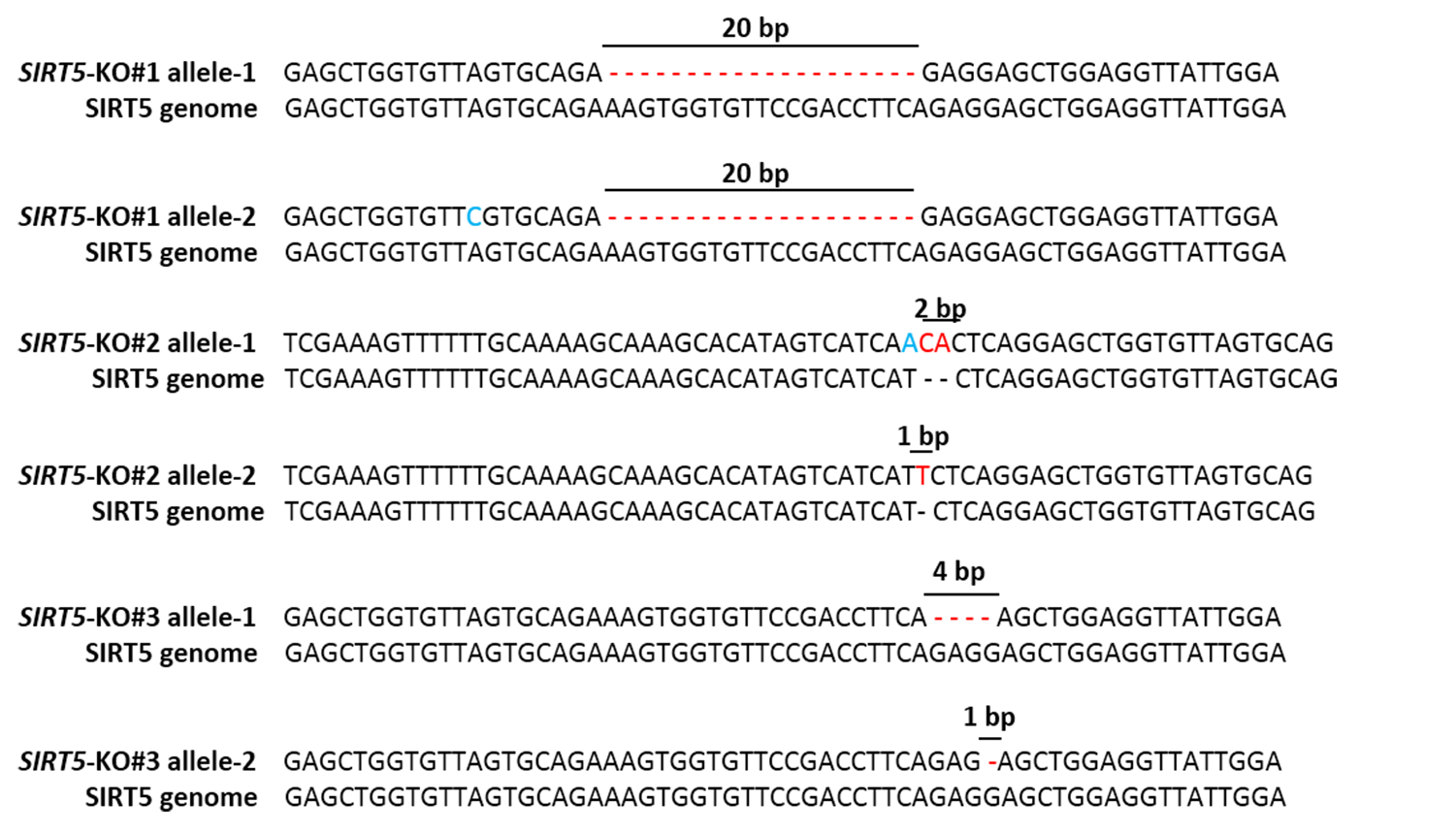

Supplement: S1 Fig — Clone SIRT5 KO-#1: the two alleles both contained 20 nucleotides deletion, and allele-2 had a site mutation (blue). Clone SIRT5 KO-#2: one allele contained two nucleotide insertion and one site mutation (red and blue), while the other allele contained one nucleotide insertion (red). Clone SIRT5 KO-#3: each allele contained four and one nucleotide deletion, respectively. (TIF) [file pone.0211796.s001.tif]

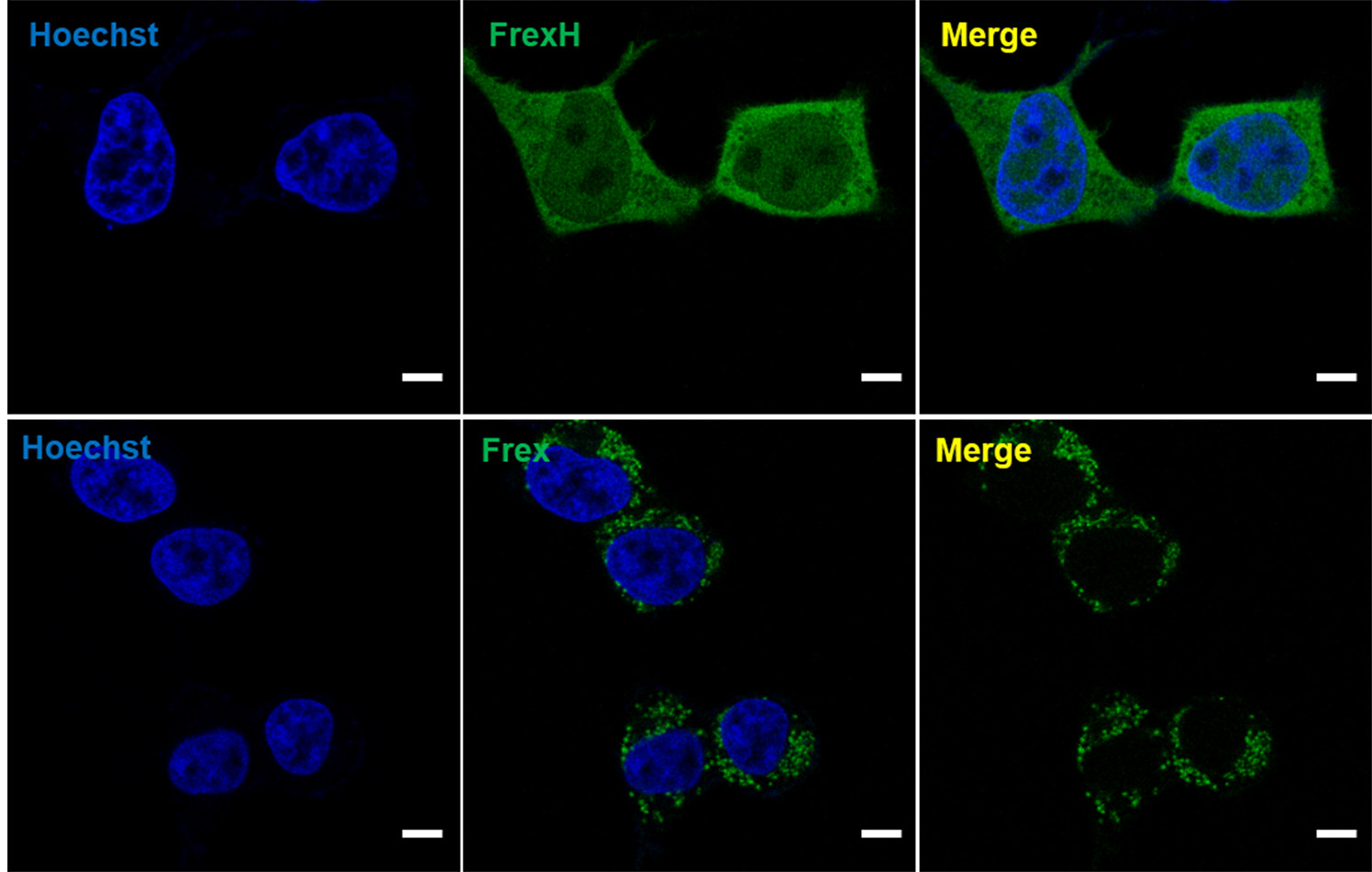

Supplement: S2 Fig — FrexH and Frex were ectopically expressed in HEK293T cells, and their subcellular localizations were determined by immunofluorescence staining in the presence of 40 μM NADH. Representative immunofluorescence images (original magnification, 630 x; a single focal plane, scale bar, 5 μm) are shown. (TIF) [file pone.0211796.s002.tif]

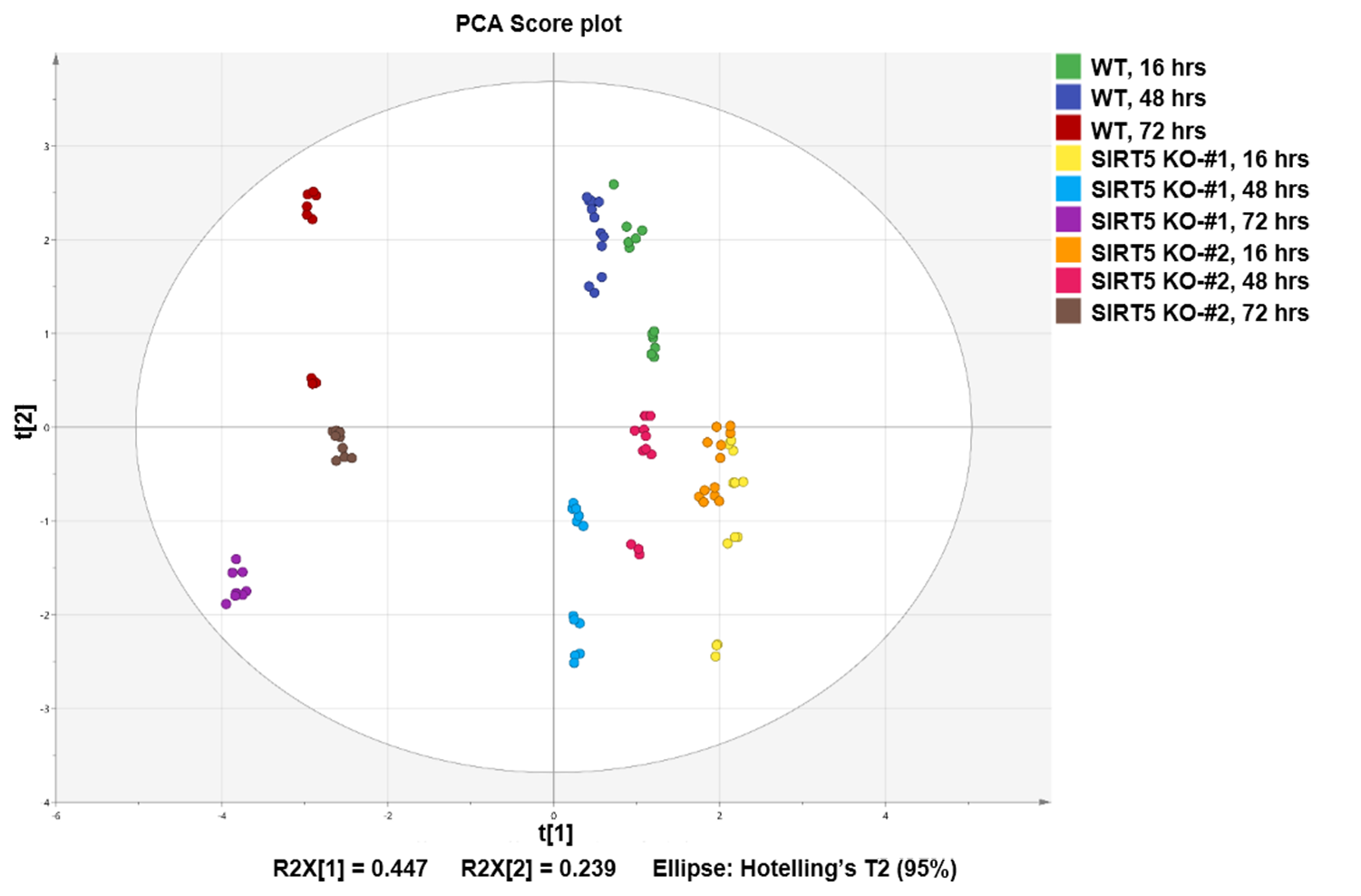

Supplement: S3 Fig — Principal component analysis was performed to analyze the indicated intermediates in SIRT5 WT, SIRT5 KO-#1 and SIRT5 KO-#2 HEK293T cells. In the score plot, SIRT5 KO and WT cells were separately clustered, especially at 72 hours after plating. n = 3 or 4 for each cell line. (TIF) [file pone.0211796.s003.tif]

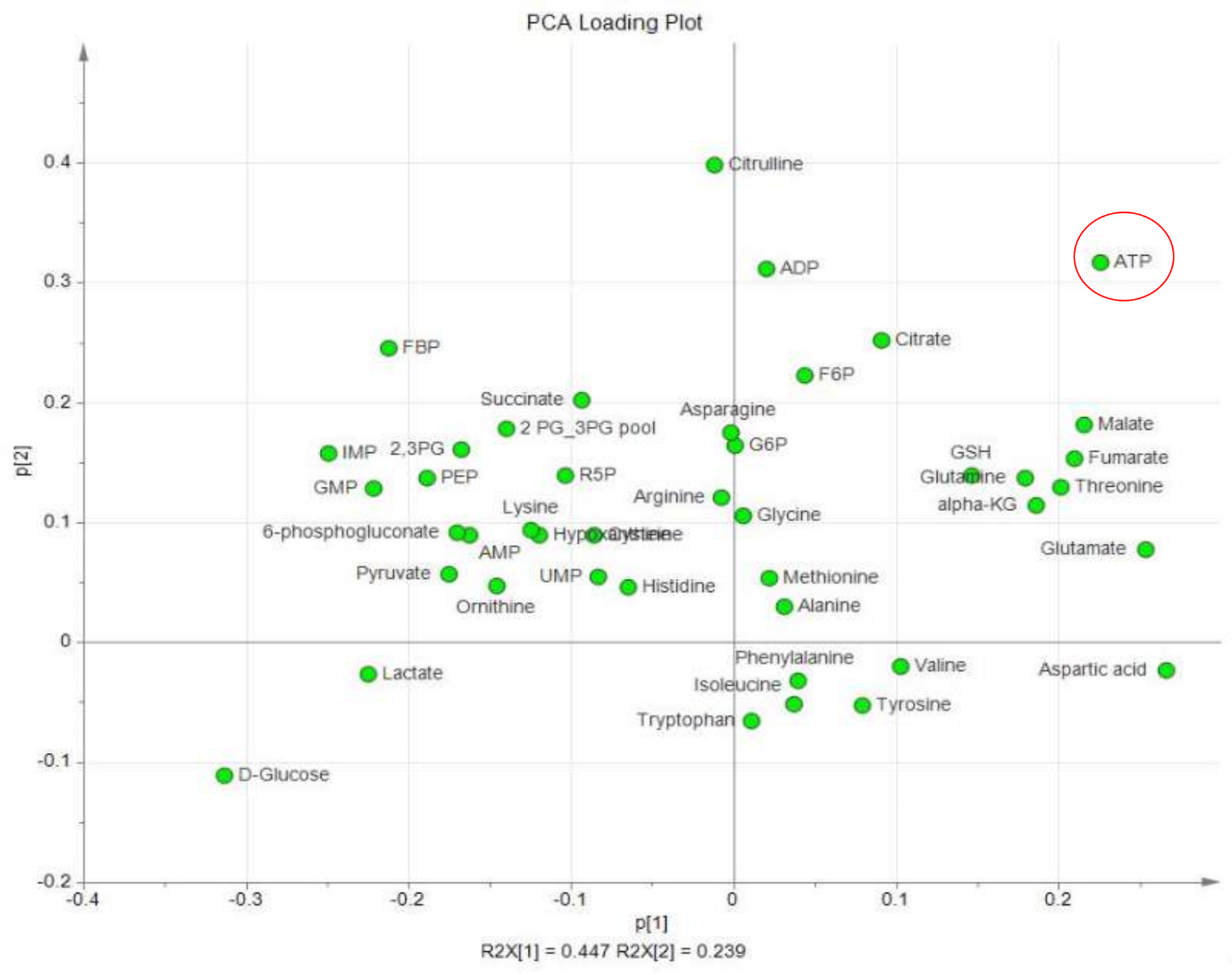

Supplement: S4 Fig — Principal component analysis was performed to analyze the indicated intermediates in SIRT5 WT, SIRT5 KO-#1 and SIRT5 KO-#2 HEK293T cells. In the loading plot, p1 is for distinguishing 16, 48, and 72 hours of plating, and p2 is for distinguishing WT and KO cells. Metabolites in the upper right panel of the plot changed significantly, including ATP. n = 3 or 4 for each cell line. (TIF) [file pone.0211796.s004.tif]

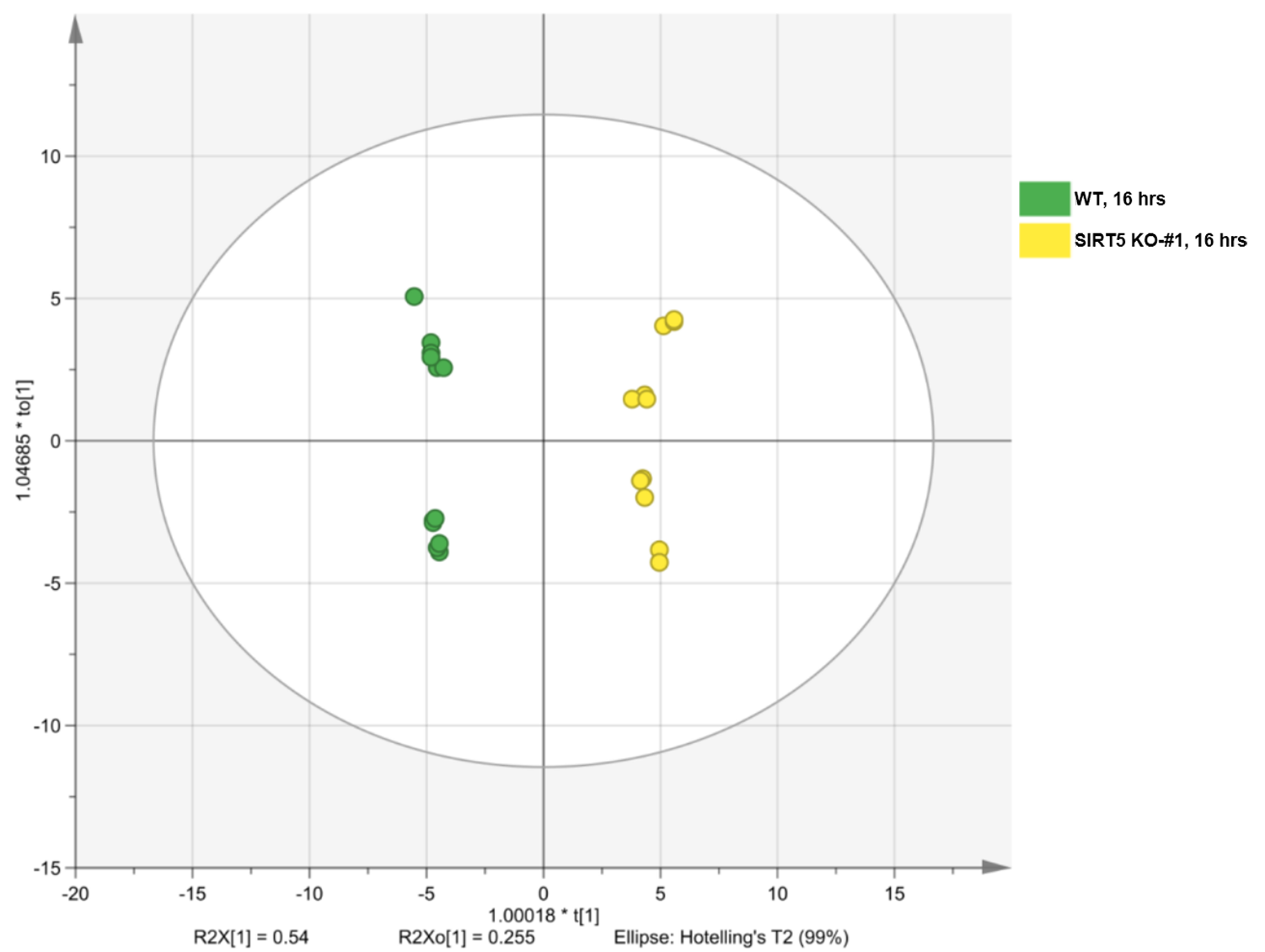

Supplement: S5 Fig — Orthogonal projections to latent structure-discriminant analysis was performed to analyze the indicated intermediates in SIRT5 KO-#1 and WT HEK293T cells (1×10^6^ cells) at 16 hours after plating. n = 3 or 4 for each cell line. (TIF) [file pone.0211796.s005.tif]

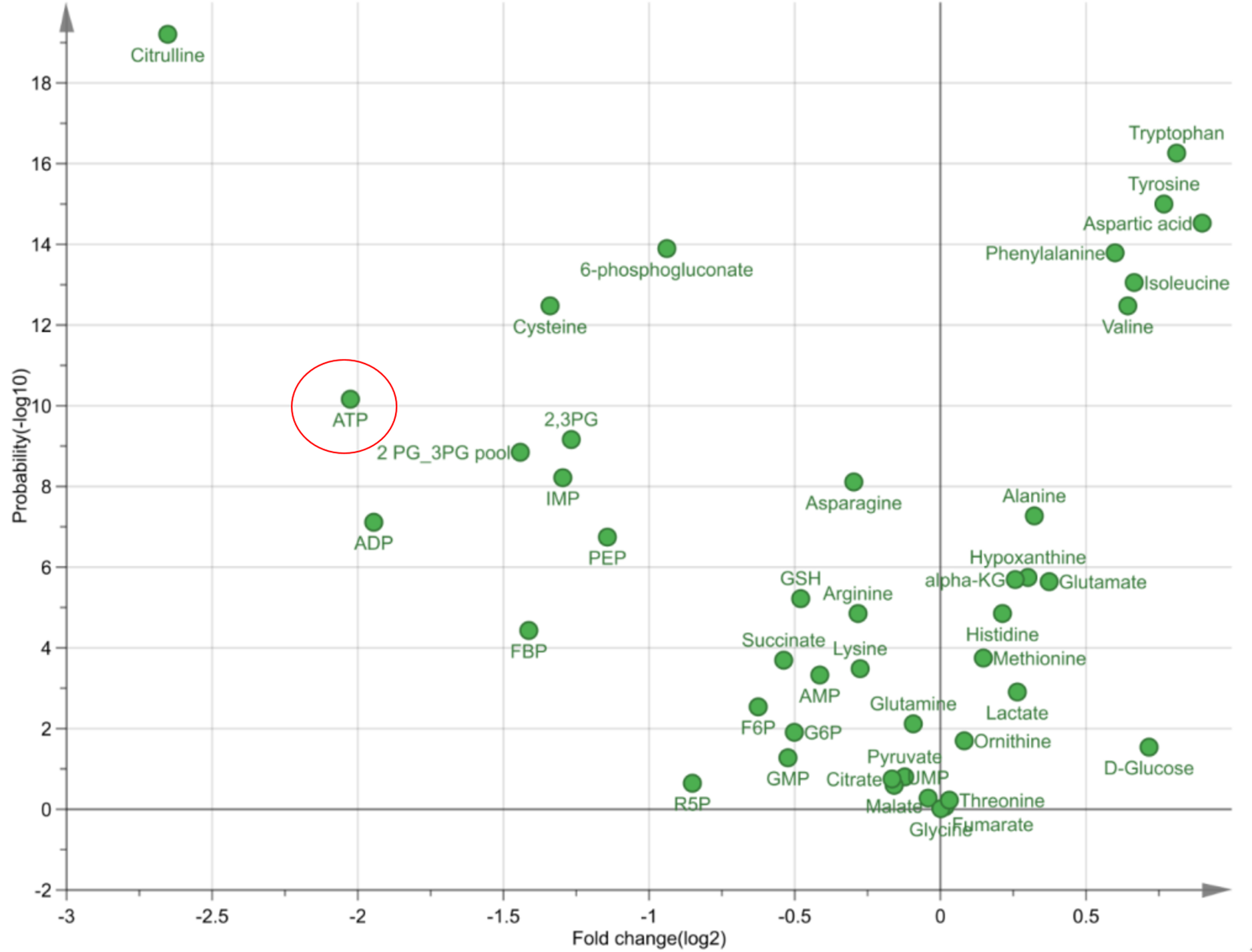

Supplement: S6 Fig — The volcano plots showed the fold change (log2) of mean concentrations of metabolites in SIRT5 KO-#1 and WT cells at 16 hours after plating according to Student’s t test p values (-log10), n = 3 or 4 for each cell line. (TIF) [file pone.0211796.s006.tif]

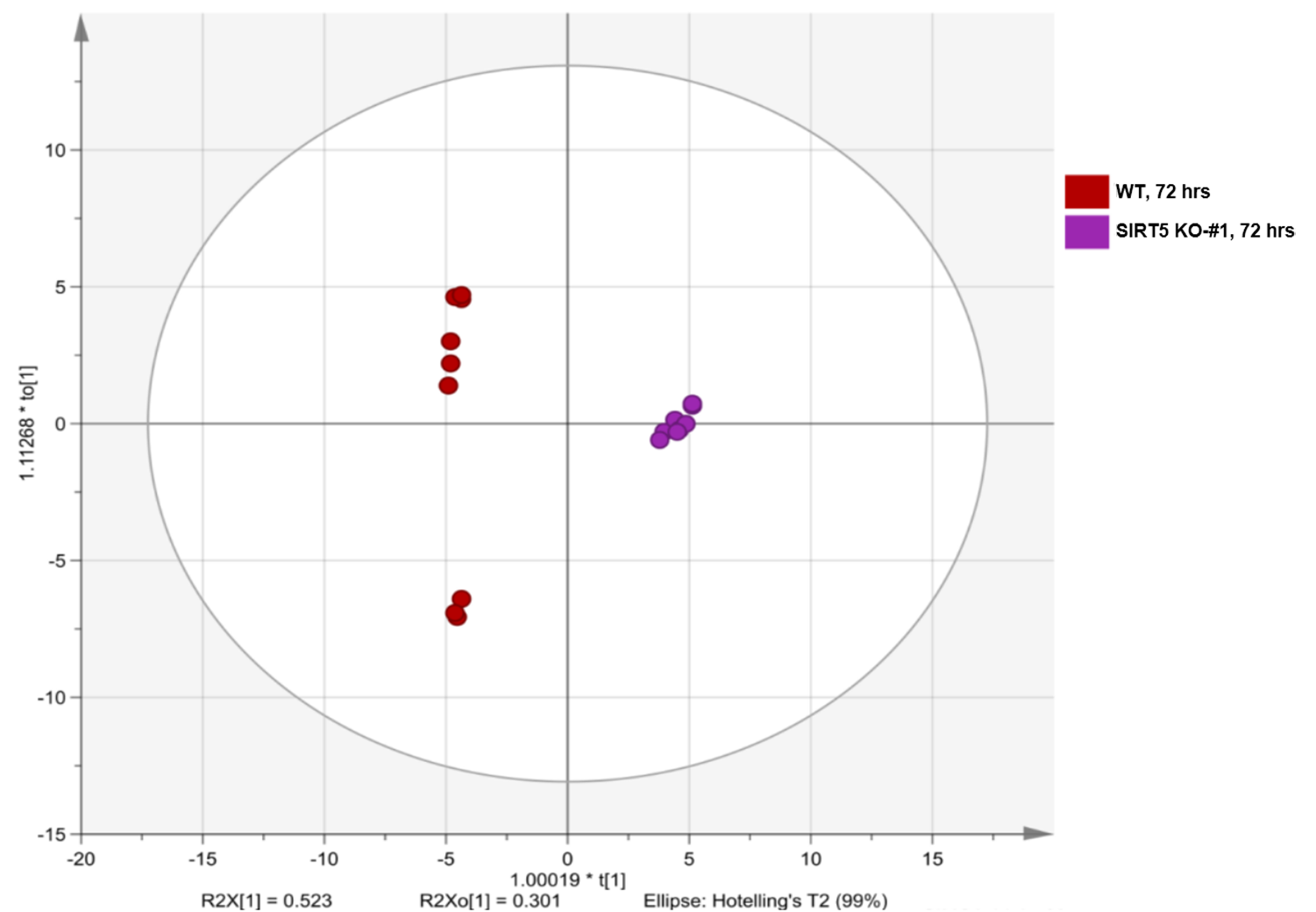

Supplement: S7 Fig — Orthogonal projections to latent structure-discriminant analysis was performed to analyze the indicated intermediates in SIRT5 KO-#1 and WT HEK293T cells (1×10^6^ cells) at 72 hours after plating. n = 3 or 4 for each cell line. (TIF) [file pone.0211796.s007.tif]

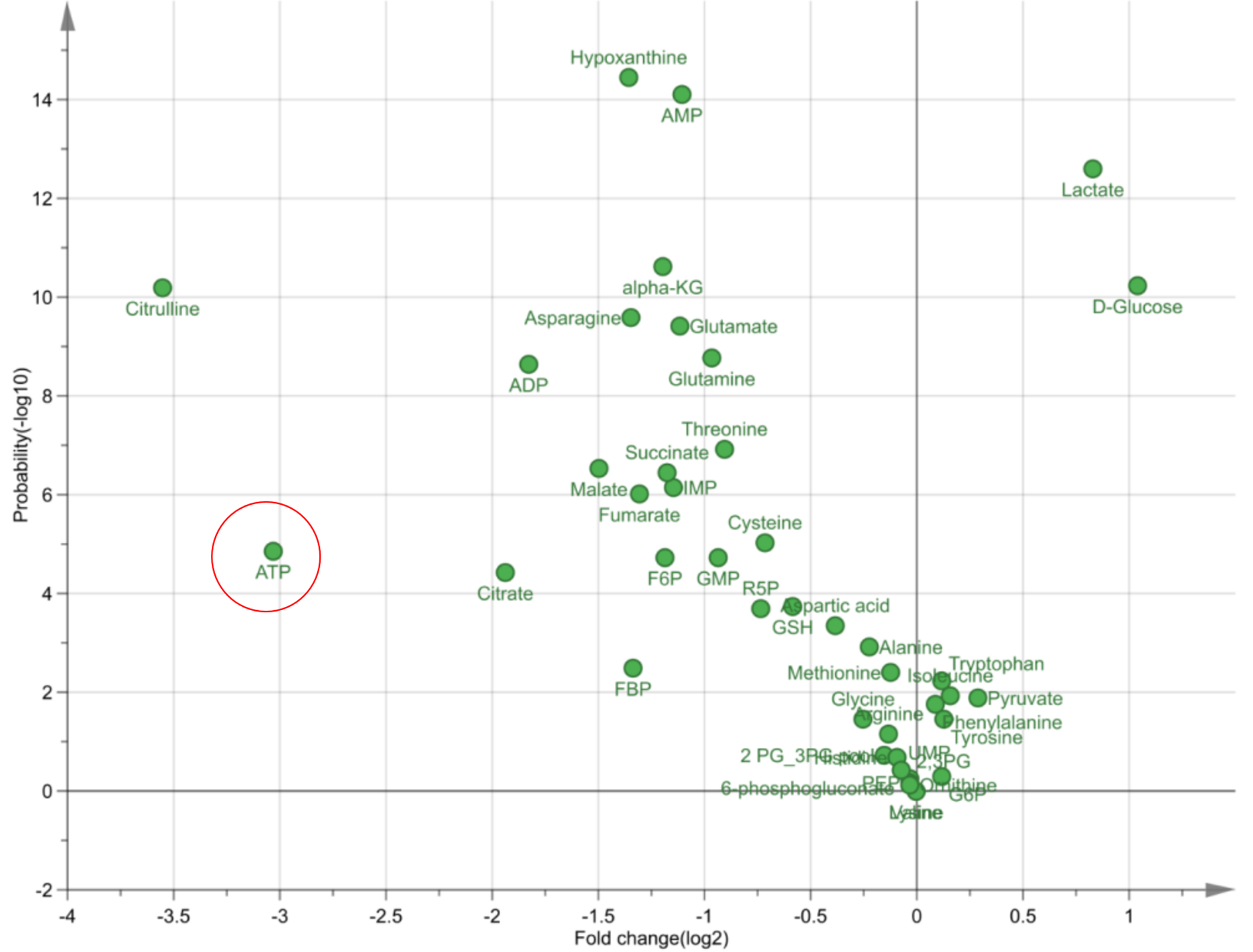

Supplement: S8 Fig — The volcano plots showed the fold change (log2) of mean concentrations of metabolites in SIRT5 KO-#1 and WT cells at 72 hours after plating according to Student’s t test p values (-log10), n = 3 or 4 for each cell line. (TIF) [file pone.0211796.s008.tif]

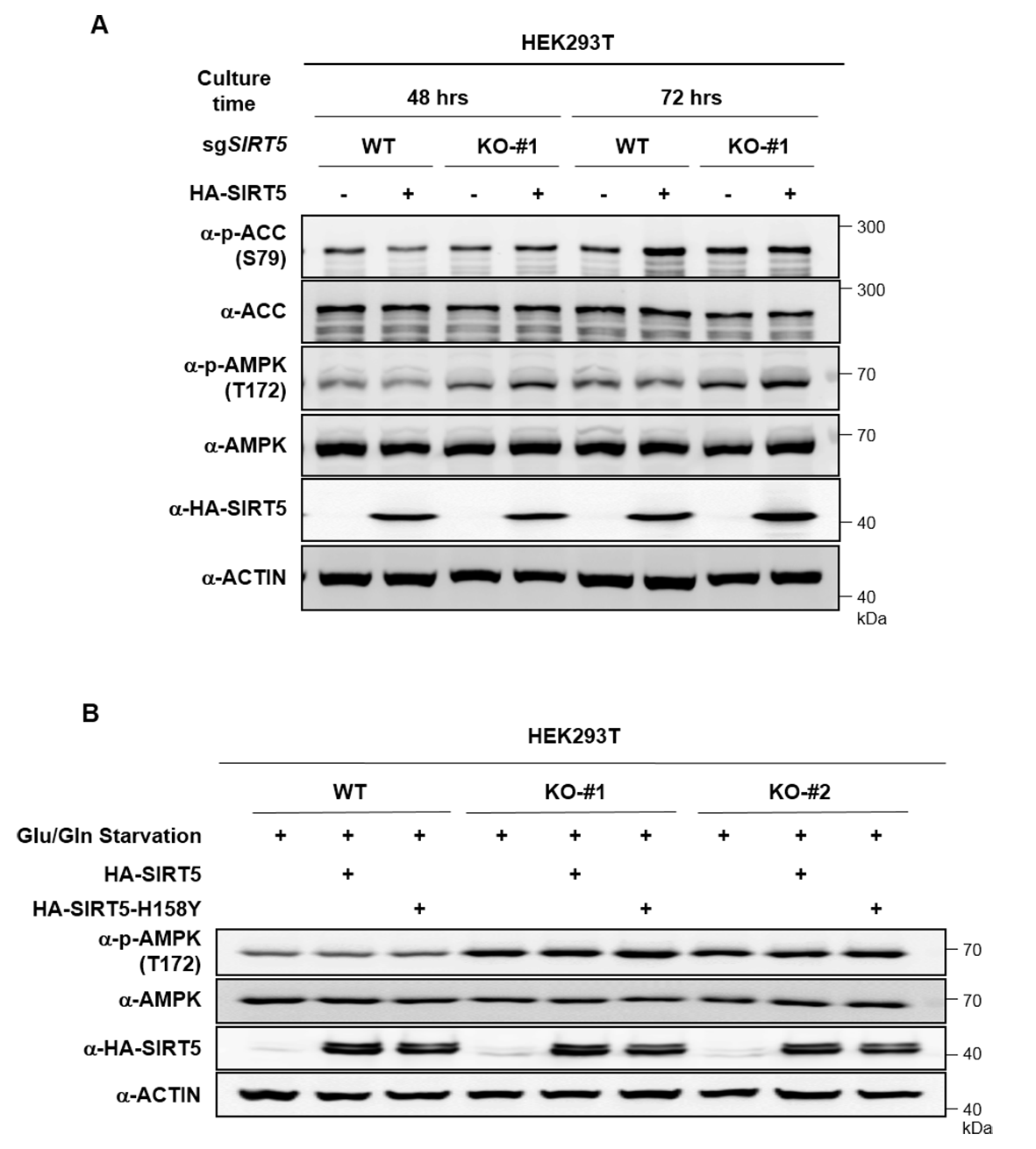

Supplement: S9 Fig — HA-SIRT5 was ectopically expressed in SIRT5 KO HEK293T. Cells were collected at the indicated culture periods, and immunoblotting was performed with the indicated antibodies (A). Moreover, HA-SIRT5H158Y was ectopically expressed in SIRT5 KO HEK293T. Cells were collected after glucose and glutamine starvation for 1 hour, and then immunoblotting was performed with the indicated antibodies (B). (TIF) [file pone.0211796.s009.tif]

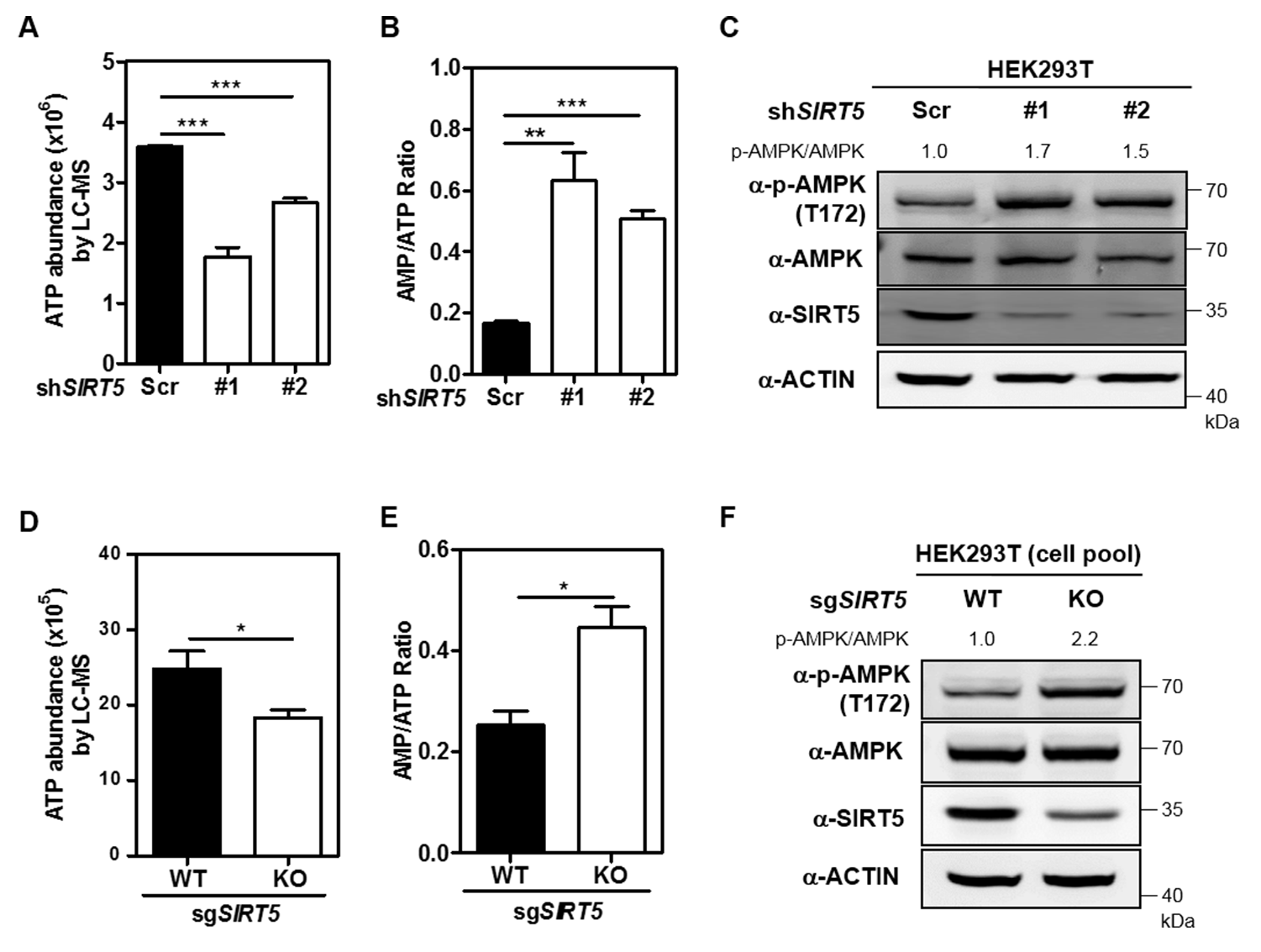

Supplement: S10 Fig — (A-B) The AMP/ATP ratio is significantly increased in SIRT5 knockdown HEK293T cells. 2×10^6^ cells were seeded into 60 mm plates. After culture for 72 hours, the cells were subjected to LC-MS/MS for metabolic profiling as described in ‘Materials and Methods’. Relative levels of ATP (A) and AMP/ATP ratio (B) were quantified. (C) AMPK activation in SIRT5 knockdown HEK293T cells. Cells were collected at 72 hours, and AMPK T172 phosphorylation was detected by immunoblotting using the indicated antibody. (D-E) The AMP/ATP ratio is significantly increased in SIRT5 knockout HEK293T cell pool. 1×10^6^ cells were seeded into each well of six-well plates. After culture for 72 hours, the cells were subjected to LC-MS/MS for metabolic profiling as described in ‘Materials and Methods’. Relative levels of ATP (D) and AMP/ATP ratio (E) were quantified. (F) AMPK activation in SIRT5 knockout HEK293T cell pool. Cells were collected at 72 hours, and AMPK T172 phosphorylation was detected by immunoblotting using the indicated antibody. n = 3 for each cell line. Data are shown as mean ± SD of 3 independent experiments, two-tailed unpaired Student's t-test. *denotes the P < 0.05, **denotes the P < 0.01, and ***denotes the P < 0.001 for the indicated comparisons. (TIF) [file pone.0211796.s010.tif]

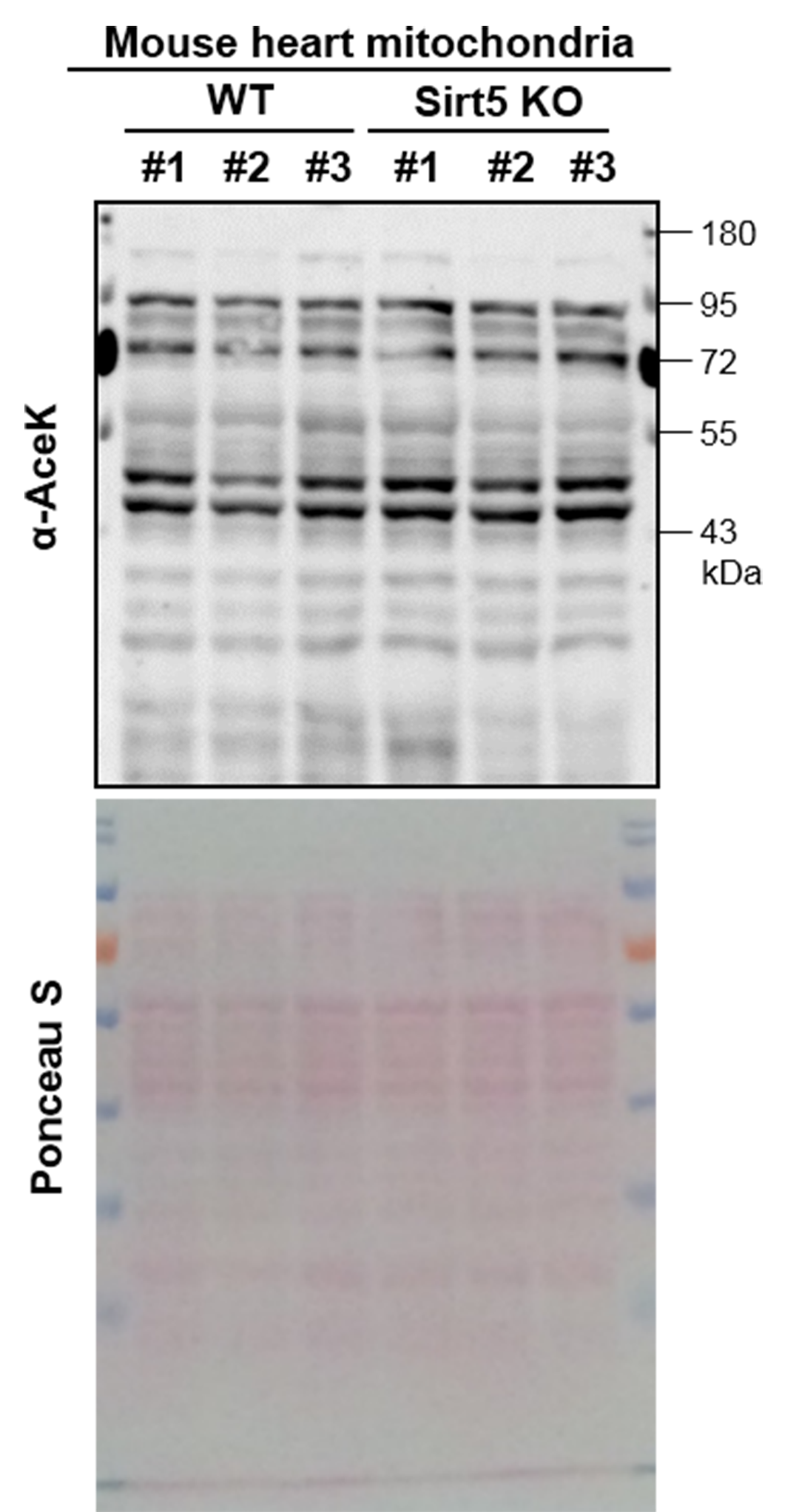

Supplement: S11 Fig — Male Sirt5 KO mice (n = 3) and WT control mice (n = 3) (16–28 weeks old) were fasted overnight. Upon sacrifice, mouse hearts were harvested for isolation of cardiac mitochondria. Immunoblotting was performed using the anti-acetyllysine antibody. Total protein loading was stained with Ponceau S. (TIF) [file pone.0211796.s011.tif]

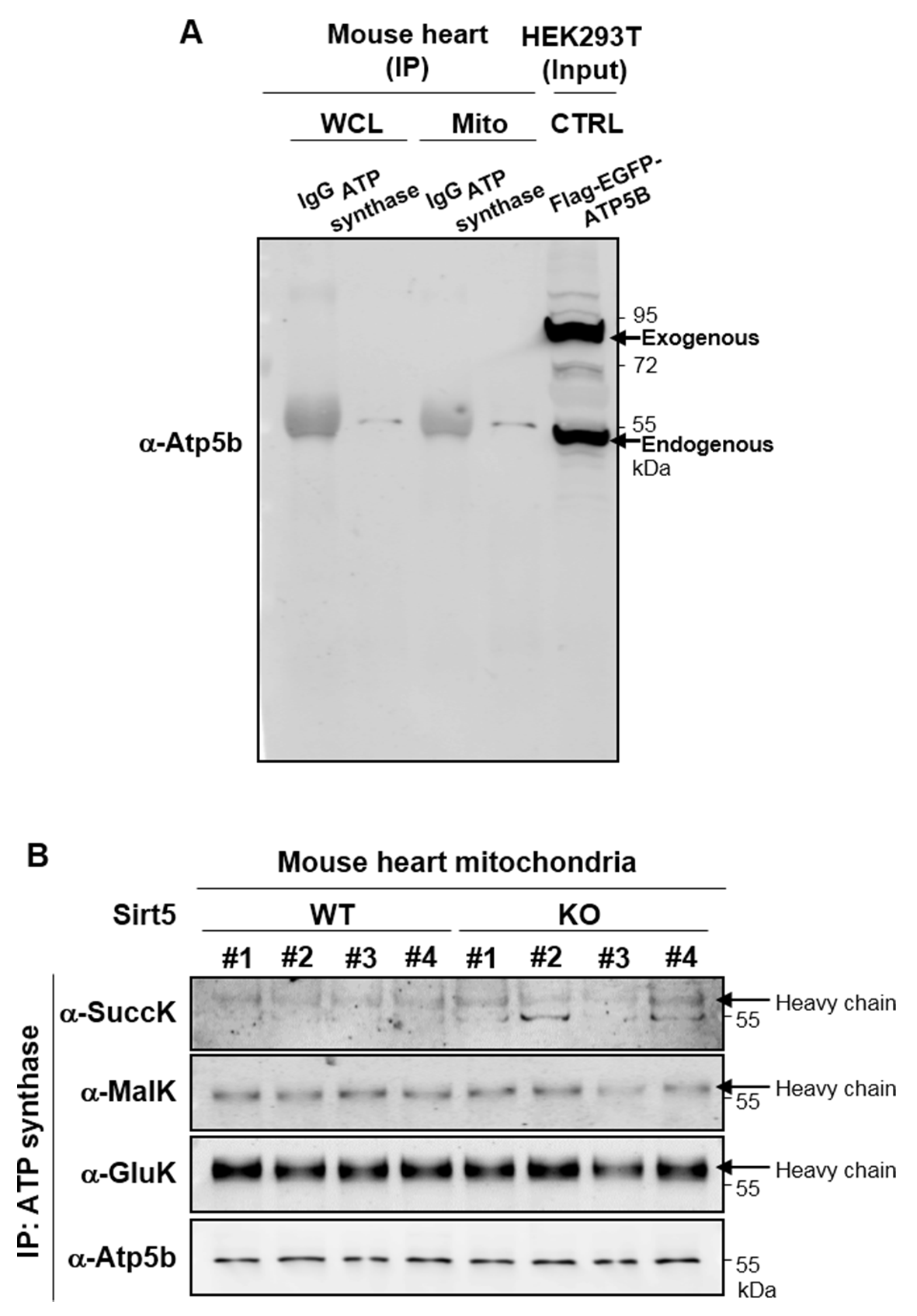

Supplement: S12 Fig — (A) To verify the immunoprecipitation efficiency of ATP synthase antibody, ectopically expressed Flag-EGFP-ATP5B was included as a positive control. WCL, whole heart tissue lysate; Mito, mitochondria; CTRL, control. (B) Sirt5 deficiency increases lysine succinylation of Atp5b in hearts of fasted mice. Sirt5 KO mice (n = 4) and sex-matched WT control mice (n = 4) (12 weeks old) were fasted overnight. Upon sacrifice, mouse hearts were harvested for isolation of cardiac mitochondrion, and were then subjected to immunoprecipitation with the ATP synthase antibody, following immunoblotting to detect Atp5b and succinylation. (TIF) [file pone.0211796.s012.tif]

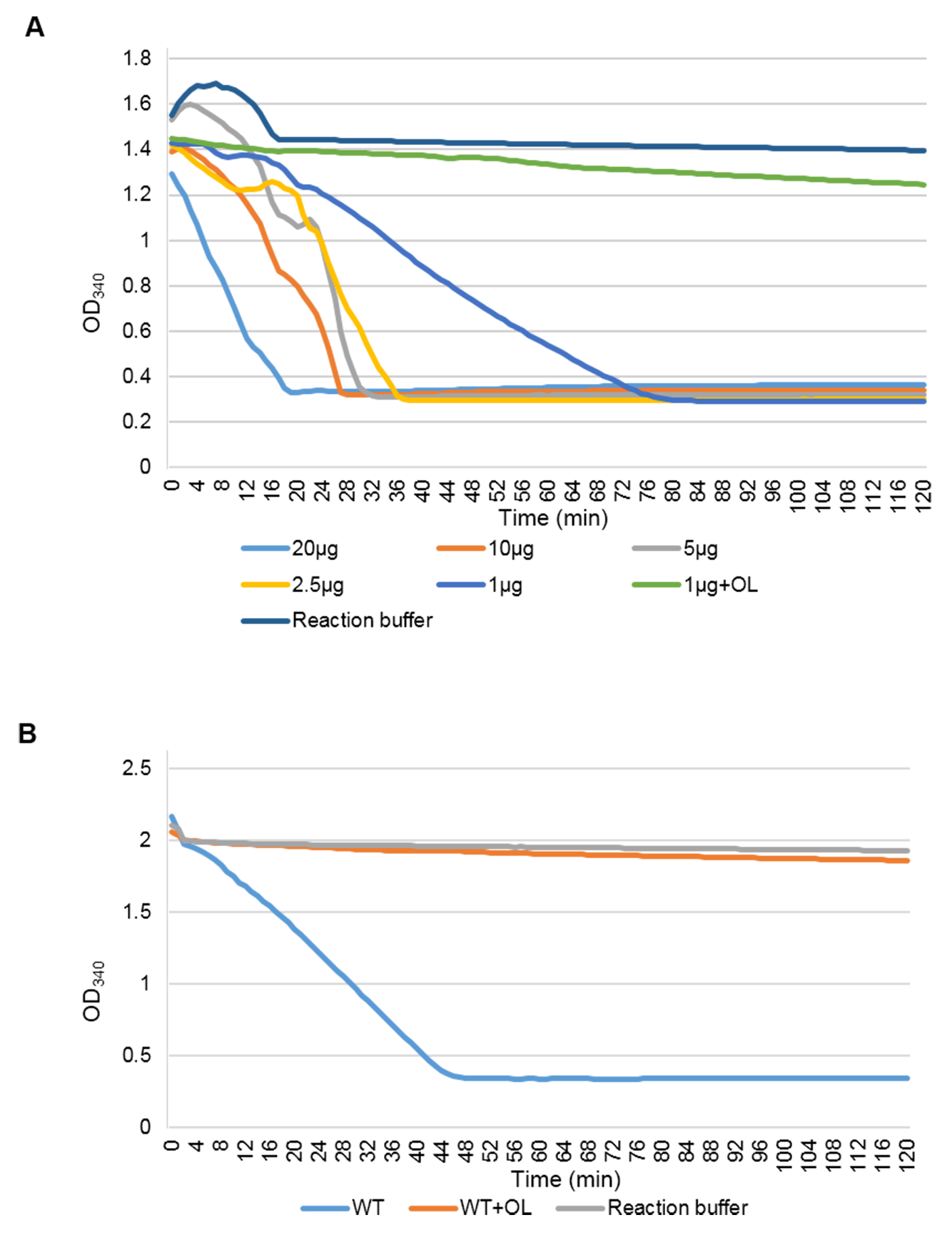

Supplement: S13 Fig — (A-B) Mouse heart mitochondria were isolated, and Oligomycin-sensitive activity of ATP synthase was measured with varying amount of mouse heart mitochondrial extracts as described in ‘Materials and Methods’. OL, oligomycin, a specific inhibitor of ATP synthase. (TIF) [file pone.0211796.s013.tif]

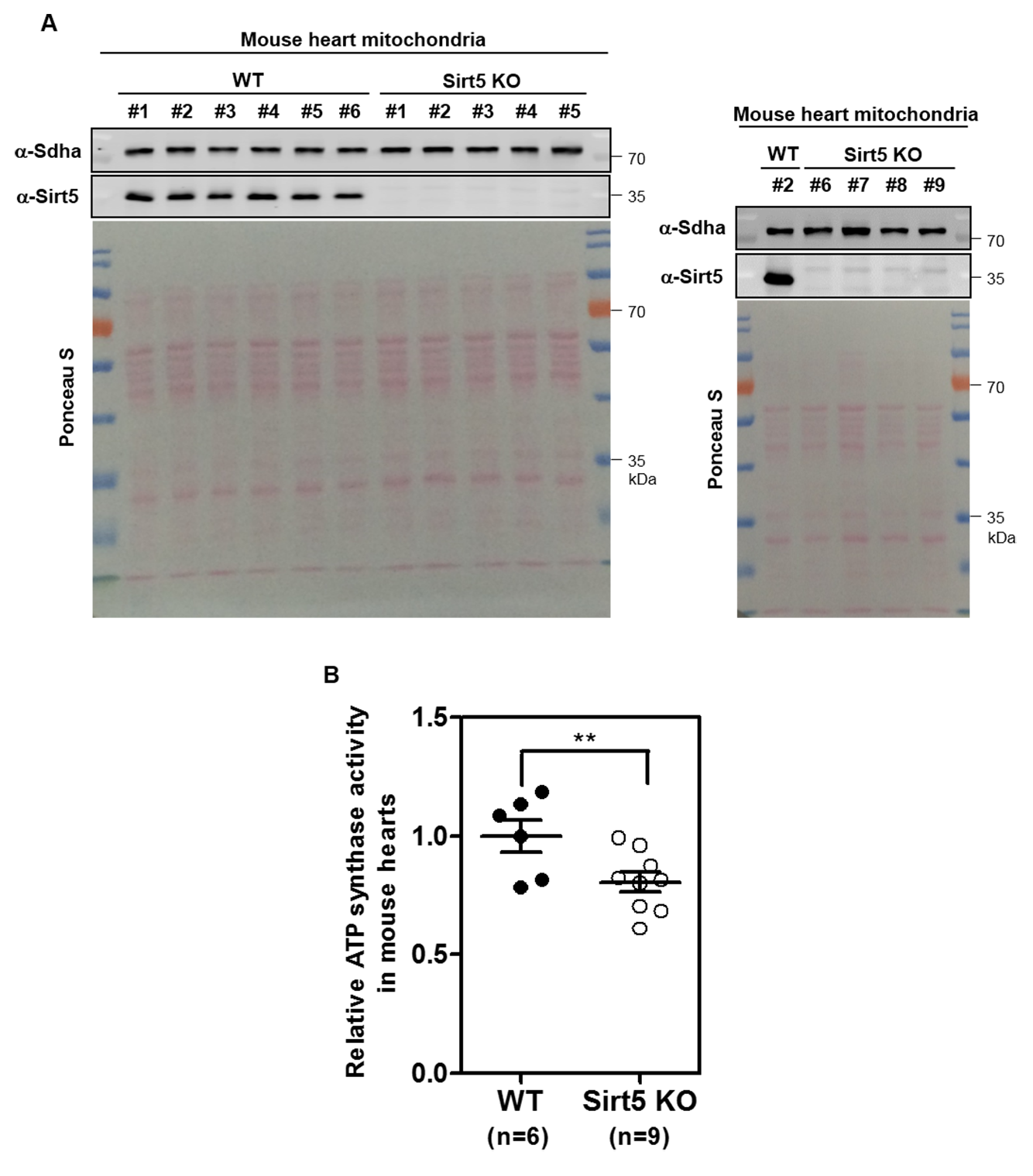

Supplement: S14 Fig — (A-B) Sirt5 KO mice (n = 9) and sex-matched WT control mice (n = 6) (16–28 weeks old) were fasted overnight. Upon sacrifice, mouse hearts were harvested for isolation of cardiac mitochondrion. Immunoblotting was performed using the anti-SDHA antibody and anti-SIRT5 antibody. Total protein loading was stained with Ponceau S (A). The samples were then subjected to ATP synthase activity (B) assays as described in ‘Materials and Methods’. Data are shown as mean ± SD of at least 3 independent experiments, two-tailed unpaired Student's t-test. **denotes the P < 0.01 for the indicated comparison. (TIF) [file pone.0211796.s014.tif]

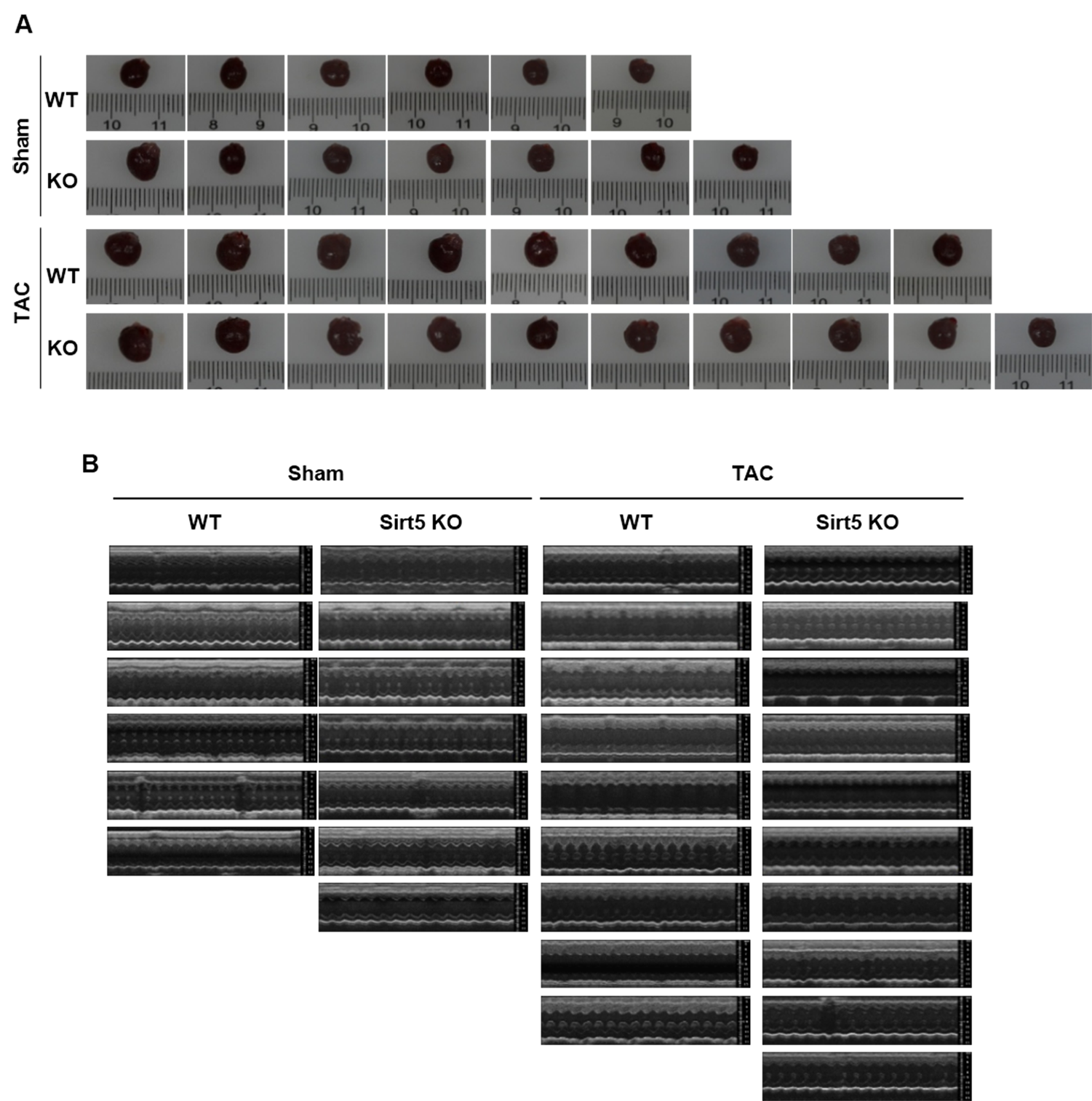

Supplement: S15 Fig — (A-B) Gross images of mouse hearts in the Sham and TAC groups. All gross images of the hearts of Sirt5 KO mice and WT littermates in the Sham and TAC groups (n = 6–10 per group) were presented (A). M-mode images of echocardiography showing cardiac function in the hearts of Sirt5 KO mice and WT littermates in the Sham and TAC groups were shown (n = 6–10 per group) (B). (TIF) [file pone.0211796.s015.tif]
