# Supplementary material for: SIRT5 deficiency suppresses mitochondrial ATP production and promotes AMPK activation in response to energy stress
Source: PLoS One. 2019 Feb 13;14(2):e0211796. doi: 10.1371/journal.pone.0211796 (PMC6373945; doi:10.1371/journal.pone.0211796)
Supplement: S1 Table — List of lysine succinylation, malonylation and glutarylation sites of mammalian ATP synthase subunits. (ZIP) [file pone.0211796.s016.zip › S1_Table.pdf]

ATP synthase subunit	SuccK (human)		SuccK (mouse)		MalK (human)	MalK (mouse)		GluK (human)		GluK (mouse)	
	Site	Ref	Site	Ref		Site	Ref	Site	Ref	Site	Ref
ATP5A1			45	[1, 2]	Not determined						
			76	[3]							
			82	[3]							
			31	[4]							
			37								
			123	[1, 2, 5]							
			125								
	126	[5]	126	[1, 2, 5, 6]		126	[7]				
			132	[2, 5, 6]							
	161	[5]	161	[1, 2, 4-6, 8]		161	[7]			161	[9]
	167	[5]	167	[2, 4, 5]		167	[7]				
			175	[2, 6]							
			180	[4]							
			189	[4]							
			194	[2]							
			23	[4]							
			218	[2]							
	230	[5]	230	[2, 4, 5]							
			231	[6]							
	239	[5]	239	[2, 4-6]						239	[9]
			240	[2]							
			241	[2]							
			252	[2]							
			255	[4]							
	261	[5]	261	[1, 2, 4-6]		261	[7]			261	[9]
			305	[1, 5]							
			316	[2]							
			377	[4]							
			384								
	427	[5]	427	[1, 2, 4-6, 8]		427	[7]			427	[9]
			434	[2]							

ATP synthase subunit	SuccK (human)		SuccK (mouse)		MalK (human)	MalK (mouse)		GluK (human)		GluK (mouse)	
ATP5A1			448	[4]	Not determined						
			456	[4]							
			472	[2]							
			481	[4]							
			489	[4]							
	498	[4]	498	[1, 2, 4-6, 8]		498	[7]			498	[9]
			503	[2]							
			506	[1, 2, 4]							
	531	[4]	531	[1, 2, 4-6]		531	[10]	531	[9]	531	[9]
	539	[4]	539	[1, 2, 4-6, 8]		539	[7, 10]			539	[9]
			541	[1, 2, 5, 6]							
ATP5B			55	[2]	Not determined						
			124	[1, 2, 4-6]		124	[7, 10]			124	[9]
	133	[4]	133	[1, 2, 4-6, 8]		133	[7, 10]			133	[9]
	159	[4]	159	[1, 2]							
			161	[1, 2, 4]							
	198	[4]	198	[1, 2, 4-6]						198	[9]
			201	[2]							
			212	[2]							
			225	[2]							
	259	[4]	259	[1, 2, 4-6, 8]							
			264	[1, 2, 4]							
			350	[2]							
			351	[2]							
	426	[4]	426	[1, 2, 5]		426	[7]				
			432	[2, 5]							
			451	[2]							
			480	[2, 6]							
	485	[4]	485	[1, 2, 4-6, 8]		485	[7]				
			489	[2]							
			519	[2]							
			522	[1, 2, 4]		522	[10]				

ATP synthase subunit	SuccK (human)		SuccK (mouse)		MalK (human)	MalK (mouse)		GluK (human)		GluK (mouse)	
ATP5C1			5		Not determined						
			15								
			19								
			22								
			25	[4]							
			29	[1, 5]							
			31	[4]							
			39	[1, 2, 5]		39	[7]				
			43								
			46	[2]							
			49	[1, 2, 4, 5]							
			55	[2, 4, 5]							
			59								
	64	[4]	64	[2]							
			79	[2]							
			83	[2, 5]							
			88	[2]							
			89	[2]							
			90	[2]							
			91	[3, 4]							
			32	[1, 3]							
			34	[3]							
	35	[4]	35	[1-5]							
			126	[2, 5]							
			130	[3, 4]							
			136	[2]							
			138	[1-3, 5]		138	[7]				
	154	[4]	154	[1-5]		154	[7]			154	[9]
			159	[2]							
			191	[2]							
	197	[4]	197	[4]							
			246	[3, 4]							
			262	[2, 5]							
			270	[2-4]							

ATP synthase subunit	SuccK (human)		SuccK (mouse)		MalK (human)	MalK (mouse)		GluK (human)		GluK (mouse)	
ATP5D			136	[1-6]	Not determined	136	[7]			136	[9]
	165	[4]	165	[1-6]							
ATP5E	21	[4]	21	[1-6]	Not determined	21	[7]			21	[9]
	28	[4]	28	[2-5]							
	32	[4]	32	[2, 4, 5]							
			37	[2-4]		37	[7]			37	[9]
	44	[4]	44	[1-4]		44	[7]			44	[9]
			47	[2]							
			50	[2]							
ATP5F1			53	[1, 2]	Not determined						
			121	[3]							
			126	[2]							
	131	[4]	131	[2-5]		131	[7]				
			139	[2, 3, 5]							
			144	[2-5]							
			154	[1-3]							
	162	[4]	162	[1-3, 5, 6]		162	[7]				
			188	[2, 3]							
			191	[2, 3, 6]							
			194	[2]							
			221	[1, 2]							
			225	[1-5]		225	[7, 10]				
	233	[4]	233	[1-6, 8]		233	[7]				
			238	[2, 3]							
	244	[4]	244	[1-3, 6]							
			248	[2]							
			249	[2, 3]							
ATP5H			25	[1, 5, 6]	Not determined						
	32	[4]	32	[4, 5, 8]							
			48	[1, 3, 5, 6, 8]							
	58	[4]									
	63	[4]	63	[1, 3, 5, 6, 8]							

ATP synthase subunit	SuccK (human)		SuccK (mouse)		MalK (human)	MalK (mouse)		GluK (human)		GluK (mouse)	
**ATP5H**	72	[4]	72	[1, 3-6]	Not determined	72	[7]				
			73	[1, 6]							
	78	[4]	78	[1, 3, 4, 8]		78	[7]			78	[9]
	85	[4]	85	[1, 3-5]		85	[7]				
	95	[4]	95	[1, 3-6]		95	[7]				
	99	[4]	99	[1, 3, 5, 6]							
	109	[4]									
	37	[4]	37	[1, 3-6]		37	[7, 10]				
			121	[6]							
	144	[4]	144	[1, 4, 5]							
			148	[1, 4]							
			149	[1, 3-6]		149	[7]				
**ATP5I**			3	[4]	Not determined						
			12	[3]							
			24	[4]							
			28	[3]							
	34	[4]	34	[3]							
			35	[4]							
			48	[3]							
			49	[3]							
			54	[4]							
			55	[3, 4]							
			61	[4]							
			66	[4]							
**ATP5J**			34	[1]	Not determined						
	41	[4]	41	[1, 5, 6]							
	46	[4]	46	[1, 3-6]							
	51	[4]	51	[3]							
	79	[4]	79	[1, 3]		79	[7]				
			84	[1, 3, 4, 8]							

ATP synthase subunit	SuccK (human)		SuccK (mouse)		MalK (human)	MalK (mouse)		GluK (human)		GluK (mouse)	
ATP5J			94	[1, 4-6, 8]	Not determined					94	[9]
	99	[4]	99	[1, 3-6, 8]						99	[9]
			105	[1, 3, 5]							
ATP5J2			8	[2]	Not determined	8	[7]				
			3	[2, 3]							
			16	[1-3]							
			48	[2]							
ATP5L			3	[1]	Not determined						
			24	[3, 4]							
			35	[1, 6]							
			54	[1, 3-6, 8]							
			55	[3, 5, 6]		55	[7]				
			61	[1, 3-5]		61	[7]				
	66	[4]	66	[1, 3-5]							
ATP5O			24	[4]	Not determined						
			26	[1, 2]							
			31	[4]							
			34	[4]							
			51	[2]							
			53	[2, 3]							
			54	[1-3, 5, 6]		54	[7]			54	[9]
	60	[4]	60	[1-3, 5, 6, 8]		60	[7, 10]			60	[9]
	70	[4]	70	[1-6]		70	[7, 10]			70	[9]
			73	[1-3, 5]						73	[9]
	84	[4]	84	[1, 3, 5, 6, 8]							
			90	[1-4]		90	[7, 10]			90	[9]
			97	[1, 2, 4, 5, 8]		97	[7, 10]			97	[9]
	98	[4]									
			100	[1-4]							
			158	[1-6]							

ATP synthase subunit	SuccK (human)		SuccK (mouse)		MalK (human)	MalK (mouse)		GluK (human)		GluK (mouse)	
ATP5O	162	[4]	162	[1-6, 8]	Not determined	162	[10]			162	[9]
			172	[1-3, 5, 6, 8]							
	176	[4]	176	[1-6]							
	192	[4]	192	[1-6]							
ATP5O	199	[4]	199	[1-6, 8]	Not determined					199	[9]
			201	[2]							
			204	[2]							
			207	[2]							
ATP8			46	[3, 4]	Not determined	46	[7]				
			48	[4]		48	[7]				
			54	[4]							
USMG5			17	[1, 3]	Not determined						

S1 Table References

1. Rardin MJ, He W, Nishida Y, Newman JC, Carrico C, Danielson SR, et al. SIRT5 regulates the mitochondrial lysine succinylome and metabolic networks. Cell metabolism. 2013;18(6):920-33.
2. Hershberger KA, Abraham DM, Martin AS, Mao L, Liu J, Gu H, et al. Sirtuin 5 is required for mouse survival in response to cardiac pressure overload. The Journal of biological chemistry. 2017;292(48):19767-81.
3. Hershberger KA, Abraham DM, Liu J, Locasale JW, Grimsrud PA, Hirschey MD. Ablation of Sirtuin5 in the postnatal mouse heart results in protein succinylation and normal survival in response to chronic pressure overload. J Biol Chem. 2018.
4. Park J, Chen Y, Tishkoff DX, Peng C, Tan M, Dai L, et al. SIRT5-mediated lysine desuccinylation impacts diverse metabolic pathways. Molecular cell. 2013;50(6):919-30.
5. Weinert BT, Scholz C, Wagner SA, Iesmantavicius V, Su D, Daniel JA, et al. Lysine succinylation is a frequently occurring modification in prokaryotes and eukaryotes and extensively overlaps with acetylation. Cell reports. 2013;4(4):842-51.
6. Boylston JA, Sun J, Chen Y, Gucek M, Sack MN, Murphy E. Characterization of the cardiac succinylome and its role in ischemia-reperfusion injury. Journal of molecular and cellular cardiology. 2015;88:73-81.
7. Colak G, Pougovkina O, Dai L, Tan M, Te Brinke H, Huang H, et al. Proteomic and Biochemical Studies of Lysine Malonylation Suggest Its Malonic Aciduria-associated Regulatory Role in Mitochondrial Function and Fatty Acid Oxidation. Molecular & cellular proteomics : MCP. 2015;14(11):3056-71.
8. Sadhukhan S, Liu X, Ryu D, Nelson OD, Stupinski JA, Li Z, et al. Metabolomics-assisted proteomics identifies

succinylation and SIRT5 as important regulators of cardiac function. Proceedings of the National Academy of Sciences of the United States of America. 2016;113(16):4320-5.
9. Tan M, Peng C, Anderson KA, Chhoy P, Xie Z, Dai L, et al. Lysine glutarylation is a protein posttranslational modification regulated by SIRT5. Cell metabolism. 2014;19(4):605-17.
10. Nishida Y, Rardin MJ, Carrico C, He W, Sahu AK, Gut P, et al. SIRT5 Regulates both Cytosolic and Mitochondrial Protein Malonylation with Glycolysis as a Major Target. Molecular cell. 2015;59(2):321-32.
