# Supplementary material for: SIRT5 deficiency suppresses mitochondrial ATP production and promotes AMPK activation in response to energy stress
Source: PLoS One. 2019 Feb 13;14(2):e0211796. doi: 10.1371/journal.pone.0211796 (PMC6373945; doi:10.1371/journal.pone.0211796)
Supplement: S1 Dataset — (ZIP) [file pone.0211796.s017.zip › Original data.pdf]

Original data for WB

Fig 1A

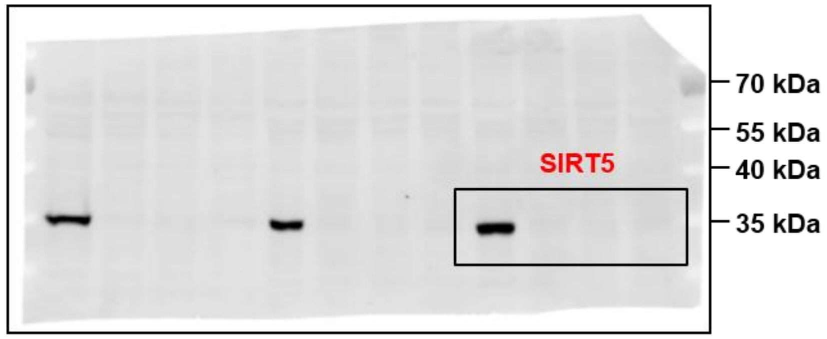

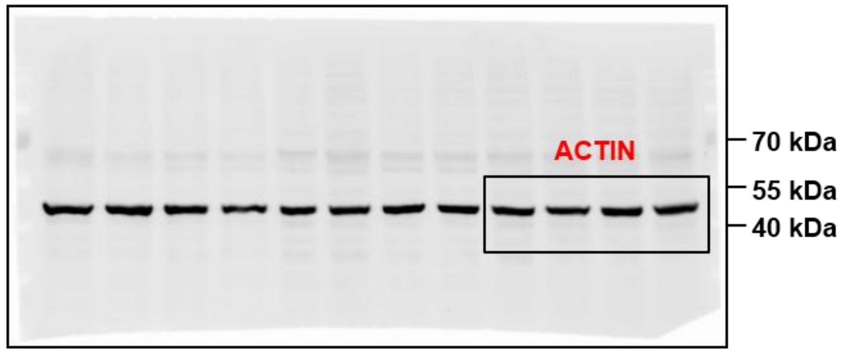

Fig 1H

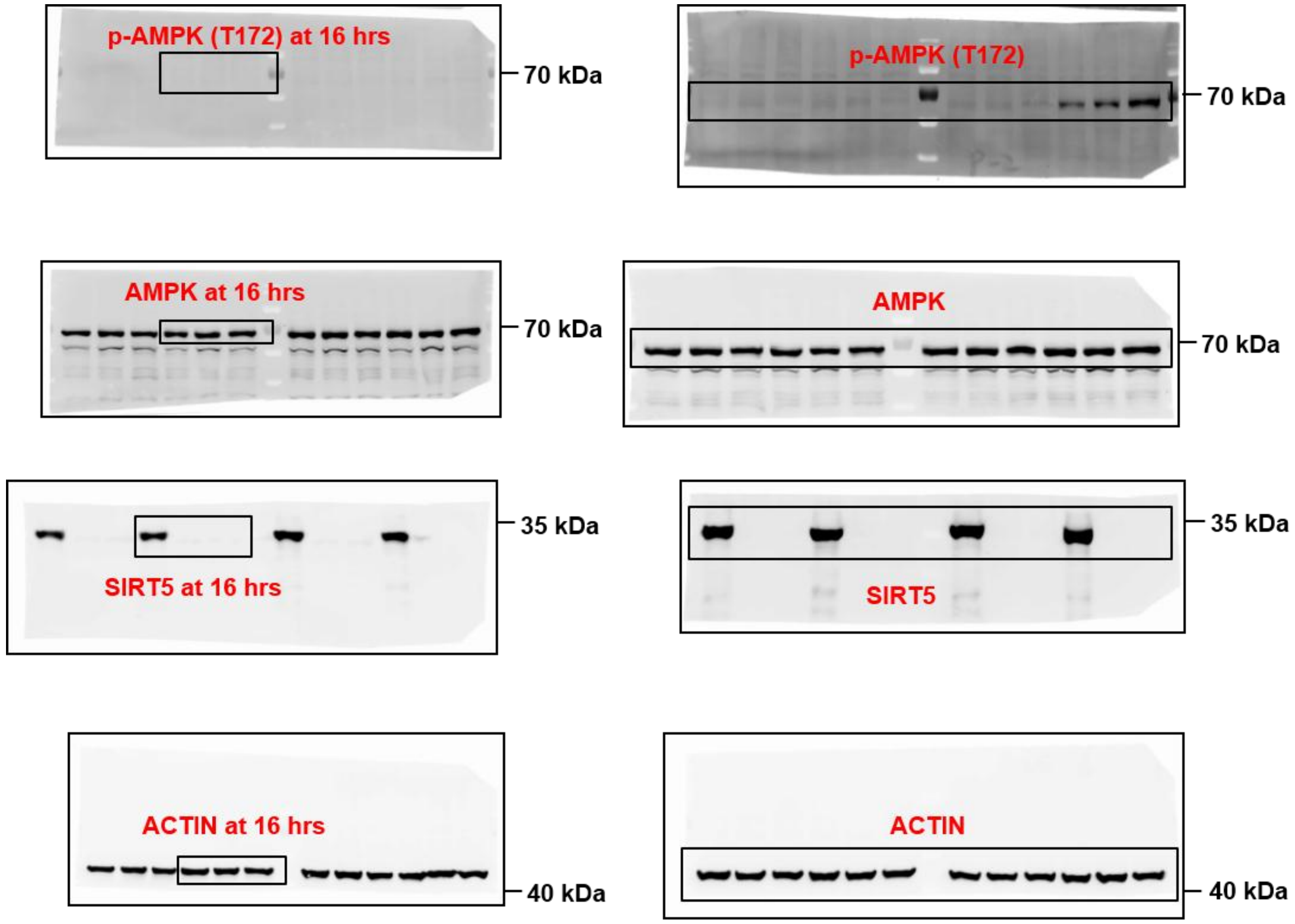

p-AMPK (T172) at 16 hrs
70 kDa
p-AMPK (T172)
70 kDa
AMPK at 16 hrs
70 kDa
AMPK
70 kDa
SIRT5 at 16 hrs
35 kDa
SIRT5
35 kDa
ACTIN at 16 hrs
40 kDa
ACTIN
40 kDa

Fig 1I

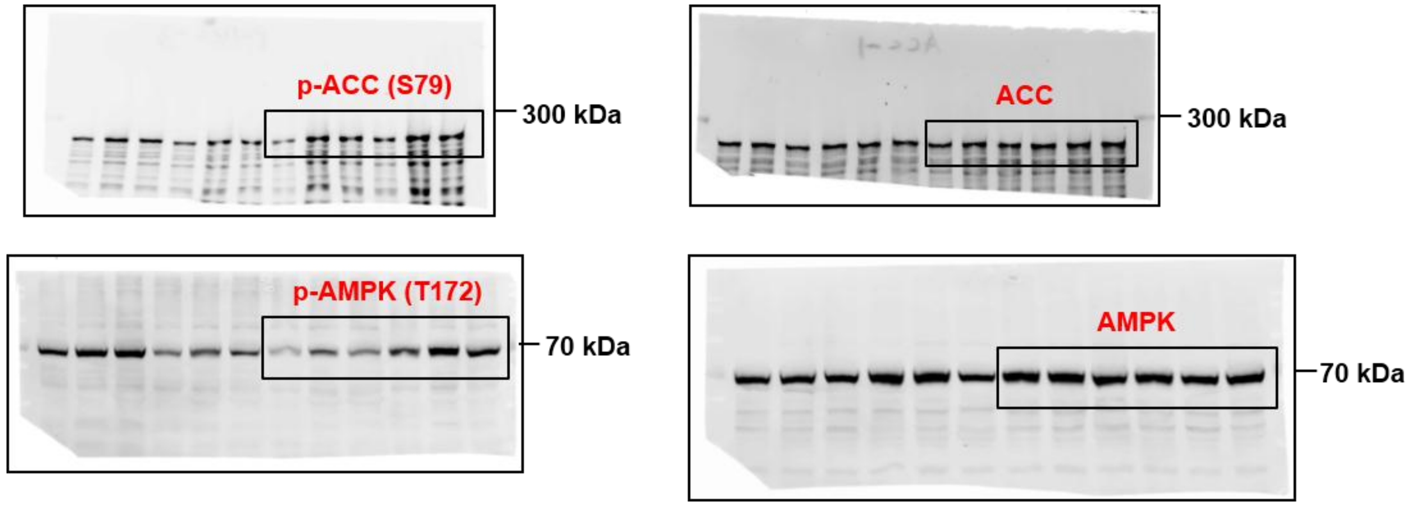
p-ACC (S79)
300 kDa
ACC
300 kDa
p-AMPK (T172)
70 kDa
AMPK
70 kDa

Fig 2F

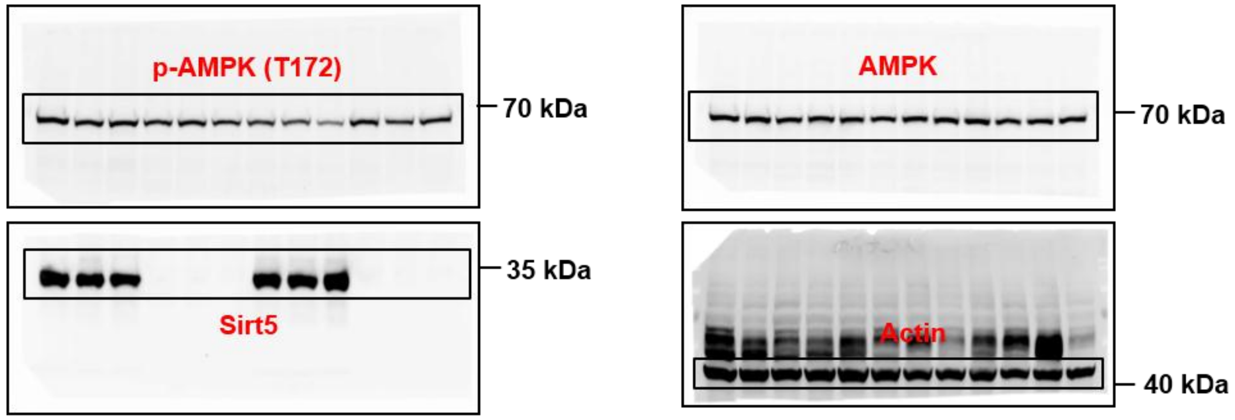
p-AMPK (T172)
70 kDa
AMPK
70 kDa
35 kDa
Sirt5
Actin
40 kDa

Fig 3A-3C

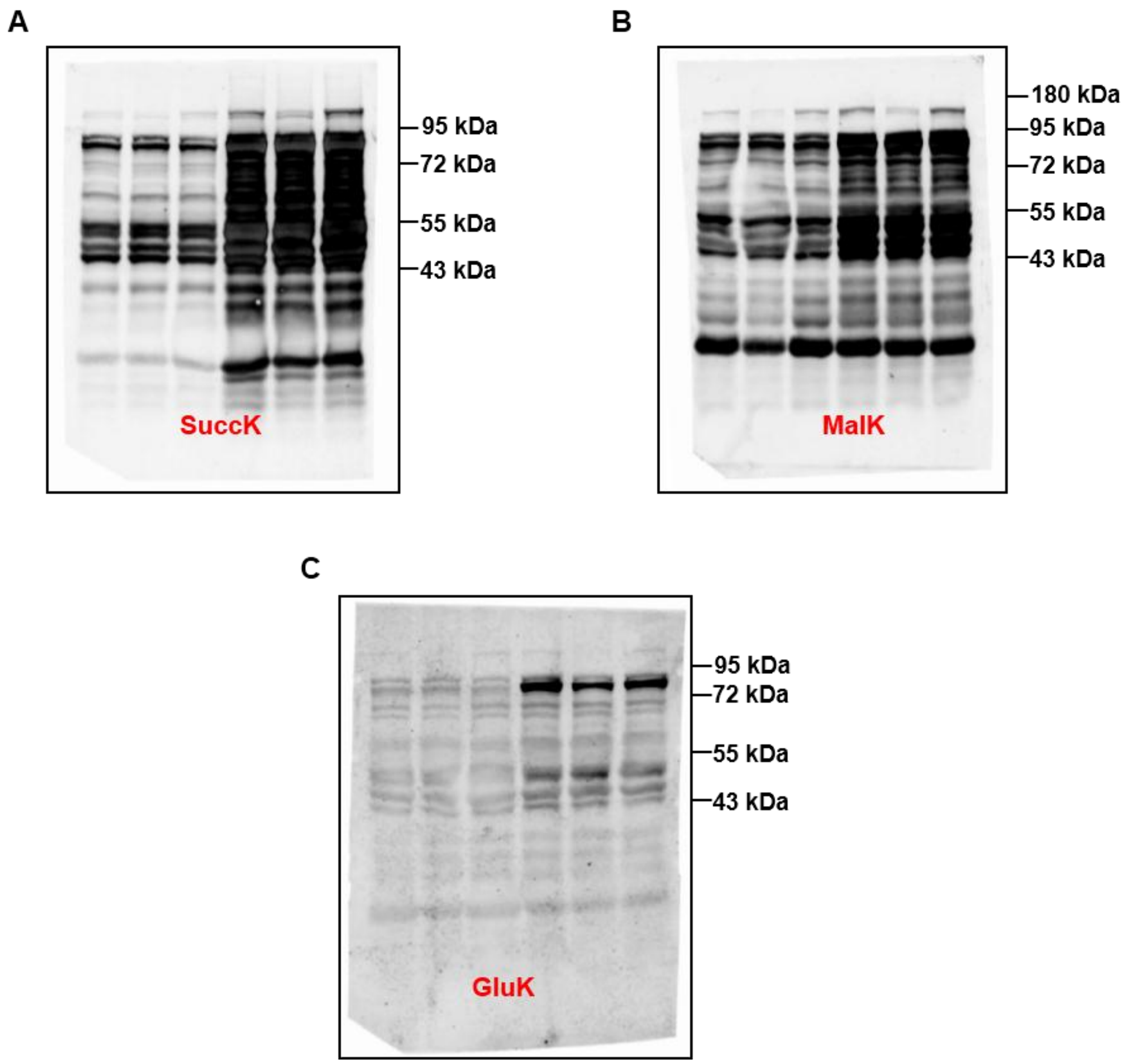
A
95 kDa
72 kDa
55 kDa
43 kDa
SuccK
B
180 kDa
95 kDa
72 kDa
55 kDa
43 kDa
MalK
C
95 kDa
72 kDa
55 kDa
43 kDa
GluK

Fig 6E

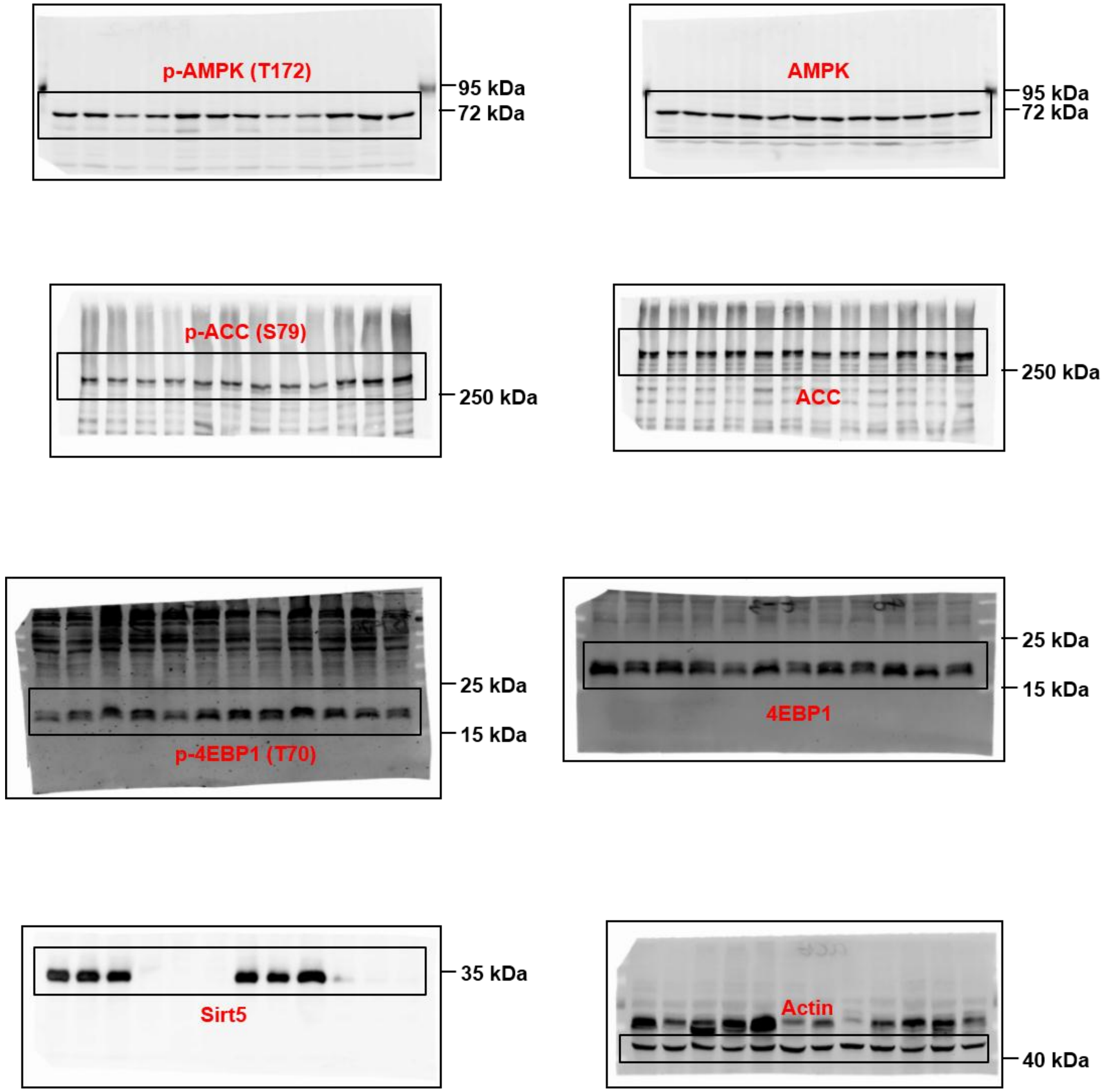
p-AMPK (T172)
95 kDa
72 kDa
AMPK
95 kDa
72 kDa
p-ACC (S79)
250 kDa
ACC
250 kDa
25 kDa
15 kDa
p-4EBP1 (T70)
25 kDa
15 kDa
4EBP1
35 kDa
Sirt5
Actin
40 kDa

S9A Fig

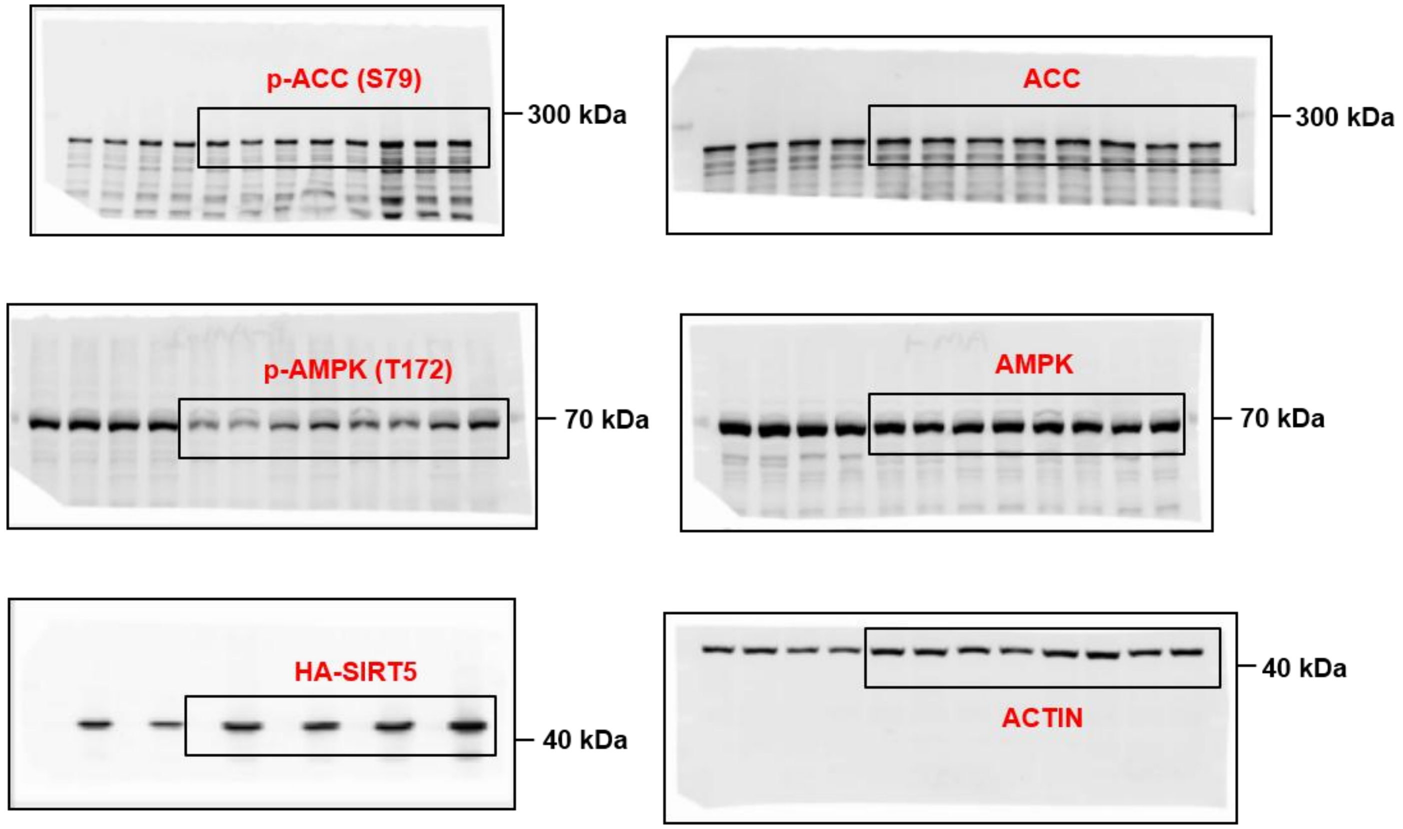
p-ACC (S79)
300 kDa
ACC
300 kDa
p-AMPK (T172)
70 kDa
AMPK
70 kDa
HA-SIRT5
40 kDa
ACTIN
40 kDa

S9B Fig

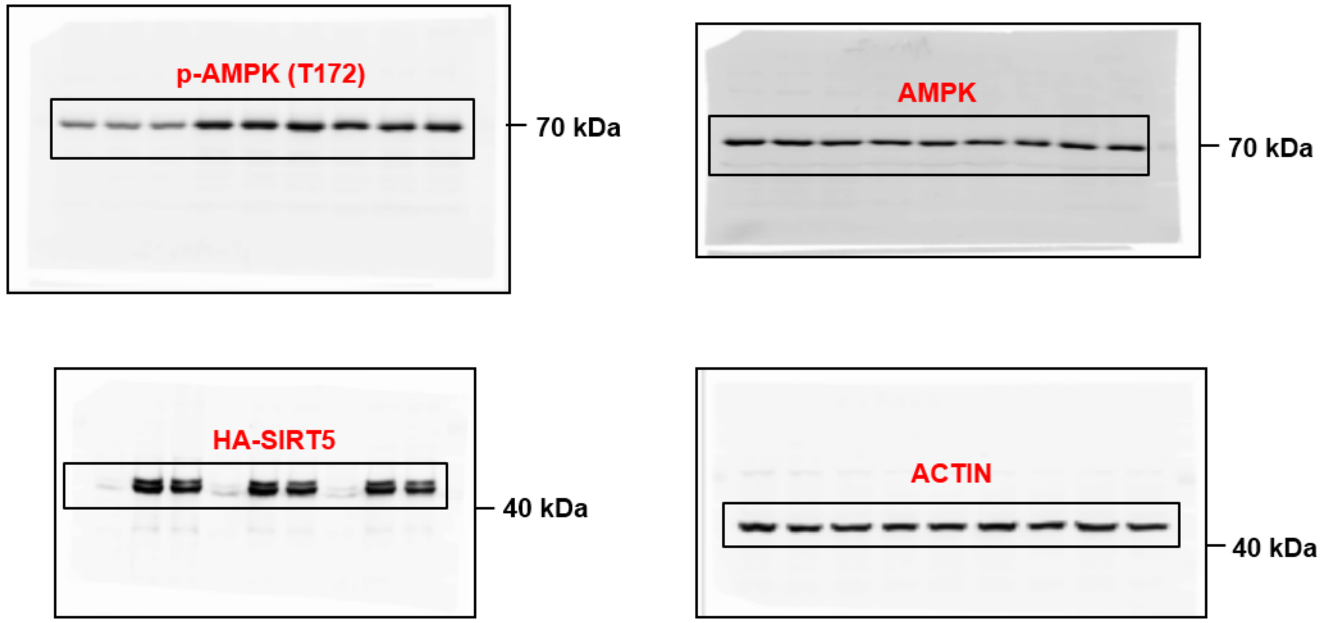

S10C Fig

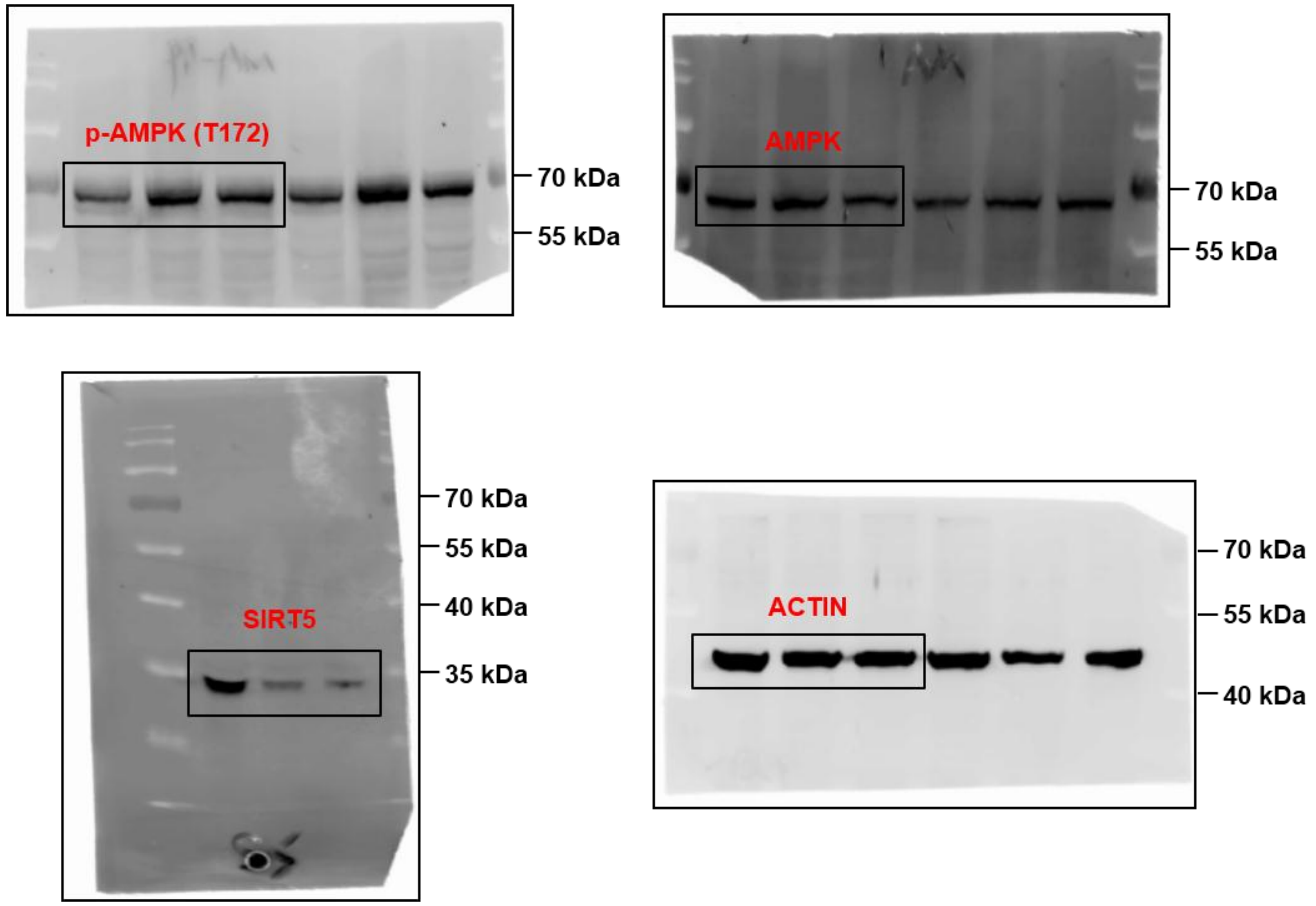

p-AMPK (T172)
70 kDa
55 kDa
AMPK
70 kDa
55 kDa
SIRT5
70 kDa
55 kDa
40 kDa
35 kDa
ACTIN
70 kDa
55 kDa
40 kDa

S10F Fig

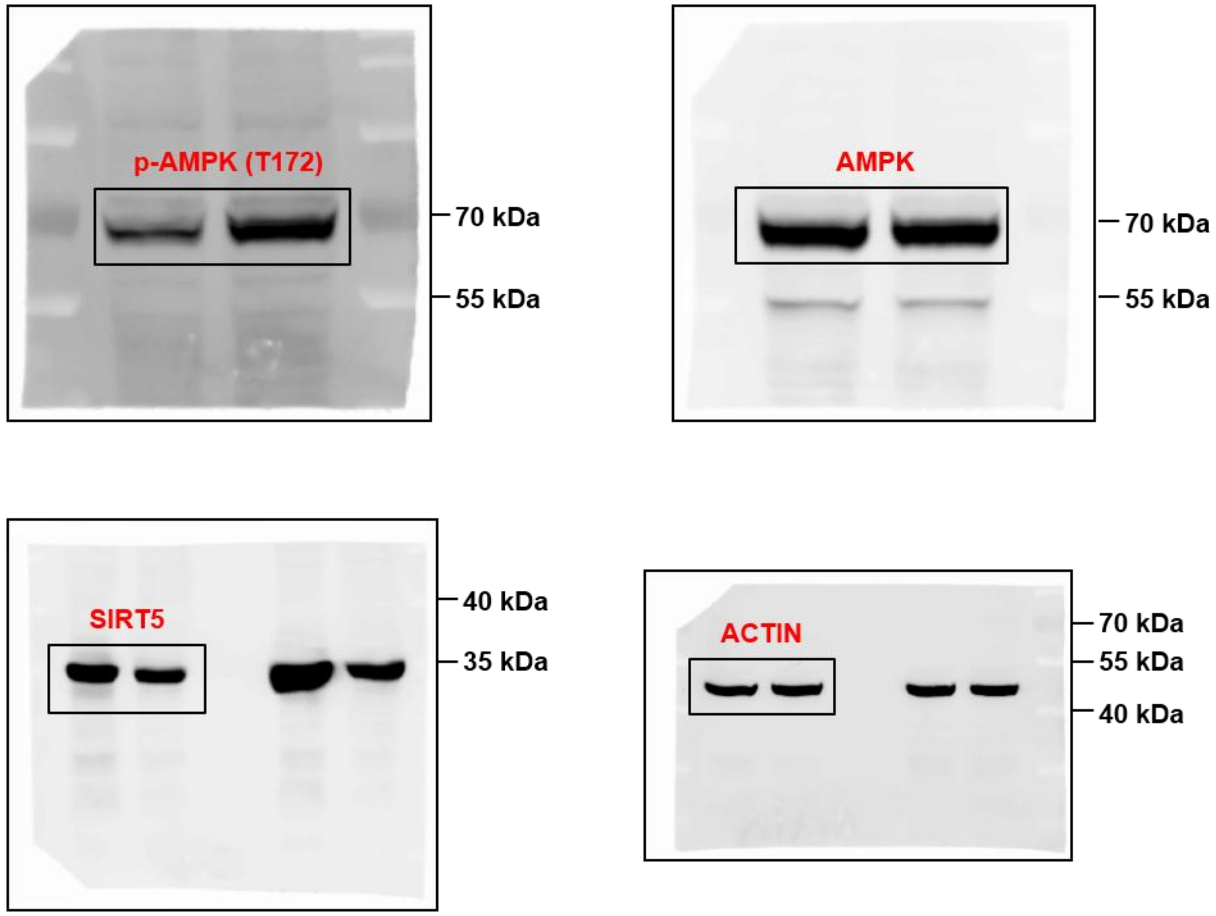
p-AMPK (T172)
70 kDa
55 kDa
AMPK
70 kDa
55 kDa
SIRT5
40 kDa
35 kDa
ACTIN
70 kDa
55 kDa
40 kDa

S11 Fig

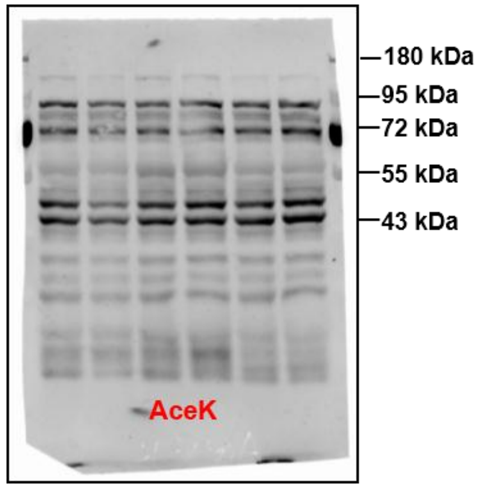

S12A Fig

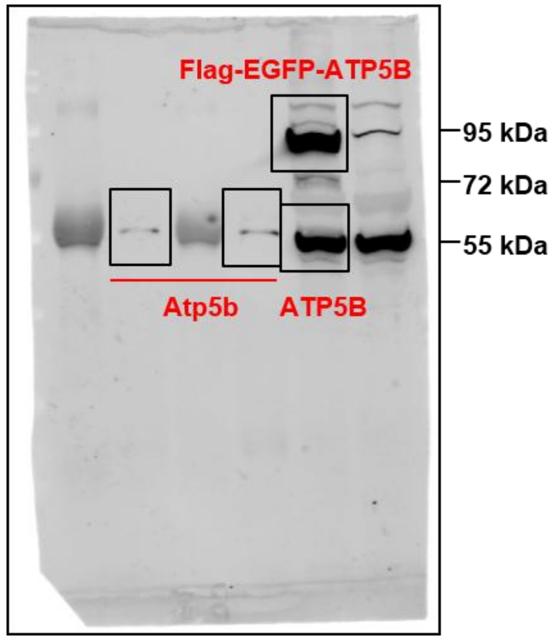

S12B Fig

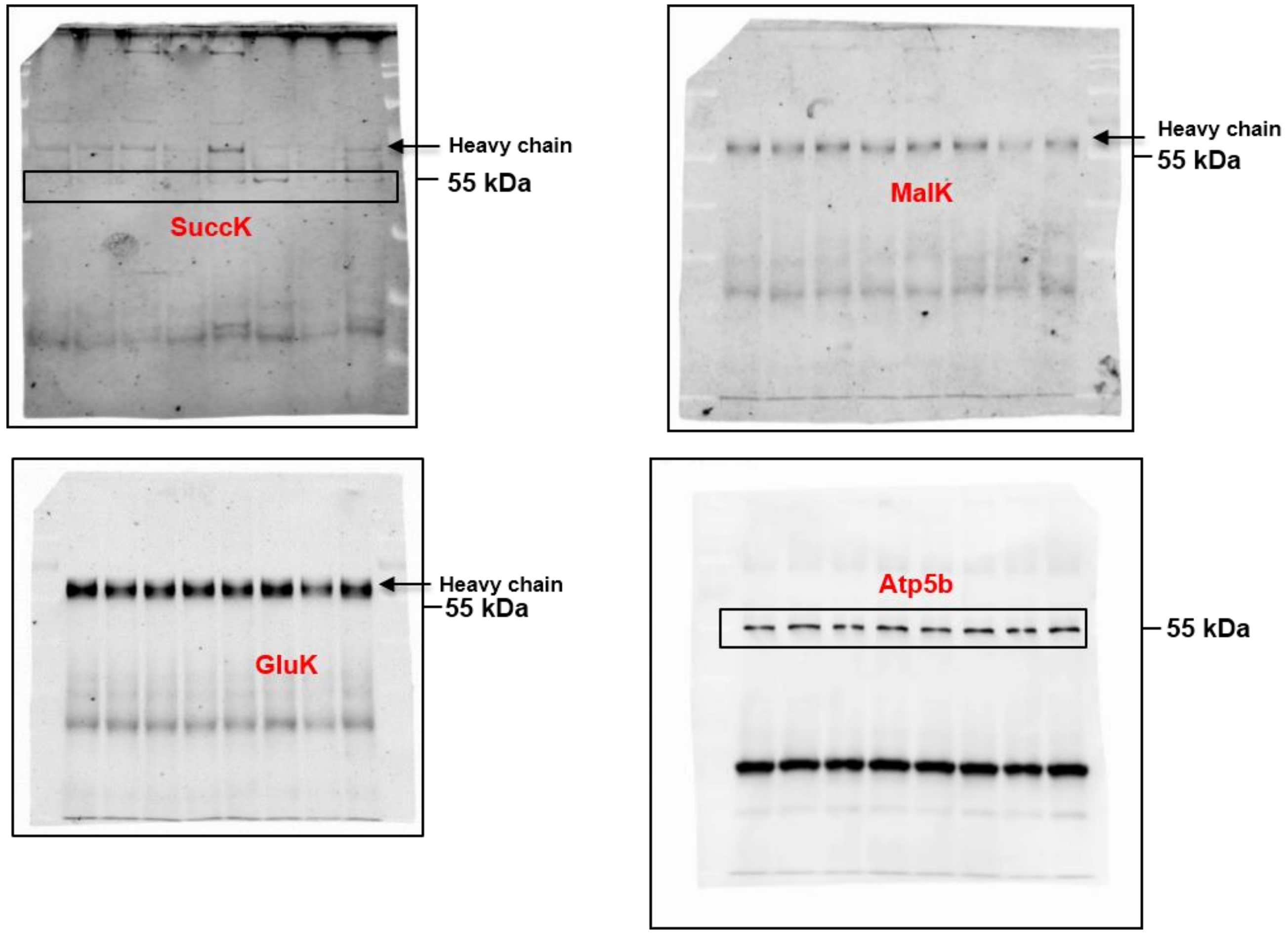
Heavy chain
55 kDa
SuccK
Heavy chain
55 kDa
MalK
Heavy chain
55 kDa
GluK
Atp5b
55 kDa

S14A Fig

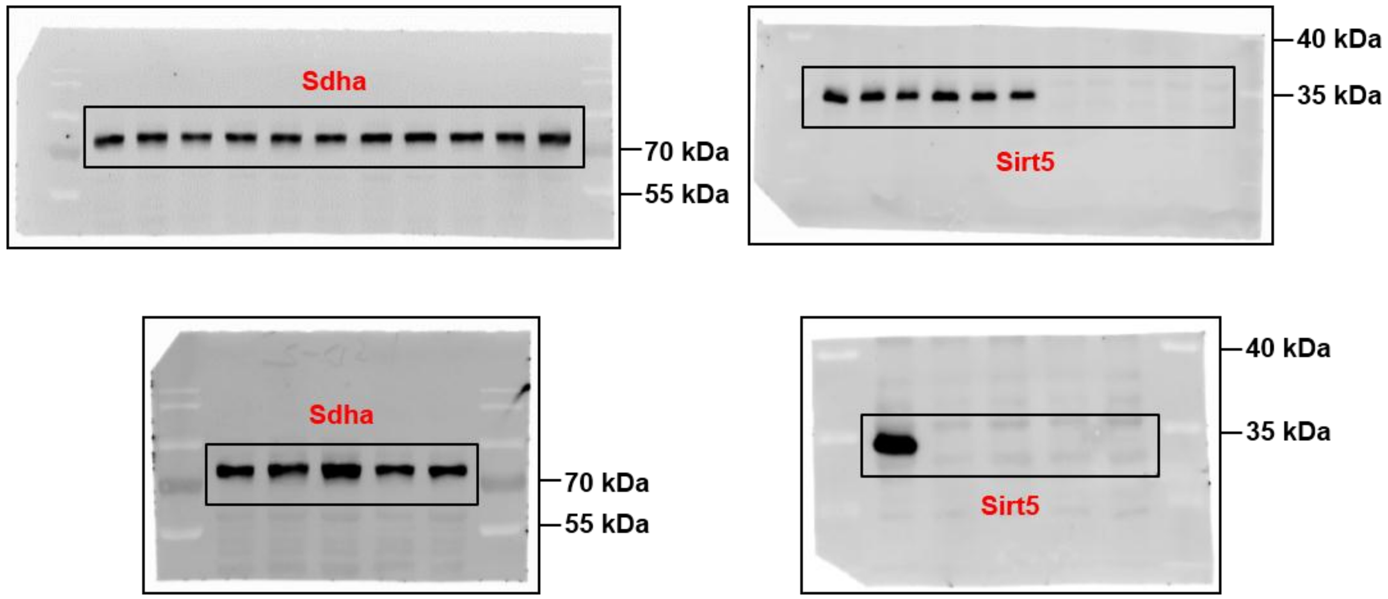
Sdha
70 kDa
55 kDa
40 kDa
35 kDa
Sirt5
Sdha
70 kDa
55 kDa
40 kDa
35 kDa
Sirt5

Original data for Graphs and Table 1

Fig 1:

	Cell line	Normalized NADH level in the cytosol			T.TEST
Fig 1B	WT	1.100205	0.9018481	0.9979466	
	SIRT5 KO-#1	1.327515	1.288706	1.023203	**0.128423**
	SIRT5 KO-#2	1.135318	1.293018	1.111294	**0.089982**

	Cell line	Normalized NADH level in the mitochondria			T.TEST
Fig 1C	WT	0.9115016	1.08115	1.007348	
	SIRT5 KO-#1	2.180511	2.542811	2.569649	**0.000445**
	SIRT5 KO-#2	2.370288	2.611821	2.328115	**0.000142**

	Culture time	Metabolite / Cell line	ATP abundance												T.TEST
Fig 1D	16 hrs	WT	12726280	13892974	13956085	12934126	12883804	11785556	15447428	14527494	18144528	17255454	17699012	21603764	
		SIRT5 KO-#1	4053714.75	4189181	4361790.5	5610720.5	5752292.5	5945492.5	4082825.5	3911083.5	4072938.5	1079641.9	952016.81	891832.31	**6.98411E-11**
		SIRT5 KO-#2	5619281.5	6020173	6356737.5	5043547.5	5301271.5	5631527.5	6151951	6292619	6910410	5566998	5426790	5194199	**1.75025E-10**
	48 hrs	WT	6671836.5	5880691	5524043.5	4691517.5	4334812.5	4204459	2557782	2384388.3	1840321.8	1760873.1	1744889.3		
		SIRT5 KO-#1	355271.063	314455.72	307417.66	429835.53	435962.25	418387.31	925264.88	1003526.7	983340.5	676740.81	687808.63	700843.88	**5.33792E-06**
		SIRT5 KO-#2	2552557.5	2361899.8	2201747.3	2463930.8	2398579.25	2423892.8	1150488.9	1139627.3	1114624.5	2007298.1	2087876.6	1999293.8	**0.003559023**
	72 hrs	WT	1079042.5	1019193.9	1024786.7	2368572	2276915.75	2301399.5	3126291.8	3173656	3198615.3				
		SIRT5 KO-#1	292633.313	262056.66	244431.88	322563.38	319178.031	319673.69	218163.73	199196.52	217345.16				**1.35364E-05**
		SIRT5 KO-#2	1072950.88	974535.81	923495.38	956359.44	901350.688	907607.13	654960.13	633306	601638.13				**0.000646597**

	Culture time	Metabolite / Cell line	ADP abundance												T.TEST
Fig 1E	16 hrs	WT	8959006	8360030.5	8067932.5	9865393	9971073	9818229	15233257	11882004	12140565	17745984	18613902	17631186	
		SIRT5 KO-#1	4946386	4480780	4313378.5	3452019.5	3277366	3388225.5	2679043.3	2637555.5	2641160.5	2191863	2260097.3	2173146.3	**7.78346E-08**
		SIRT5 KO-#2	3134089.75	2979401.8	2927762.8	3398599	3406871	3395749.3	3619213.3	3450279.5	3499468.8	4268952	4410116	4260398.5	**1.0728E-07**
	48 hrs	WT	7573722	7109676.5	6933297	7904252.5	7731365.5	7645869.5	6603400.5	5736513	5352572.5	5154798	5006016	5066266	
		SIRT5 KO-#1	1418871.13	1417094.9	1387903	1707376.1	1667401	1670745.5	1974725.3	2000402.9	1938514.5	1773375.9	1756591.9	1787537.9	**1.02088E-12**
		SIRT5 KO-#2	2787429	2823545.5	2729106.5	3726018.5	3765340	3797813.3	2819482.8	2934548.8	2892757.5	4818993.5	4715894	4723938.5	**2.07283E-07**
	72 hrs	WT	6360605.5	6047066	5949023.5	9471136	9291447	9074070	8341491	8424528	8417698				
		SIRT5 KO-#1	2522786.75	2411847.5	2457451.3	2120968.3	2171544.25	2349366.3	2035082.5	1928130.3	2148979.5				**2.30453E-09**
		SIRT5 KO-#2	3052151	2880249.5	2818647.5	3688691.3	3528046.75	3478530.5	3157020	3247582.5	3131722.8				**3.92626E-08**

	Culture time	Metabolite / Cell line	AMP abundance												T.TEST
Fig 1F	16 hrs	WT	149558.656	148227.84	149804.84	185382.08	184998.25	180583.38	207159.28	179301.77	175329.33	250767.59	247641.06	253676.83	
		SIRT5 KO-#1	155911.297	151146.5	155445.16	141528.34	136914.688	132036.27	136597.73	132715.39	130196.89	153392.84	155398.69	157472.95	**0.000482854**
		SIRT5 KO-#2	86674.609	96326.672	91943.336	131859.05	132215.766	128114.48	107881.06	103357.02	103568.84	171841.41	165676.88	165038.23	**7.86851E-05**
	48 hrs	WT	307423.688	299782.09	301445.41	240167.72	228192.375	239468.83	163488.88	147965.39	137828.28	139343.02	142650.83	140257.33	
		SIRT5 KO-#1	183753.969	185280.08	172621.09	206227.81	225492.406	208773.84	238650.33	232156.84	234900.39	103385.46	97498.031	101071.41	**0.243981993**
		SIRT5 KO-#2	124595.547	121583.95	115831.85	174240.36	170522.672	169114.3	183283.06	185894.88	190714.94	219057.05	214659.77	216016.8	**0.098131571**
	72 hrs	WT	496109.406	510030.97	505130.75	533011.19	549009.25	514081.69	494174.69	471009.94	481987.81				
		SIRT5 KO-#1	240914.609	238458.53	230377.44	253242.94	248659.766	257294.08	216826.42	216303.61	207788.45				**8.18642E-15**
		SIRT5 KO-#2	293729.156	291021.56	278038.97	391552.31	390535.563	388984.09	358920.75	356323.47	347429				**6.12088E-08**

	Culture time	Metabolite / Cell line	AMP/ATP ratio												T.TEST
Fig 1G	16 hrs	WT	0.011752	0.0106693	0.010734	0.014332787	0.014359	0.0153224	0.0134106	0.0123422	0.0096629	0.0145327	0.0139918	0.0117423	
		SIRT5 KO-#1	0.0384613	0.0360802	0.0356379	0.025224629	0.0238018	0.0222078	0.0334567	0.0339332	0.0319663	0.1420775	0.163231	0.1765724	**0.007051283**
		SIRT5 KO-#2	0.0154245	0.0160006	0.0144639	0.026144107	0.0249404	0.0227495	0.0175361	0.0164251	0.0149874	0.0308679	0.0305294	0.0317736	**0.000193524**
	48 hrs	WT	0.0460778	0.0509774	0.0545697	0.051191905	0.0526418	0.0569559	0.0639182	0.0620559	0.0748936	0.0791329	0.0817535		
		SIRT5 KO-#1	0.5172219	0.5892088	0.5615198	0.479783075	0.5172292	0.4989966	0.2579265	0.231341	0.23888	0.1527697	0.1417517	0.1442139	**1.82239E-05**
		SIRT5 KO-#2	0.048812	0.0514772	0.0526091	0.070716419	0.0710932	0.0697697	0.1593089	0.163119	0.1711024	0.1091303	0.1028125	0.1080466	**0.016250179**
	72 hrs	WT	0.4597682	0.5004259	0.4929131	0.225034826	0.2411197	0.2233779	0.1580706	0.1484124	0.1506864				
		SIRT5 KO-#1	0.8232645	0.9099503	0.9425016	0.78509514	0.7790629	0.8048647	0.9938701	1.0858805	0.9560298				**3.22983E-08**
		SIRT5 KO-#2	0.2737583	0.2986258	0.3010724	0.409419615	0.4332782	0.428582	0.548004	0.5626403	0.5774717				**0.047743042**

Fig 2:

Fig 2A	Mouse heart	ATP (nmol/g)									T.TEST
	WT	401.2655685	441.69345	409.04982	490.91	528.0735235	478.35476	517.27601	508.23624	517.52712	
	Sirt5 KO	377.1595018	354.56006	423.11169	315.88992	312.8766573	314.13218	404.78104	422.35838	432.40257	**0.000384**

Fig 2B	Mouse heart mitochondria	ATP abundance										
	WT	124064.66	129543.02	124811.47	134342.94	136566.55	129352.79	123401.82	130208.80	128273.90	121816.77	120820.52
	Sirt5 KO	105859.56	111615.93	102699.09	95833.84	95821.90	97973.43	122231.89	120616.98	118255.48	114607.79	109829.30

Fig 2B	Mouse heart mitochondria	ATP abundance										T.TEST
	WT	120172.54	103145.54	102771.66	99570.96	100174.05	106200.22	104060.71				
	Sirt5 KO	108242.95	106512.64	107568.21	105924.99	75883.93	75933.31	75180.07	96952.93	96031.82	96779.55	**0.000289223**

Fig 2C	Mouse heart mitochondria	ADP abundance										
	WT	293616.28	294167.81	285414.66	224643.11	225117.69	230110.28	237212.06	242748.48	242554.44	207777.34	212891.03
	Sirt5 KO	294705.06	311280.09	302135.09	186347.14	191841.66	195697.73	245948.52	245734.63	257572.63	189128	195586.42

Fig 2C	Mouse heart mitochondria	ADP abundance										T.TEST
	WT	226255.08	195664.17	217755.13	191364.44	192808.75	184620.56	188867.53				
	Sirt5 KO	187752.33	228028.39	227636.19	227096.22	194903.11	193053.67	189792.75	212461.72	198234.58	214638.28	**0.733849858**

Fig 2D	Mouse heart mitochondria	AMP abundance										
	WT	28063.45	28408.83	31474.86	18233.07	17669.96	19238.02	24112.28	23822.93	22773.54	24819.25	26158.63
	Sirt5 KO	47022.73	45732.06	49096.29	36472.48	37459.69	35926.58	25834.37	27189.21	28198.61	17487.23	19201.81

Fig 2D	Mouse heart mitochondria	AMP abundance										T.TEST
	WT	27596.74	23523.72	24573.78	25494.4	19798.69	19425.87	20756.76				
	Sirt5 KO	18949.25	30394.03	33441.83	31958.03	15996.83	16889.88	19381.85	33228.93	33281.57	33623.14	**0.011439601**

Fig 2E	Mouse heart mitochondria	AMP/ATP ratio										
	WT	0.226200193	0.2193004	0.2521792	0.1357203	0.129387174	0.1487252	0.1953965	0.1829594	0.1775384	0.2037425	0.2165082
	Sirt5 KO	0.444199182	0.409727	0.4780596	0.3805804	0.390930361	0.3666972	0.2113554	0.2254178	0.238455	0.1525833	0.1748332

Fig 2E	Mouse heart mitochondria	AMP/ATP ratio										T.TEST
	WT	0.2296426	0.2280634	0.2391105	0.2560425	0.1976429	0.1829174	0.1994678				
	Sirt5 KO	0.1750622	0.2853561	0.3108895	0.3017043	0.2108066	0.2224304	0.2578057	0.3427326	0.3465681	0.3474199	**0.000193817**

		Mouse heart	p-AMPK/AMPK ratio			**T.TEST**
Fig 2F	Fed	WT	1	0.78	1.04	
		Sirt5 KO	0.75	0.89	0.81	**0.244323529**
	Fasted	WT	0.67	0.59	0.39	**0.028285497**
		Sirt5 KO	1	0.75	0.9	**0.03929881**

Fig 3:

Fig 3E	Mouse heart mitochondria	Relative citrate synthase activity												
	WT	1.2624209	1.468217	1.5035402	0.514149	0.5946074	0.7113702	0.7211822	0.7928099	1.4758959	0.5798894	0.6004946	0.8713059	0.6456298
	Sirt5 KO	1.4345149	1.6061672	1.4429532	1.506535	1.7347622	1.5802489	0.7506182	0.4793864	0.5298482	1.2857649	1.3577359	1.836807	0.8427109

Fig 3E	Mouse heart mitochondria	Relative citrate synthase activity												
	WT	0.6995958	1.4344295	1.38682	1.2153801	1.5222622								
	Sirt5 KO	0.8242083	1.6267268	1.4788651	0.5455474	0.577927	1.2469094	0.8931726	0.6845975	0.6105869	0.640864	1.5961676	1.6150557	1.6935518

Fig 3E	Mouse heart mitochondria	Relative citrate synthase activity	**T.TEST**
	WT		
	Sirt5 KO	1.6713456	**0.16209064**

Fig 3F	Mouse heart mitochondria	Relative ATP synthase activity												
	WT	1.606801772	1.5872829	0.9215513	1.9399687	2.4926459	1.0513189	1.133988	1.5864391	0.6608614	1.8588349	1.7351255	1.3047176	1.2753461
	Sirt5 KO	1.128210068	1.0140991	1.2289219	1.7467317	1.6858737	0.6434244	0.9007416	1.412867	1.3690364	0.9254427	1.0550049	0.815744	0.9477152

Fig 3F	Mouse heart mitochondria	Relative ATP synthase activity												
	WT	1.263769	0.8661474	1.5109157	1.3677991	1.0635454								
	Sirt5 KO	0.9168242	0.653854	0.8278775	1.2842116	1.4862657	1.0013712	0.9270136	0.9679559	1.5386583	0.9160349	0.8609651	0.9138419	1.1393992

Fig 3F	Mouse heart mitochondria	Relative ATP synthase activity	**T.TEST**
	WT		
	Sirt5 KO	1.2021184	**0.00723985**

Fig 4:

	TAC operation	Mouse heart	HW/BW ratio (mg/g)										One-way ANOVA
	Sham	WT	6.392515	6.08365	5.678694	7.112069	5.023119	5.652174					**n.s.**
Fig 4A	Sham	*Sirt5* KO	5.840497	5.142586	5.480072	5.258488	6.928072	6.779752	6.587802				**P<0.001**
	TAC	WT	12.99663	10.91983	8.879944	6.876585	11.8396	6.904762	9.949875	10.77875	10.14772		**P<0.001**
	TAC	*Sirt5* KO	7.279438	10.68884	7.501237	7.188264	10.19103	11.09838	8.979872	9.676813	9.534442	8.258427	**n.s.**

	TAC operation	Mouse heart	LV mass/BW ratio (mg/g)										One-way ANOVA
	Sham	WT	4.895561	4.372201	3.936856	5.613362	3.952081	4.731056					**n.s.**
Fig 4B	Sham	*Sirt5* KO	5.394938	4.920628	4.117754	4.460648	6.201299	6.575121	4.70715				**P<0.001**
	TAC	WT	11.06944	8.422363	8.958348	6.270921	9.197602	6.220322	8.168338	10.90375	9.219532		**P<0.01**
	TAC	*Sirt5* KO	5.346924	6.906968	6.511133	5.616544	7.947134	8.742394	6.936414	7.675686	7.095962	6.847018	**P<0.01**

	TAC operation	Mouse heart	LVAWTd (mm)										One-way ANOVA
	Sham	WT	0.93	0.97	0.88	1.15	0.78	0.83					**n.s.**
Fig 4C	Sham	*Sirt5* KO	0.99	1.06	0.78	1.1	1.11	0.96	0.87				**P<0.001**
	TAC	WT	1.54	1.46	1.47	1.08	1.63	1.41	1.35	1.72	1.43		**P<0.001**
	TAC	*Sirt5* KO	1.13	1.19	1.05	1.28	1.39	1.56	1.36	1.43	1.48	1.52	**n.s.**

	TAC operation	Mouse heart	LVAWTs (mm)										One-way ANOVA
	Sham	WT	1.19	1.46	1.25	1.48	1.34	1.26					**n.s.**
Fig 4D	Sham	*Sirt5* KO	1.43	1.43	1.19	1.61	1.56	1.52	1.33				**P<0.001**
	TAC	WT	2.01	1.72	1.87	1.6	1.93	2	1.99	2.01	1.92		**P<0.001**
	TAC	*Sirt5* KO	1.73	1.63	1.41	1.69	1.99	2.04	1.76	1.8	1.97	2	**n.s.**

	TAC operation	Mouse heart	LVPWTd (mm)										One-way ANOVA
	Sham	WT	0.85	0.78	0.81	1.15	0.78	0.83					**n.s.**
Fig 4E	Sham	*Sirt5* KO	0.73	0.62	0.74	0.81	1.11	0.96	0.87				**P<0.001**
	TAC	WT	1.46	1.17	1.14	1.11	1.41	1.41	1.35	1.72	1.43		**P<0.001**
	TAC	*Sirt5* KO	1.09	1.37	1.05	1.28	1.39	1.56	1.36	1.43	1.48	1.52	**n.s.**

Fig 4F	TAC operation	Mouse heart	LVPWTs (mm)										**One-way ANOVA**
	Sham	WT	1.19	1.35	1.03	1.48	1.34	1.26					**n.s.**
	Sham	*Sirt5* KO	0.95	0.84	0.96	1.14	1.56	1.52	1.33				**P<0.01**
	TAC	WT	1.76	1.57	1.36	1.3	1.63	2	1.99	2.01	1.92		**P<0.05**
	TAC	*Sirt5* KO	1.54	1.74	1.23	1.35	1.69	1.74	1.47	1.62	1.56	1.37	**n.s.**

Fig 4G	TAC operation	Mouse heart	Relative *Anp* mRNA expression										**One-way ANOVA**
	Sham	WT	1	3.396	4.379	2.766	0.74	1.09					**n.s.**
	Sham	*Sirt5* KO	1.226239	0.5378569	1.156	0.921	0.578	0.589	0.737				**P<0.01**
	TAC	WT	18.955	17.433	18.152	6.513	12.617	6.180084	5.474862	6.550071	4.140518		**P<0.01**
	TAC	*Sirt5* KO	2.811	18.915	7.873	0.903	8.013	9.319	13.878	23.424	8.78	13.107	**n.s.**

Fig 4H	TAC operation	Mouse heart	Relative *Bnp* mRNA expression										**One-way ANOVA**
	Sham	WT	1	1.8	8.182	3.325	1.108	0.897					**n.s.**
	Sham	*Sirt5* KO	1.942	3.853	0.91	1.296	0.427	1.424	1.45				**P<0.01**
	TAC	WT	17.572	15.576	10.97	4.396	7.598	9.886	5.328	6.186	5.109		**P<0.01**
	TAC	*Sirt5* KO	3.598	13.08	3.651	3.379	7.891	6.867	7.134	15.641	7.418	8.875	**n.s.**

Fig 4I	TAC operation	Mouse heart	Relative *Collagen 1a1* mRNA expression										**One-way ANOVA**
	Sham	WT	1	1.245	1.111	0.888	0.783	0.631					**n.s.**
	Sham	*Sirt5* KO	1.268395	0.4735532	0.547	0.717	0.799	0.764	0.545				**P<0.01**
	TAC	WT	2.941	1.972	2.721	1.796	2.707	1.575416	2.467485	2.889238	2.277246		**P<0.001**
	TAC	*Sirt5* KO	1.226	2.024	0.686	1.24	2.713	2.135	2.085	4.784	3.245	3.453	**n.s.**

Fig 4J	TAC operation	Mouse heart	Relative *Collagen 3a1* mRNA expression										**One-way ANOVA**
	Sham	WT	1	1.389	1.271	0.738	0.742	0.355					**n.s.**
	Sham	*Sirt5* KO	1.057486	0.4158356	0.462	0.702	1.003	1.017	0.503				**P<0.001**
	TAC	WT	4.518	2.804	4.455	2.069	4.269	2.050368	3.373918	3.622733	3.080355		**P<0.001**
	TAC	*Sirt5* KO	1.414	2.208	0.734	1.515	4.514	3.932	3.134	6.736	4.742	4.677	**n.s.**

Fig 5:

	TAC operation	Mouse heart	LVIDd (mm)										One-way ANOVA
Fig 5A	Sham	WT	4.08	3.92	3.77	4.18	3.93	3.9					**n.s.**
	Sham	*Sirt5* KO	4.21	4.06	4.08	3.92	4.12	4.01	3.83				**n.s.**
	TAC	WT	4.32	4.1	4.88	4.04	3.79	4.08	4.17	4.32	4.32		**n.s.**
	TAC	*Sirt5* KO	4.06	3.86	4.23	3.68	3.87	4.04	3.89	4.17	3.64	3.79	**P<0.05**

	TAC operation	Mouse heart	LVIDs (mm)										One-way ANOVA
Fig 5B	Sham	WT	2.71	2.47	2.78	3.11	2.63	2.89					**n.s.**
	Sham	*Sirt5* KO	3.04	2.86	3.04	2.56	2.93	2.82	2.77				**P<0.05**
	TAC	WT	3.26	3.3	4.26	3.01	2.97	2.89	2.82	3.59	3.27		**n.s.**
	TAC	*Sirt5* KO	2.78	2.82	3.11	2.52	2.59	3.01	2.68	3.2	2.15	2.41	**P<0.01**

	TAC operation	Mouse heart	LVEDV (μL)										One-way ANOVA
Fig 5C	Sham	WT	73.45	66.87	60.87	77.8	67.27	66					**n.s.**
	Sham	*Sirt5* KO	79.08	72.73	73.45	66.66	75.05	70.32	62.96				**n.s.**
	TAC	WT	84.04	74.29	111.94	71.88	61.38	73.45	77.4	84.04	84.15		**n.s.**
	TAC	*Sirt5* KO	72.54	64.29	79.8	57.55	64.81	71.88	65.6	77.4	55.77	61.38	**P<0.05**

	TAC operation	Mouse heart	LVESV (μL)										One-way ANOVA
Fig 5D	Sham	WT	27.24	21.58	29.12	38.18	25.43	31.96					**n.s.**
	Sham	*Sirt5* KO	36.14	31.04	36.24	23.76	33.08	30.08	28.65				**P<0.05**
	TAC	WT	42.83	44	81.23	35.17	34.11	32.06	30.07	54.03	43.2		**n.s.**
	TAC	*Sirt5* KO	29.09	30.08	38.14	22.75	24.47	35.17	26.54	40.83	15.33	20.42	**P<0.05**

	TAC operation	Mouse heart	LVEF%										One-way ANOVA
Fig 5E	Sham	WT	62.92	67.73	52.16	50.92	62.2	51.58					**n.s.**
	Sham	*Sirt5* KO	54.3	57.32	50.67	64.36	55.92	57.23	54.49				**P<0.05**
	TAC	WT	49.04	40.77	27.43	51.08	44.42	56.35	61.15	35.71	48.66		**n.s.**
	TAC	*Sirt5* KO	59.9	53.21	52.21	60.47	62.24	51.08	59.55	47.25	72.51	66.74	**P<0.05**

	TAC operation	Mouse heart	LVFS%										One-way ANOVA
Fig 5F	Sham	WT	33.64	37.14	26.21	25.66	33.02	25.93					**n.s.**
	Sham	*Sirt5* KO	27.83	29.73	25.45	34.58	28.83	29.63	27.72				**P<0.05**
	TAC	WT	24.58	19.64	12.78	25.69	21.57	29.09	32.43	16.95	24.35		**n.s.**
	TAC	*Sirt5* KO	31.48	26.92	26.5	31.63	33.01	25.69	31.13	23.42	40.82	36.27	**P<0.05**

Fig 6:

	TAC operation	Mouse heart	ATP abundance						One-way ANOVA
Fig 6A	Sham	WT	1471019	2002508.4	2065642	1374767.625	1936378.4	1869449.1	
	Sham	*Sirt5* KO	2475512	1517889.1	2167426.8	2490173.5	1354539	2191164.8	**n.s.**
	TAC	WT	1292508.1	2091684.5	3014372.3	1170712.25	2023285	2772744.3	
	TAC	*Sirt5* KO	714374.06	1787771.6	1465691.5	695169.375	1632497	1375292.1	**n.s.**

	TAC operation	Mouse heart	AMP abundance						One-way ANOVA
Fig 6B	Sham	WT	6601649.5	7680043.5	9736615	7045120.5	7910859.5	9648727	
	Sham	*Sirt5* KO	7456330	9327440	5130852	7670000.5	9745397	5441415.5	**n.s.**
	TAC	WT	5224542	7932034.5	4467941	5426371.5	8351821	4655911	
	TAC	*Sirt5* KO	5792616	10002278	8352540	5983945.5	10152843	8270800	**n.s.**

	TAC operation	Mouse heart	ADP abundance						One-way ANOVA
Fig 6C	Sham	WT	4684746	6143032.5	4786874.5	4536606.5	5880431	4303939.5	
	Sham	*Sirt5* KO	5352461	4737500.5	3463169.5	5306581	4218373.5	3603430.3	**n.s.**
	TAC	WT	4261387	5742245.5	4160223	3856204	5482715.5	4162542.8	
	TAC	*Sirt5* KO	3004907.8	4043106	4077452.8	2884124.75	4128711.8	3957854.5	**n.s.**

	TAC operation	Mouse heart	AMP/ATP ratio						One-way ANOVA
Fig 6D	Sham	WT	4.4878071	3.8352117	4.7136024	5.124590056	4.0853893	5.1612675	
	Sham	*Sirt5* KO	3.0120355	6.1450075	2.3672551	3.080106868	7.1946227	2.4833438	**n.s.**
	TAC	WT	4.0421734	3.7921754	1.4822128	4.635102691	4.127852	1.6791707	
	TAC	*Sirt5* KO	8.1086595	5.5948298	5.6987026	8.607895738	6.2192108	6.0138496	**P<0.01**

	TAC operation	Mouse heart	p-AMPK/AMPK ratio			One-way ANOVA
Fig 6F	Sham	WT	1	1.09	0.54	
	Sham	*Sirt5* KO	0.56	1.89	0.84	**n.s.**
	TAC	WT	0.96	0.58	0.63	
	TAC	*Sirt5* KO	1.35	1.25	1.23	**n.s.**

Fig 6G	TAC operation	Mouse heart	p-ACC/ACC ratio			**One-way ANOVA**
	Sham	WT	1	1.15	0.87	
	Sham	*Sirt5* KO	0.86	1.19	1.2	**n.s.**
	TAC	WT	1.01	0.99	0.8	
	TAC	*Sirt5* KO	1.31	1.83	1.56	**P<0.05**

Fig 6H	TAC operation	Mouse heart	p-4EBP1/4EBP1 ratio			**One-way ANOVA**
	Sham	WT	1	2.458518	2.267091	
	Sham	*Sirt5* KO	3.426408	3.162408	2.543768	**n.s.**
	TAC	WT	5.377864	3.504437	4.861915	
	TAC	*Sirt5* KO	2.086624	2.17425	2.134938	**P<0.01**

S10 Fig:

	Cell line	ATP abundance			T.TEST
S10A Fig	shScramble	3.62	3.56	3.59	
	sh*SIRT5*-#1	1.5	1.81	2.01	**0.000262**
	sh*SIRT5*-#2	2.73	2.52	2.74	**0.000231**

	Cell line	AMP/ATP ratio			T.TEST
S10B Fig	shScramble	0.1851152	0.1546407	0.1573987	
	sh*SIRT5*-#1	0.8185163	0.5416455	0.5353826	**0.007671**
	sh*SIRT5*-#2	0.4591781	0.5085841	0.5513567	**0.000275**

	Cell line	ATP abundance			T.TEST
S10D Fig	WT	21	24.4	29	
	SIRT5 KO pool	16.9	17.4	20.4	**0.037948**

	Cell line	AMP/ATP ratio			T.TEST
S10E Fig	WT	0.2198541	0.2317123	0.3068454	
	SIRT5 KO pool	0.4879515	0.4882012	0.3628273	**0.017808**

S13 Fig:

S13A Fig		Relative ATP synthase activity						
	Mouse heart mitochondria	20 µg	10 µg	5 µg	2.5 µg	1 µg	1 µg + OL	Reaction buffer
	Rate (mOD/min)	42.6	34	38.53846154	26.972973	16.071429	0.3666667	0

S13B Fig		Relative ATP synthase activity		
	Mouse heart mitochondria	2.5 µg	2.5 µg + OL	Reaction buffer
	Rate (mOD/min)	39.86666667	1.203389831	0

S14 Fig:

S14A Fig	Mouse heart mitochondria	Relative Sdha protein level								
	WT	4.8278199	4.5148088	4.0376265	4.1463167	4.4340019	5.0464961			
	Sirt5 KO	4.5509137	5.2370692	5.0870962	5.7342472	4.3739034	5.6469513	6.2202445	5.5277456	5.2489015

S14B Fig	Mouse heart mitochondria	Relative ATP synthase activity												
	WT	0.9785343	0.6905931	0.7766795	1.1639486	1.1633299	0.9267171	1.1265443	0.8078657	1.0588162	1.297986	1.2437013	1.010917	1.5153201
	Sirt5 KO	1.0997907	0.902131	0.9761469	0.8351914	0.8688806	0.7634696	1.0709346	0.7108738	0.8435178	1.008214	0.5470215	0.888114	1.3791742

S14B Fig	Mouse heart mitochondria	Relative ATP synthase activity												
	WT	1.0548111	0.8315074	0.7233247	0.8976672	0.7317367								
	Sirt5 KO	0.7500326	0.7493869	0.6119907	0.6219108	0.8189037	0.622548	0.5721879	0.6316023	0.7769911	0.7062384	0.6266004	0.8105686	0.8799768

S14B Fig	Mouse heart mitochondria	Relative ATP synthase activity	**T.TEST**
	WT		
	Sirt5 KO	0.711741	**0.003182**

Table 1:

TAC operation	Mouse heart	Heart Rate (bpm)										**One-way ANOVA**
Sham	WT	565	539	500	578	558	545					**n.s.**
Sham	*Sirt5* KO	585	444	585	563	495	462	527				**n.s.**
TAC	WT	516	511	539	516	522	516	457	578	490		
TAC	*Sirt5* KO	485	581	432	545	533	466	466	578	466	495	**n.s.**

TAC operation	Mouse heart	Peak flow velocity (mm/s)										**One-way ANOVA**
Sham	WT	873.71	711.21	879.75	696.09	ND	ND					**P<0.001**
Sham	*Sirt5* KO	920.36	752.73	924.65	823.47	808.11	ND	ND				**P<0.001**
TAC	WT	3437.81	3427.63	3403.78	3420.99	3390.38	3413.53	3408.46	ND	ND		
TAC	*Sirt5* KO	3395.04	3434.45	3154.09	3427.63	3387.31	3451.35	3410.74	3403.78	3414.19	ND	**n.s.**
Note: The values were forgotten to be determined (ND).												

TAC operation	Mouse heart	LVAWTd (mm)										**One-way ANOVA**
Sham	WT	0.93	0.97	0.88	1.15	0.78	0.83					**P<0.001**
Sham	*Sirt5* KO	0.99	1.06	0.78	1.1	1.11	0.96	0.87				**P<0.001**
TAC	WT	1.54	1.46	1.47	1.08	1.63	1.41	1.35	1.72	1.43		
TAC	*Sirt5* KO	1.13	1.19	1.05	1.28	1.39	1.56	1.36	1.43	1.48	1.52	**n.s.**

TAC operation	Mouse heart	LVAWTs (mm)										**One-way ANOVA**
Sham	WT	1.19	1.46	1.25	1.48	1.34	1.26					**P<0.001**
Sham	*Sirt5* KO	1.43	1.43	1.19	1.61	1.56	1.52	1.33				**P<0.001**
TAC	WT	2.01	1.72	1.87	1.6	1.93	2	1.99	2.01	1.92		
TAC	*Sirt5* KO	1.73	1.63	1.41	1.69	1.99	2.04	1.76	1.8	1.97	2	**n.s.**

TAC operation	Mouse heart	LVPWTd (mm)										**One-way ANOVA**
Sham	WT	0.85	0.78	0.81	1.15	0.78	0.83					**P<0.001**
Sham	*Sirt5* KO	0.73	0.62	0.74	0.81	1.11	0.96	0.87				**P<0.001**
TAC	WT	1.46	1.17	1.14	1.11	1.41	1.41	1.35	1.72	1.43		
TAC	*Sirt5* KO	1.09	1.37	1.05	1.28	1.39	1.56	1.36	1.43	1.48	1.52	**n.s.**

TAC operation	Mouse heart	LVPWTs (mm)										**One-way ANOVA**
Sham	WT	1.19	1.35	1.03	1.48	1.34	1.26					**P<0.01**
Sham	*Sirt5* KO	0.95	0.84	0.96	1.14	1.56	1.52	1.33				**P<0.05**
TAC	WT	1.76	1.57	1.36	1.3	1.63	2	1.99	2.01	1.92		
TAC	*Sirt5* KO	1.54	1.74	1.23	1.35	1.69	1.74	1.47	1.62	1.56	1.37	**n.s.**

TAC operation	Mouse heart	LVIDd (mm)										**One-way ANOVA**
Sham	WT	4.08	3.92	3.77	4.18	3.93	3.9					**n.s.**
Sham	*Sirt5* KO	4.21	4.06	4.08	3.92	4.12	4.01	3.83				**n.s.**
TAC	WT	4.32	4.1	4.88	4.04	3.79	4.08	4.17	4.32	4.32		
TAC	*Sirt5* KO	4.06	3.86	4.23	3.68	3.87	4.04	3.89	4.17	3.64	3.79	**P<0.05**

TAC operation	Mouse heart	LVIDs (mm)										**One-way ANOVA**
Sham	WT	2.71	2.47	2.78	3.11	2.63	2.89					**P<0.05**
Sham	*Sirt5* KO	3.04	2.86	3.04	2.56	2.93	2.82	2.77				**n.s.**
TAC	WT	3.26	3.3	4.26	3.01	2.97	2.89	2.82	3.59	3.27		
TAC	*Sirt5* KO	2.78	2.82	3.11	2.52	2.59	3.01	2.68	3.2	2.15	2.41	**P<0.01**

TAC operation	Mouse heart	LVEDV (µl)										**One-way ANOVA**
Sham	WT	73.45	66.87	60.87	77.8	67.27	66					**n.s.**
Sham	*Sirt5* KO	79.08	72.73	73.45	66.66	75.05	70.32	62.96				**n.s.**
TAC	WT	84.04	74.29	111.94	71.88	61.38	73.45	77.4	84.04	84.15		
TAC	*Sirt5* KO	72.54	64.29	79.8	57.55	64.81	71.88	65.6	77.4	55.77	61.38	**P<0.05**

TAC operation	Mouse heart	LVESV (µl)										**One-way ANOVA**
Sham	WT	27.24	21.58	29.12	38.18	25.43	31.96					**P<0.05**
Sham	*Sirt5* KO	36.14	31.04	36.24	23.76	33.08	30.08	28.65				**n.s.**
TAC	WT	42.83	44	81.23	35.17	34.11	32.06	30.07	54.03	43.2		
TAC	*Sirt5* KO	29.09	30.08	38.14	22.75	24.47	35.17	26.54	40.83	15.33	20.42	**P<0.05**

TAC operation	Mouse heart	Stroke Volume (µl)										**One-way ANOVA**
Sham	WT	46.21	45.29	31.75	39.62	41.84	34.04					**n.s.**
Sham	*Sirt5* KO	42.94	41.69		42.9	41.97	40.24	34.31				**n.s.**
TAC	WT	41.21	30.29	30.71	36.71	27.27	41.39	47.33	30.01	40.95		
TAC	*Sirt5* KO	43.45	34.21	41.66	34.8	40.34	36.71	39.06	36.57	40.44	40.96	**n.s.**

TAC operation	Mouse heart	LVEF (%)										**One-way ANOVA**
Sham	WT	62.92	67.73	52.16	50.92	62.2	51.58					**P<0.05**
Sham	*Sirt5* KO	54.3	57.32	50.67	64.36	55.92	57.23	54.49				**n.s.**
TAC	WT	49.04	40.77	27.43	51.08	44.42	56.35	61.15	35.71	48.66		
TAC	*Sirt5* KO	59.9	53.21	52.21	60.47	62.24	51.08	59.55	47.25	72.51	66.74	**P<0.05**

TAC operation	Mouse heart	LVFS (%)										**One-way ANOVA**
Sham	WT	33.64	37.14	26.21	25.66	33.02	25.93					**P<0.05**
Sham	*Sirt5* KO	27.83	29.73	25.45	34.58	28.83	29.63	27.72				**n.s.**
TAC	WT	24.58	19.64	12.78	25.69	21.57	29.09	32.43	16.95	24.35		
TAC	*Sirt5* KO	31.48	26.92	26.5	31.63	33.01	25.69	31.13	23.42	40.82	36.27	**P<0.05**

TAC operation	Mouse heart	LV Mass Corrected (mg)										**One-way ANOVA**
Sham	WT	112.5	103.49	91.65	130.23	94.02	76.17					**P<0.001**
Sham	*Sirt5* KO	112.97	103.53	90.92	115.62	124.15	122.1	89.53				**P<0.001**
TAC	WT	263.01	199.61	255.94	148.37	222.49	185.49	195.55	273.03	224.68		
TAC	*Sirt5* KO	152.12	174.47	131.59	137.83	178.89	221.27	151.63	204.25	149.37	158.44	**P<0.001**
